# Symmetric subgenomes and balanced homoeolog expression stabilize the establishment of allopolyploidy in cyprinid fish

**DOI:** 10.1186/s12915-022-01401-4

**Published:** 2022-09-14

**Authors:** Li Ren, Xin Gao, Jialin Cui, Chun Zhang, He Dai, Mengxue Luo, Shaofang He, Qinbo Qin, Kaikun Luo, Min Tao, Jun Xiao, Jing Wang, Hong Zhang, Xueyin Zhang, Yi Zhou, Jing Wang, Xin Zhao, Guiming Liu, Guoliang Wang, Linhe Huo, Shi Wang, Fangzhou Hu, Rurong Zhao, Rong Zhou, Yude Wang, Qinfeng Liu, Xiaojing Yan, Chang Wu, Conghui Yang, Chenchen Tang, Wei Duan, Shaojun Liu

**Affiliations:** 1 State Key Laboratory of Developmental Biology of Freshwater Fish, College of Life Sciences, Hunan Normal University, Changsha, 410081 China; 2 Biomarker Technologies Corporation, Beijing, 101300 China; 3 Wuhan Carbon Code Biotechnologies Corporation, Wuhan, 430070 China; 4 Beijing Agro-Biotechnology Research Center, Beijing Academy of Agriculture and Forestry Sciences, Beijing, 100097 China

**Keywords:** Polyploid, Hybridization, Genomic recombination, *Cis*- and *trans*- regulation, Fish

## Abstract

**Background:**

Interspecific postzygotic reproduction isolation results from large genetic divergence between the subgenomes of established hybrids. Polyploidization immediately after hybridization may reset patterns of homologous chromosome pairing and ameliorate deleterious genomic incompatibility between the subgenomes of distinct parental species in plants and animals. However, the observation that polyploidy is less common in vertebrates raises the question of which factors restrict its emergence. Here, we perform analyses of the genome, epigenome, and gene expression in the nascent allotetraploid lineage (2.95 Gb) derived from the intergeneric hybridization of female goldfish (*Carassius auratus*, 1.49 Gb) and male common carp (*Cyprinus carpio*, 1.42 Gb), to shed light on the changes leading to the stabilization of hybrids.

**Results:**

We firstly identify the two subgenomes derived from the parental lineages of goldfish and common carp. We find variable unequal homoeologous recombination in somatic and germ cells of the intergeneric F_1_ and allotetraploid (F_22_ and F_24_) populations, reflecting high plasticity between the subgenomes, and rapidly varying copy numbers between the homoeolog genes. We also find dynamic changes in transposable elements accompanied by genome merger and duplication in the allotetraploid lineage. Finally, we observe the gradual decreases in *cis*-regulatory effects and increases in *trans*-regulatory effects along with the allotetraploidization, which contribute to increases in the symmetrical homoeologous expression in different tissues and developmental stages, especially in early embryogenesis.

**Conclusions:**

Our results reveal a series of changes in transposable elements, unequal homoeologous recombination, *cis*- and *trans*-regulations (e.g. DNA methylation), and homoeologous expression, suggesting their potential roles in mediating adaptive stabilization of regulatory systems of the nascent allotetraploid lineage. The symmetrical subgenomes and homoeologous expression provide a novel way of balancing genetic incompatibilities, providing a new insight into the early stages of allopolyploidization in vertebrate evolution.

**Supplementary Information:**

The online version contains supplementary material available at 10.1186/s12915-022-01401-4.

## Background

Two rounds of ancient polyploidization events are inferred to have occurred during the evolution from fish to mammals [1, 2]. The benefits of polyploidization are attributed to the acceleration of species diversity by breaking post-zygotic reproductive isolation, dosage effects, the sub- or neo-functionalization of duplicated genes and increased phenotypic variation [3]. Polyploidization is currently rarely observed in animals but is regularly observed in plants [4, 5]. Traditional explanations for this disparity are related to special sexual reproduction, physiological and developmental constraints [4, 6], transposon divergences [5], genome incompatibility and remodelling [7], and failure of homologous chromosome pairing during meiosis [8]. One type of polyploidization in animals resulting from unreduced gametes in hybrid progenies can reverse the reduction in fertility arising from hybrid incompatibility [7, 9]. However, increased gene copy numbers after polyploidization disrupt regulatory systems and result in the death of individuals [10]. To date, the factors in evolutionarily divergent genomes that contribute to establishing stable regulatory systems in allopolyploid lineages have yet to be clearly elucidated in vertebrates.

The genomic shock resulting from allopolyploidization induces various changes at the genomic (e.g. DNA recombination), epigenetic, and gene expression (e.g. transcriptomic shock) levels in some plant systems, such as in Brassica [11], rice [12], and cotton [13]. The rare identification of nascent allopolyploids in vertebrates has resulted in confusion about which genetic characteristics are necessary in hybrid individuals to achieve effective polyploidization. The merger of divergent genomes results in rapid and widespread genetic changes, including novel patterns of *cis*- and *trans*-regulations shaped by the interactions of divergent or conserved homoeologs [14]. On the other hand, the emergence of unreduced gametes may be related to some genetic changes existing in diploid hybrids before polyploidization. Analyses of the separate contributions of hybridization and polyploidization may allow this question to be effectively investigated and addressed in vertebrates.

A nascent allotetraploid lineage obtained from the intergeneric hybridization of goldfish (*Carassius auratus*) and common carp (*Cyprinus carpio*) is an appropriate model for addressing the above questions. Goldfish and common carp share a common allopolyploidization event that occurred approximately 13.75 million years ago (Mya), and these species diverged approximately 9.95 Mya [7, 15, 16]. Their wide distributions and diverse phenotypes, including the sizes and colours of their bodies, fins, and eyes [17–19], are related to their high-level genomic plasticity. Furthermore, bisexual sterile triploids have been obtained via the interploid crossing of the allotetraploid with its inbred parents [20, 21]. The characteristics of fast growth and strong innate immunity have been targets of commercial fish breeding in the Yangtze drainage basin [22]. To investigate the origin of polyploidization in the allotetraploid, we aimed to (i) assemble its genome into chromosome-scale sequences via multiple sequencing strategies; (ii) detect potential genomic changes in the allotetraploid lineage; and (iii) disentangle the separate contributions of hybridization, polyploidization, and/or biological inheritance to the changes in *cis*- and *trans*-regulations (e.g. DNA methylation) and gene expression at the homoeolog level.

## Results

### High-quality genome assembly and annotation in the allotetraploid

**Fig. 1:**
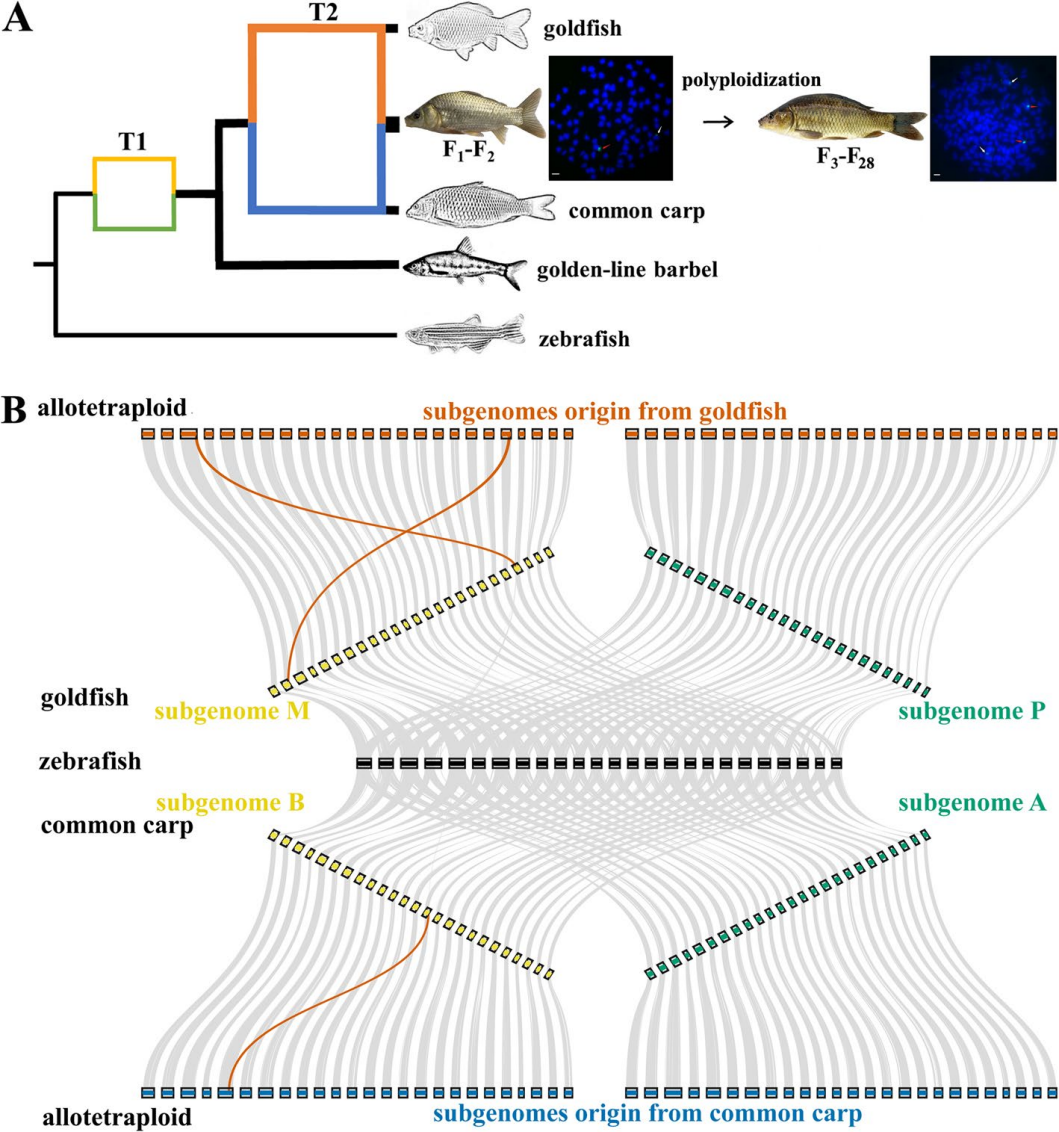
Two rounds of allopolyploidization and subgenome determination. **A** Simulated topologies of phylogeny reflecting the origin of goldfish, common carp, and their allotetraploid progenies. Polyploidization occurring in F_3_ individuals gave rise to the allotetraploid lineage of goldfish and common carp [7, 23]. The width of the lines represents the genome size of the species. The maternal ancestor (yellow) and paternal ancestor (green) of goldfish, common carp, and golden-line barbel (*Sinocyclocheilus grahami*) existed for a short time span (T1) relative to the divergence time between goldfish and common carp (T2). One strong (red, originating from goldfish) and one weak (white, originating from common carp) signal were detected in the intergeneric F_1_ using fluorescence in situ hybridization, while two strong (red) and two weak (white) signals were observed in allotetraploid F_22_ and F_24_ (Scale bar: 3 cm) [24]. **B** Genome synteny of goldfish, common carp, and their allotetraploid progenies. Blocks represent the assembled chromosomes of subgenome R (red) and subgenome C (blue) in the allotetraploid, subgenome M in goldfish and subgenome B in common carp (yellow river carp) (originating in the maternal ancestor, yellow), and subgenome P in goldfish and subgenome A in common carp (originating in the paternal ancestor, green). The red line represents the three interchromosomal translocations between the inbred parents and the corresponding subgenome of the assembled genome

To determine the genetic background of the allotetraploid lineage of goldfish (♀) and common carp (♂), we identified 100 chromosomes in the intergeneric F_1_ and 200 chromosomes in the allotetraploid F_22_ and F_24_ based on metaphase chromosome assays of cultured kidney cells. Using fluorescence in situ hybridization, it was discovered that half of their chromosomes originated from goldfish and the other half from common carp (Fig. 1A). The high-quality (contig N50: 2.86 Mb, scaffold N50: 26.74 Mb) chromosome-level genome assembly of the nascent allotetraploid facilitated subsequent analyses (Table 1, Additional files 1 and 2: Tables S1-S11 and Additional file 1: Fig. S1). For the allotetraploid genome assembly, 4202 contigs were merged into 2812 scaffolds, of which 91.72% were anchored to 100 pseudo-chromosomes. The genome size (2.95 Gb) and chromosome number (2n: 200) of the allotetraploid were close to the combined values from the parental goldfish (1.49 Gb, 2n: 100) and common carp (1.42 Gb, 2n: 100) (Fig. 1A). The high level of complete BUSCOs (96.42%) indicated the completeness of the assembled allotetraploid genome (Additional file 1: Table S12).

**Table 1:** Overview of assembly genome for the allotetraploid fish

	Scaffold	Contig
Number	2812	4202
Total length (bp)	2,953,563,945	2,953,424,945
N50 (bp)	26,740,681	2,863,522
N90 (bp)	18,507,213	473,835
Max length (bp)	47,189,010	13,873,778
Length of scaffolds anchored on linkage groups	2.71 Gb (91.72%)	/
Complete BUSCOs	3234 (96.42%)	/
Gene number	85,214	/
Repeat sequences (bp)	1,289,350,257 (43.65%)	/
Simple sequence repeats (bp)	193,607,232 (6.55%)	/

Genome annotation allowed identification of 86,180 protein-coding genes in the allotetraploid genome (Additional file 1: Tables S13-S14); gene features were similar to those of the two inbred parents (Additional file 1: Fig. S2). Transposable elements (TEs) accounted for 1.29 Gb of the genome (43.65%). In addition, 193.6 Mb of tandem repeats were found in the allotetraploid genome (6.57%) (Additional file 1: Tables S15-S16). Finally, a total of 50,493 non-coding RNAs were annotated in the allotetraploid genome (Additional file 1: Table S17).

The observation of high synteny and collinearity among zebrafish, goldfish, common carp, and the allotetraploid fish reflected an additional allopolyploidization event from zebrafish to goldfish or common carp (13.75 Mya), and a subsequent allopolyploidization event from goldfish or common carp to the nascent allotetraploid fish based on 13,244 conserved homologous gene pairs (Fig. 1A, B) [16]. High synteny and collinearity were also observed between the chromosomes of the parental goldfish and common carp of the allotetraploid fish (grey line in Additional file 1: Fig. S3). Additionally, the high symmetry between two sets of ancestral chromosomes reflected parallel evolution in goldfish and common carp, respectively (black line in Additional file 1: Fig. S3).

### Transposable elements change along with hybridization and polyploidization

We next sought to address the question of which genomic characteristics of goldfish and common carp affect the polyploidization of the nascent allotetraploid. Therefore, we analysed the genomic divergence between the inbred parental genomes in the allotetraploid lineage, an intergeneric hybrid of *Culter alburnus* and *Megalobrama amblycephala* (2n: 48) [25], and three interspecific hybrids of *Takifugu rubripes* and *Takifugu flavidus* (2n: 44) [26, 27], *Oreochromis aureus* and *Oreochromis niloticus* (2n: 48) [28, 29], and *Xiphophorus hellerii* and *Xiphophorus maculatus* (2n: 48) [30, 31] (Additional file 1: Tables S18-S21).

**Fig. 2.**
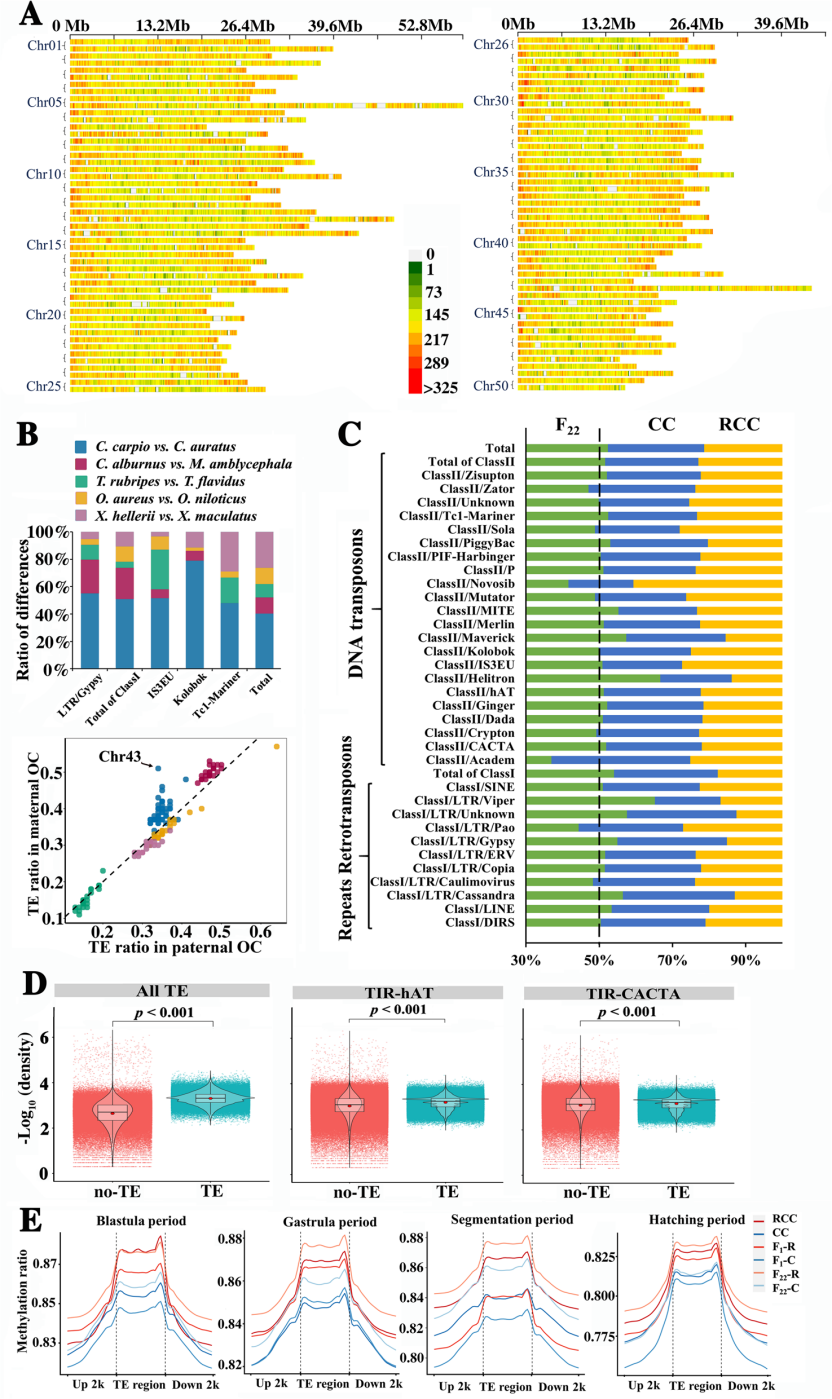
TE changes accompanying allopolyploidization and their correlation with structural variation and DNA methylation. **A** The density of TEs in OCPs between common carp (the former in each pair) and goldfish (the latter in each pair). **B** The percentage represents the differences in TEs between the two inbred parents of hybrids (left figure). TE differences were detected in each OCP (right figure). **C** Repeat sequence lengths among the allotetraploid, goldfish, and common carp. Red numbers represent a higher percentage (> 50%) in the allotetraploid than in the combination of the two inbred parents, while black numbers represent a lower percentage (< 50%). **D** Structural variation frequency in regions of TEs (< 1 kb) and no-TE. Correlation relationship between the distribution of TEs and structural variations in the allotetraploid (*p* < 0.001, *t* test). **E** Methylation levels (MLs) of homoeologs around TE regions in four developmental stages of goldfish, common carp, F_1_, and F_22_. “Up 2k” represents the 2 kb upstream region. “Down 2k” represents the 2 kb downstream region.

Focusing on the two parental genomes of each hybrid lineage, the percentage of orthologous genes in each orthologous chromosome pair and across all chromosomes was found to be lower in the parental genomes of the allotetraploid (39.83% in common carp and 41.19% in goldfish) than in the other four interspecific hybrid and/or intergeneric hybrid lineages (44.61%~92.98%) (Fig. 2A, Additional files 3 and 4: Tables S22-S23 and Additional file 1: Fig. S4-S5). The distribution of synonymous substitutions (K_s_) and the ratio of K_s_ to nonsynonymous substitutions (K_a_) (K_a_/K_s_) showed the highest genetic diversity within coding sequences between the two paternal genomes of the allotetraploid lineage relative to the others (Additional file 1: Fig. S6). The differences in other genomic characteristics between the two parental genomes, including microsatellites (0.65%), some categories of TEs (1.62% for LTR-Gypsy, 0.16% for IS3EU, 0.34% for Kolobok transposons, 0.33% for Tc1-Mariner; 3.63% in total of repeat retrotransposons and 2.64% in total of TEs), and the TEs present in each orthologous chromosome pair (OCP), were also greater in the allotetraploid lineage than in the other four lineages (Fig. 2A, B, Additional file 1: Table S24 and Figs. S7-S11, Additional file 5: Table S25). These genomic characteristics, including dynamic differences in transposable elements, revealed that genomic divergence was higher in the inbred parental genomes of the allotetraploid lineage than in those of the other four lineages.

A greater number of TEs were detected in goldfish (649.51 Mb) and common carp (526.81 Mb) than in the parental genomes of the other four lineages (Additional file 5: Table S25). Meanwhile, the numbers of TEs (1.29 Gb, 43.65%) and tandem repeats (193.61 Mb, 6.57%) in the allotetraploid were higher than the combined values from the two inbred parents (Additional file 1: Tables S15-S16). Most TEs (26 out of 36) had a higher percentage in the allotetraploid (> 50%) than the combined values from the two inbred parents, especially the “Helitron” and “Viper” types (> 60%), whereas the percentages of 10 TEs were lower in the allotetraploid (50%), including the “Novosib” and “Academ” types (42%) (Fig. 2C). The large number of TEs and their dramatic changes from goldfish or common carp to the allotetraploid fish may contribute to the hybridization and polyploidization events of the nascent allotetraploid.

### Rapid emergence of unequal homoeologous recombination balancing subgenome conflicts

**Fig. 3:**
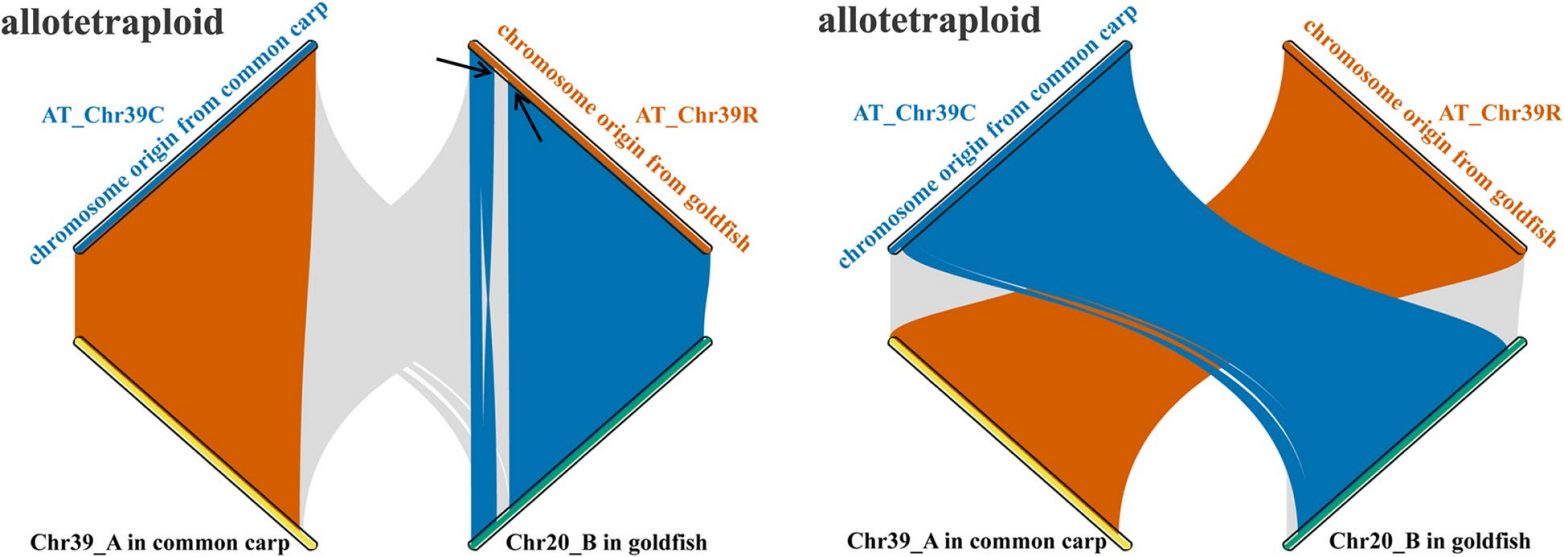
Determination of unequal HR in the allotetraploid. Gene synteny analyses of homoeologous chromosome pairs (HCPs) in the allotetraploid and corresponding orthologous chromosome pairs (OCPs) in the inbred parents revealed unequal exchange of homoeologous chromosomes in the assembled genome (3,426,224-6,057,586 bp in chromosome chr39R of the allotetraploid, black arrow)

A number of genomic variations, including three events of translocation between non-homologous chromosomes, were detected in the assembled genome based on comparison with the inbred parents (Fig. 1B and Additional file 1: Fig. S12). Additionally, unequal homoeologous recombination (HR) was observed in chromosome 39 (chr39) of the assembled genome, in which the 48 contiguous genes of subgenome R (originating from goldfish) were replaced with homoeologous sequences from subgenome C (originating from common carp) (Fig. 3A).

Analyses of whole-genome resequencing data revealed various unequal HR events in the intergeneric F_1_ (81~111), F_22_ allotetraploid (84~118), and F_24_ allotetraploid (163~369) populations (Additional file 1: Tables S26-S27). Unequal HR events involving three contiguous genes (16~54 in F_1_, 15~20 in F_22_ and 25~172 in F_24_) were mainly concentrated on chr34 in F_1_; chr14, chr25, and chr38 in F_22_; and chr19, chr31, chr34, and chr35 in F_24_ (Fig. 3B, C). These results indicated that the occurrence of unequal HR was induced not only by hybridization (F_0_~F_1_) but also by polyploidization (F_2_~F_3_) and transgenerational inheritance (F_3_~F_22_/F_24_). Interestingly, different distributions of unequal HR were observed between somatic and germ cells in the same individual.

Unequal HR and its effects on homoeologous expression in the caudal fin were detected in five allotetraploid individuals (F_24_) (Fig. 3C). Interestingly, the bias of homoeolog expression was not completely consistent with the ratio of gene copies between the homoeologs. The emergence of copy number changes between R and C homoeologs in hybrids could decrease the expression divergence originating from their inbred parents. Furthermore, whole-genome resequencing results showed that the emergence of structural variations was positively correlated with the distribution of DNA transposons of the CACTA and hAT superfamilies in the allotetraploid population, while negative correlations were detected between structural variations and other TE categories (*p* < 0.001, *t* test) (Fig. 2D and Additional file 1: Fig. S14 and Table S28). Meanwhile, the lengths of CACTA and hAT DNA transposons in the allotetraploid were greater than the sum of the lengths in the inbred parents (Fig. 2C). These rapid changes in the CACTA and hAT superfamilies reflect a potential role in the process of polyploidization.

### DNA methylation changes regulate homoeolog expression

**Fig. 4:**
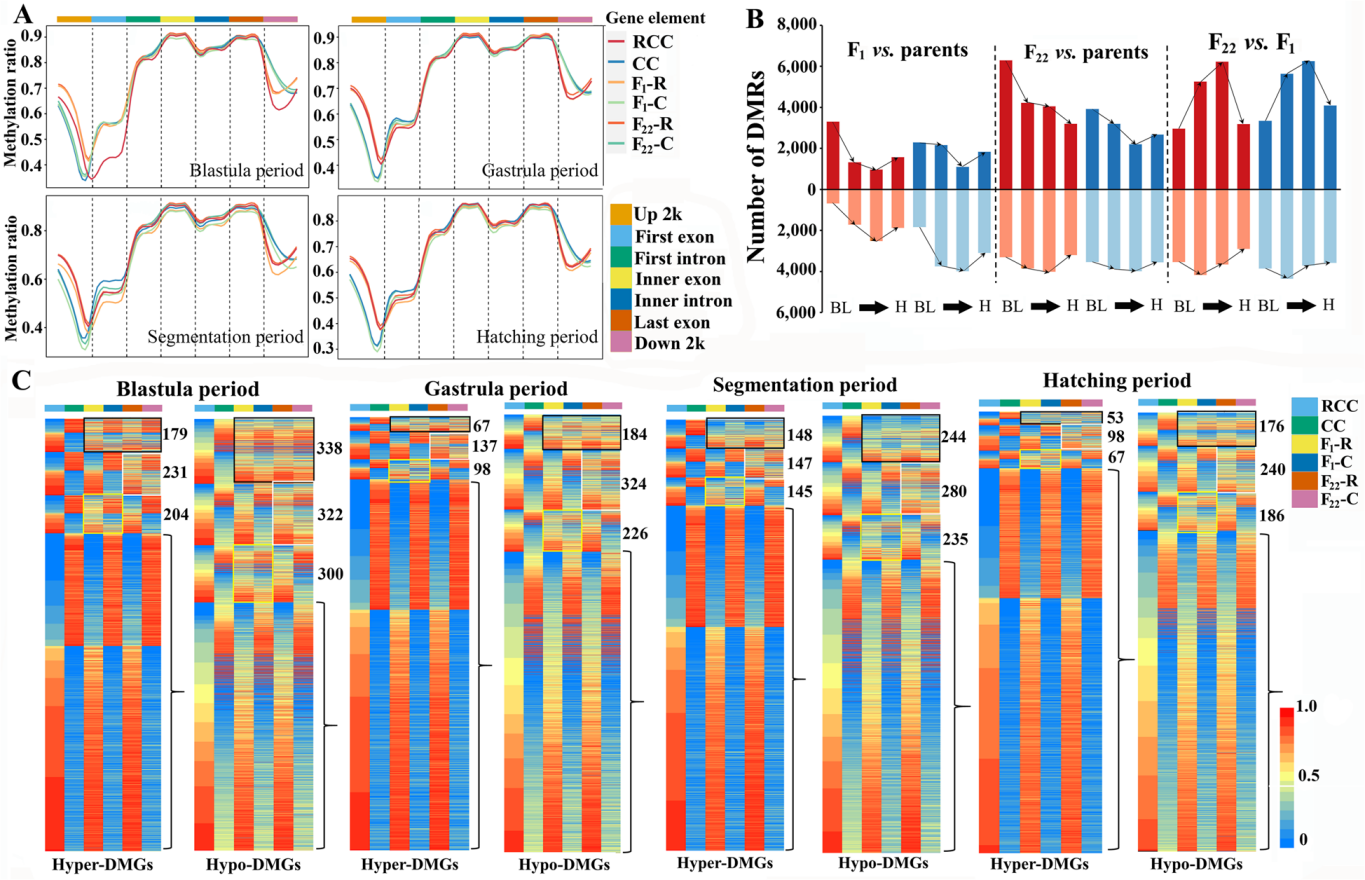
DNA methylation changes accompanying allopolyploidization. **A** The methylation ratios between subgenomes R and C in the four periods were observed in “up 2k” (2 kb upstream of TSS), gene body, and “down 2k” (2 kb downstream of TTS) regions. **B** Comparative analyses of DMRs among the inbred parents (goldfish and common carp), F_1_, and F_22_. In subgenome R, dark red represents the number of genes with higher methylation levels in the first group, while light red represents those with higher methylation levels in the second group. In subgenome C, dark blue represents the number of genes with higher methylation levels in the first group, while light blue represents those with higher methylation levels in the second group. The “BL to H” represents the four embryo development stages in order of BL, G, S, and H periods. **C** Differential methylation (DM) analysis between the two inbred parents revealed hyper-DMGs (DM > 0.6) and hypo-DMGs (DM < 0.6). Some of these genes (black box) exhibit inherited methylation changes during allopolyploidization. Some genes (yellow box) exhibit hybridization-induced and polyploidization-recovered methylation changes. Some genes (white box) show polyploidization-induced methylation changes. Brackets indicate no DM between the two inbred parents and the two hybrids

After obtaining DNA methylation levels in the four periods of blastula, gastrula, segmentation, and hatching, differentially methylated analysis among goldfish, common carp, F_1_, and F_22_ showed that the methylation changes resulting from allopolyploidization and transgenerational inheritance were mainly enriched in the regions 2 kb upstream of the transcription start site (TSS) and downstream of the transcription termination site (TTS) (Fig. 4A and Additional file 1: Tables. S29-S31). Interestingly, DNA methylation diversity between R and C homoeologs decreased gradually in the 2 kb upstream of the TSS but increased in the 2 kb downstream of the TTS, accompanied by allopolyploidization and transgenerational inheritance (*t-*test, Additional file 1: Fig. S15).

Differentially methylated regions (DMRs) (2 kb upstream of the TSS) were more in the F_1_ and F_22_ comparison (66,634), which involves potential effects in the processes of polyploidization and transgenerational inheritance, than in the two comparisons (the inbred parents *vs.* F_1_ (33,940) and F_22_ (59,008, respectively) (Fig. 4B). We detected R or C homoeologs in the F_1_ *vs.* F_22_ comparison independently. The number of DMRs in the C homoeolog was higher than in the R homoeolog, reflecting that the larger methylation changes occurred in the C homoeolog accompanied by polyploidization. Additionally, the MLs of species-specific genes (SSGs) originating from goldfish or common carp were similar between F_1_ or F_22_ and their inbred parents, except that the ones originating from common carp in the segmentation and hatching periods were lower in F_1_ than in common carp and F_22_ (Additional file 1: Fig. S16). These results indicated that some DNA methylation changes accompanied by hybridization were recovered by polyploidization and transgenerational inheritance (yellow box, Fig. 4C) [32].

Allopolyploidization and transgenerational inheritance had diverse effects on MLs in different developmental stages (Fig. 4 and Additional file 1: Fig. S17). In the blastula period, the greatest number of DMRs was found in the “F_22_ *vs.* parents” comparison, revealing that obvious changes in DNA methylation were made during the maternal-to-zygotic transition, in which the existence of maternal transcripts of C homoeolog in the allotetraploid had dramatic effects on zygotic genome activation. Then, in gastrula and segmentation periods, the greatest number of DMRs was found in “F_22_ *vs.* F_1_”, reflecting that the large-scale methylation changes in these two developmental stages may be related to the increase in gene copies (Fig. 4B and C). Functional analyses showed that 155 differentially methylated genes (DMGs) (yellow and white boxes) were enriched in neural development (ko05012: Parkinson’s disease; ko05016: Huntington’s disease) (Fig. 4C and Additional file 1: Fig. S18). Twenty-one DMGs were detected in pathways of central carbon metabolism.

Our results further revealed inherited methylation changes in some DMGs accompanied by allopolyploidization and transgenerational inheritance (black box, Fig. 4C). Overall, the identified methylation changes were evenly distributed on each chromosome of F_1_ and F_24_ (Additional file 1: Figs. S19-S22). Analysis of TE regions exhibited that MLs were lower in F_1_ than in the inbred parents, while MLs were higher in F_22_ than in F_1_ and the inbred parents (Fig. 2E).

### Balanced gene expression reduces regulatory system disorders

We performed gene expression analyses in the four embryonic development stages, liver, and barbel tissues of the allotetraploid lineage (Additional file 1: Fig. S23 and Tables S32-S33). The effects of the maternal-to-zygotic transition in F_1_ induced two different trends in the number of expressed genes: first, a decrease in the number of expressed genes from the blastula to gastrula periods revealed that the number of eliminated maternal transcripts was greater than the number of zygotic transcripts; second, an increase in the number of expressed genes from the gastrula to hatching periods showed that the number of zygotic transcripts was greater than the number of eliminated maternal transcripts (Additional file 1: Fig. S24A and Table. S34). The same phenomenon was observed when the expression changes of C homoeologs were examined (Additional file 1: Fig. S24B and Table. S35). However, this phenomenon was not observed in F_22_, where the transcripts of the C homoeologs were inherited in the eggs of the parental F_21_ allotetraploid.

Associative analysis of DNA methylation and gene expression showed that higher levels of gene expression and lower MLs were detected in orthologous or homoeologous gene pairs (OGPs or HGPs) among the inbred parents, F_1_, and F_22_, while lower levels of gene expression and higher MLs were found in SSGs (Additional file 1: Figs. S16 and S25). However, in the blastula, gastrula, and segmentation periods, higher gene expression and higher MLs were observed in SSGs of F_1_. Interestingly, we observed that the expression of SSGs was lower in F_22_ than in the two inbred parents, especially in the two examined tissues and during the hatching period, although the gene expression changes did not consist of DNA methylation changes.

**Fig. 5:**
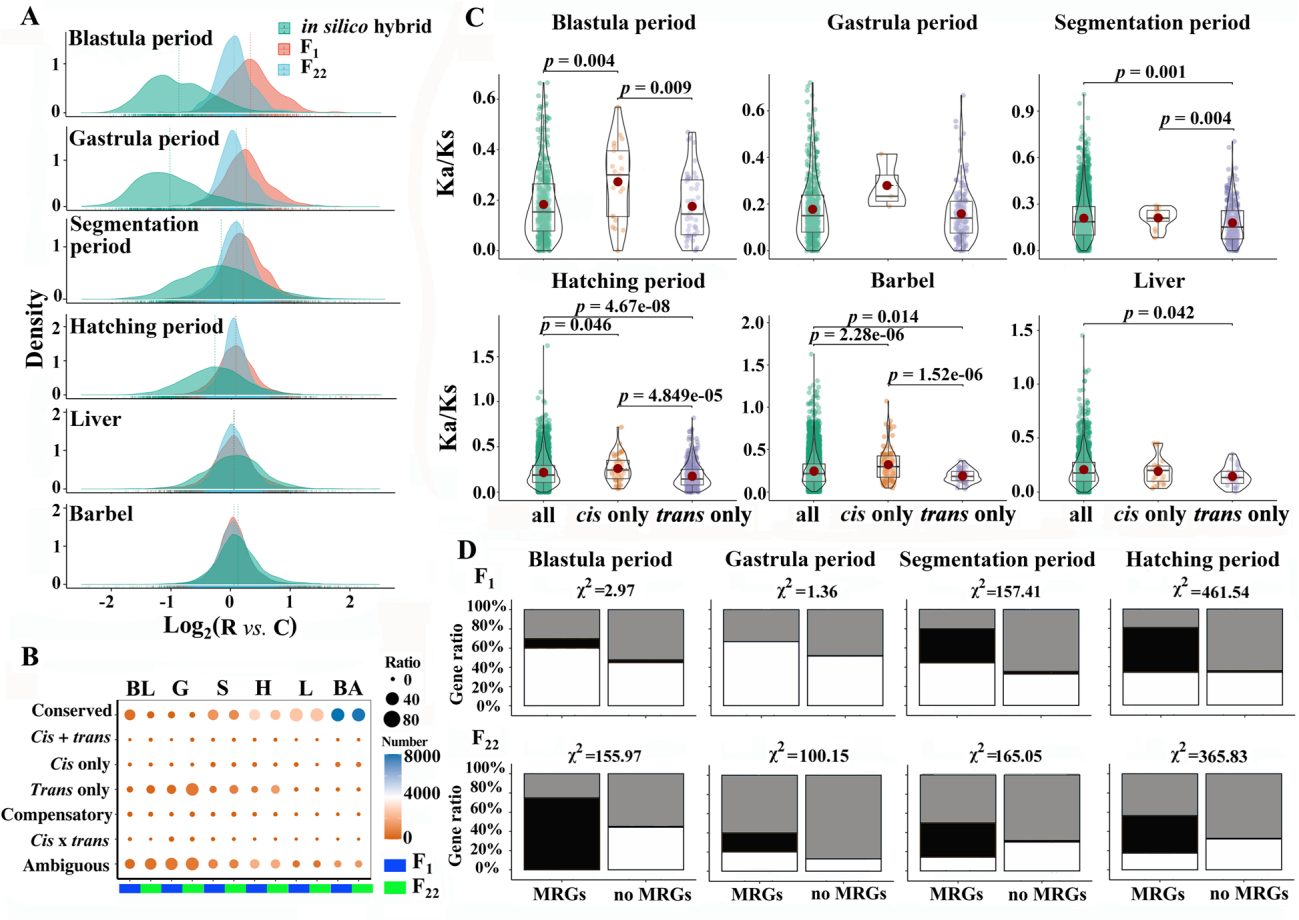
Homoeolog expression changes, *cis*- and *trans*-regulations, and their correlation with K_a_/K_s_. **A** The extent of homoeolog expression bias (HEB) was observed in different developmental stages and tissues. Different gene numbers (705, 762, 2810, 5807, 3039, and 9707 in the blastula, gastrula, segmentation, hatching periods, and liver and barbel tissues, respectively) are assessed in this graph. The density distribution of log_2_ (R*/*C) values was determined in the inbred parents (in silico hybrid), F_1_, and F_22_. Dotted lines represent the average log_2_ (R*/*C) values in each sample. **B** Numbers and percentages of *cis*- and *trans*-regulatory genes in different developmental stages and tissues. **C** Distribution of K_a_/K_s_ values of OGPs in the “*cis*-only”, “*trans*-only”, and total genes (all) of F_1_. The red dots in boxes represent the mean values. **D** Correlation analyses of potential DNA MRGs and “*cis*-only” genes in F_1_ and F_22_. The grey represents “no *cis* only” genes. The black represents “*cis* only” genes. The black represents “ambiguous” genes

Moreover, the expression divergence between R and C homoeologs (expression values of log_2_ ($\frac{\mathrm{R}\ \mathrm{homoeologs}}{\mathrm{C}\ \mathrm{homoeologs}}$) or log_2_(R/C), also described as homoeologous expression bias (HEB)) gradually decreased from the inbred parents (in silico hybrid) to F_22_ (Additional file 1: Fig. S26). The degree of HEB gradually decreased from the blastula to hatching periods accompanying hybridization and polyploidization, reflecting that symmetric homoeolog expression was rapidly established, accompanied by zygotic genome activation (Fig. 5A and Additional file 1: Table. S36). No obvious HEB was observed in the liver and barbel tissues of the “in silico hybrid”. Interestingly, the degree of HEB in F_1_ was lower than that in the in silico hybrid but higher than that in F_22_, in which symmetric expression between the two homoeologs was observed in the four examined periods and the two tissues (Fig. 5A). Overall, the observed expression changes were evenly distributed on each OCP during embryonic development (Additional file 1: Figs. S27-S30).

### Homoeologous expression bias resulting from cis- and trans-regulations

Changes in HEB from inbred parents to hybrids could shed light on the effects of *cis*- and *trans*-regulations on the hybrids’ various genes [14, 33]. “conserved”, “*cis* + *trans*”, and “*cis*-only” genes (the same direction of HEB between the inbred parents and the hybrids) were considered to represent parental effects, while “*trans*-only”, “compensatory”, and “*cis* × *trans*” genes (the different direction of HEB between the inbred parents and the hybrids) may be related to novel effects (Additional file 6: Table S37). The number of “conserved”, “*cis* + *trans*”, and “*cis*-only” genes in the four periods decreased from F_1_ to F_22_, while the number of “*trans*-only” genes increased (Fig. 5B, Additional file 1: Fig. S31, Additional file 6: Table S37). The number of “conserved” genes was greater in the two examined tissues than in the embryos. However, the *cis*- and *trans*-regulatory patterns of the genes observed in F_1_ were largely maintained in F_22_, reflecting that the majority of *cis*- and *trans*-regulatory elements was inherited during polyploidization.

The K_a_/K_s_ ratios were significantly different (*p* < 0.05) between “all” genes and “*trans*-only” genes in the segmentation, hatching periods, and barbel and liver tissues of F_1_ and F_22_ fish (Fig. 5C and Additional file 1: Fig. S32A). The results showed that the *trans*-acting factors originating from the highly conserved orthologous genes of goldfish and common carp preferentially regulate the expression of target R and C homoeologs together. Additionally, significant differences (*p* < 0.05) in K_a_/K_s_ values were observed between “all” genes and “*cis*-only” genes within the same samples (Fig. 5C and Additional file 1: Fig. S32A). This result indicated that *cis*-regulatory effects play dominant roles in the expression of orthologous genes in hybrids, which was highly divergent between goldfish and common carp. No correlation between these values was found in the blastula and gastrula periods, which may also be related to the incomplete zygotic genome activation. Additionally, the correlation coefficients of the K_a_/K_s_ ratios between “all” and “*cis*-only” genes decreased from F_1_ to F_22_, while increased correlation coefficients were detected between “all” and “*trans*-only” genes (Fig. 5C and Additional file 1: Fig. S32).

A negative correlation was detected between the gene expression levels and MLs (2 kb upstream of TSS) of some genes (138–261 in the inbred parents, 10–47 in F_1_, and 4–28 in F_22_), which were considered potential DNA methylation-regulated genes (MRGs) (Fig. 4C and Additional file 1: Fig. S33). Furthermore, a significant correlation was observed between MRGs and the distribution of “*cis*-only” and “*trans*-only” genes, except in the blastula and gastrula periods of F_1_. (Fig. 5D and Additional file 1: Fig. S34). Additionally, MRGs exhibited a higher correlation with “*cis*-only” genes than with “*trans*-only” genes (Additional file 1: Fig. S35). However, the correlation coefficients between MRGs and “*trans*-only” genes in the segmentation and hatching periods were higher in F_22_ than in F_1_. These results indicated that the *trans*-regulation of DNA methylation increased after polyploidization, although the *cis*-regulation of DNA methylation still played a dominant role in hybrids.

## Discussion

Hybridization (especially intergeneric hybridization) results in pre-zygotic and post-zygotic reproductive isolation in vertebrates. This isolation may be related to factors such as chromosome pairing, sex determination, physiological and developmental constraints, and genomic divergence. Polyploidization can restore reproductive fitness, break reproductive isolation, and further give rise to novel species [3, 34]. However, these phenomena are observed more often in plants than in animals. The nascent allotetraploid lineage examined in this work was derived from the hybridization of goldfish and common carp, and subsequent polyploidization (Fig. 1). Studies on genomic, epigenomic, and expression changes in the early stages of allopolyploidization and transgenerational inheritance can help us understand polyploidization in vertebrates.

Polyploidization is always followed by the emergence of unreduced gametes during meiosis, which are likely to emerge in polyploid plants and fishes [35, 36]. Beyond the teleost-specific genome duplication, an additional polyploidization event is observed in the ancestors of goldfish and common carp [15, 16]. Then, rapid genomic divergence occurred in the subsequent evolution of the two species. Empirical observation shows that the divergence between hybrid parents is critical to forming stable allotetraploids [5, 37]. Furthermore, the coexistence of diploid and triploid individuals in the natural *C. auratus* population reflects the goldfish’s high genetic plasticity [38, 39]. Our results exhibited diverse events of unequal HR in different individuals between F_1_ and allotetraploid populations, as well as between somatic and germ cells in the same individual, reflecting the various potential mechanisms of genomic recombination, including DNA double-strand break repair in mitosis [40], and chromosome pairing and exchange during meiosis [41], in them. This previous evidence and our results revealed that high-level genomic plasticity, which may be related to TE increases and other types of changes, may explain the emergence of polyploidy in goldfish, common carp, and their nascent allotetraploid offspring (Fig. 2 and Additional file 1: Figs. S5 and S6).

Polyploidization relies not only on the emergence of unreduced gametes, but also on the survival of allopolyploid individuals. The possibility of survival is always related to the capacity to tolerate excessive developmental abnormalities arising from genome duplication. The destruction of regulatory systems is the main cause of the death of allopolyploid individuals, especially in the early stages of embryonic development [10]. Mismatches between regulatory factors and target genes, and increased gene copy numbers, are always fatal to normal development. The rapid establishment of a suitable regulatory network is important in a nascent allotetraploid. Our results showed that symmetric and balanced expression between R and C homoeologs was mainly established via two steps: hybridization and polyploidization (Fig. 4). The initial balance was established in F_1_, particularly during early embryogenesis. Pre-zygotic reproductive isolation is induced by nuclear-cytoplasmic conflict during embryonic development [42–44]. In the second step, the balance was further modified from the initial state and adapted to the genomic chaos arising from increased gene copy numbers.

In the above two steps, the low MLs of the TE region in F_1_ may facilitate rapidly emerging unequal HR and the appearance of unreduced gametes in the gonads [7, 23], while the high MLs of TEs in F_22_ may reduce the recombination percentages and transcriptional efficiency of duplicate copies of genes, which contributes to the stabilization of cell metabolism after polyploidization (Fig. 2E) [45, 46]. Meanwhile, some rapid DNA methylation changes in central carbon metabolism and neural development may maintain the genetic stability of the nascent allotetraploid fish [47]. The decreased parental effects and increased novel effects from F_1_ to F_22_ reveal that the increased *trans*-regulatory effects may benefit the normal development of allotetraploid embryos adapting to the chaos caused by increased gene copy numbers (Fig. 5B). Meanwhile, the more “conserved” genes in the two examined tissues than in the embryos suggested that conserved HEB is important for the stabilization of organ function. Analysis of K_a_/K_s_ ratios from F_1_ to F_22_ exhibited that the decreased correlation coefficients between “all” and “*cis*-only” genes, and the increased ones between “all” and “*trans*-only” genes, may be related to the exchange of R and C homoeologs, and benefit the establishment of symmetric homoeolog expression (Fig. 5C). Overall, the symmetric homoeologous expression of the nascent allotetraploid depends on diverse strategies, including TE regulation, unequal HR, and *cis*- and *trans*-regulations (e.g. DNA methylation), to stabilize the regulatory system disturbed by genome merger and duplication (Figs. 2, 3, 4, and 5).

## Conclusions

Balanced states in subgenomes and homoeolog expression may be important for decreasing genome incompatibility in the early stages of allopolyploid vertebrates. Concerted genomic and epigenomic changes are highly beneficial to the genetic stabilization of polyploid plants [37]. Our results further reveal that the dynamic genetic changes accompanied by polyploidization play potential roles in stabilizing the regulatory systems in various developmental stages of the nascent allotetraploid fish. This work will help to explain why nascent polyploidization is rarely observed in vertebrates.

## Methods

### Sample determination and whole genome sequencing

The chromosomal locations of 5S rDNA of the intergeneric F_1_, allotetraploid F_22_, and F_24_ and their inbred parents were analysed by fluorescence in situ hybridization. Chromosomal preparations were performed from peripheral kidney cell cultures of each sample based on the standard process [48]. FISH was performed using a probe with a 200-bp 5S rDNA repeat sequence [24].

Genomic DNA was isolated from the muscle tissue of a male allotetraploid of generation 22 (F_22__1) using the DNeasy Blood and Tissue Kit (Qiagen). The quality of DNA was checked on a NanoDrop® ND-1000 spectrophotometer according to the criteria of a 260/280 ratio of ~1.8 and an OD 260/230 ratio of ~2.0. All complete libraries were added to a flow cell for SMRT sequencing using Oxford Nanopore Technologies GridION X5. Then, paired-end libraries of the allotetraploid were obtained and sequenced on the Illumina X Ten platform according to the Illumina standard operating procedure. Sequencing adaptors, duplicated read pairs, and low-quality Illumina reads and bases were removed with fastp (v. 0.21.0) [49]. Genomic DNA extracted from venous blood (F_22__1) was used for the construction of BioNano map. Hi-C libraries were created from whole-blood cells of an allotetraploid (F_22__2). Chimeric fragments representing the original cross-linked long-distance physical interactions were then processed into paired-end sequencing libraries [50].

### Genome assembly

After quality filtering, clean reads from Nanopore sequencing were used in genome assembly using Canu software [51]. The clean reads of Illumina data were aligned against the Quiver-polished assemblies using BWA (v. 0.7.17-r1188) with the default parameters [52]. Based on the resulting BAM files, inconsistencies between polished contigs and Illumina reads were identified with SAMtools (v. 1.10) [53] and VCFtools (v. 1.3.1) [54]. Credible homozygous variation with quality > 20, mapping quality > 40, and a sum of high-quality alt-forward and alt-reverse bases > 2 in the Quiver-polished assemblies were replaced with called bases. The IrysView (BioNano Genomics, v2.5.1) software package was used to produce single-molecule maps and de novo assembled maps and translate them into genome maps. Then, clean reads of HI-C were aligned to the assembled allotetraploid genome with BWA. Only the reads of unique mapped pairs whose mapping quality was greater than 20 were retained. Invalid read pairs filtered by HiC-Pro (v. 2.8.1) were used to correct scaffolds and the scaffolds were clustered, ordered, and oriented into chromosomes using LACHESIS [55].

### Gene prediction and annotation

The full-length transcriptome was obtained using PacBio sequencing for gene annotation. The total RNA from seven tissues (liver, muscle, ovary, kidney, eye, spleen, and heart) was obtained and mixed in equal amounts. The RNA was reverse transcribed using the SMARTer PCR cDNA Synthesis Kit, and PCR amplification was performed using KAPA HiFi PCR Kits. Libraries were constructed from these cDNA products using the SMRTbell Template Prep Kit 1.0. A mixture of library templates and enzyme were used in the PacBio Sequel™ system for sequencing. Sequence reads from the SMRT chip were processed through PacBio’s SMRT-Portal analysis suite to generate circular consensus sequences. BUSCO assessments (v. 4.0) were performed on the assembled genome. Three methods (de novo prediction, homology search, and transcript-based assembly) were used to annotate protein-coding genes. The de novo gene models were predicted using Augustus (v. 2.4) and SNAP (released on 2006-07-28). In homologue-based analysis, GeMoMa (v. 1.7) software was performed using a reference gene model from some cyprinid fish. In transcript-based prediction analysis, RNA-sequencing data was mapped to the reference genome using HISAT (v. 2.0.4) [56] and assembled by StringTie (v. 1.2.3) [57]. Gene prediction was based on the transcripts assembled from Illumina data using GeneMarkS-T (v. 5.1), Additionally, PASA (v. 2.0.2) was used to predict genes based on full-length transcripts assembled by Trinity (v. 2.11). The gene models obtained from these different methods were combined using EVM software (v. 1.1.1) and updated by PASA. The final gene models were annotated by searching the GenBank Non-Redundant (NR, 20200921), TrEMBL (202005), Pfam (v. 33.1), SwissProt (202005), Eukaryotic Orthologous Groups (KOG, 20110125), Gene Ontology (GO, 20200615), and Kyoto Encyclopedia of Genes and Genomes (KEGG, 20191220) databases. TEs and SSRs were annotated based on the above analysis pipelines.

### Synteny and comparative genomics

Syntenic gene analyses were performed in four comparisons, including goldfish and zebrafish (group 1), common carp and zebrafish (group 2), the allotetraploid and goldfish (group 3), and the allotetraploid and common carp (group 4). The syntenic blocks of the allotetraploid, common carp, goldfish, and zebrafish were determined by MCScan (https://github.com/tanghaibao/jcvi) with default parameters [58]. Gene synteny and collinearity were displayed in a schematic diagram created with Circos (v 0.69-6) (http://circos.ca) [59]. Circos was also used as a visualization tool for gene interchromosomal translocation events between the inbred parents and the allotetraploid.

### Genomic divergences of parental genomes in hybrids

Genome data of the parents of the five hybrid groups (10 species) were downloaded. The parental species included the two inbred parents from intergeneric hybridization (*C. carpio* and *C. auratus*) [15, 16], parents from intergeneric hybridization (*C. alburnus* and *M. amblycephala*) [25], and parents from interspecific hybridization (*T. rubripes* and *T. flavidus*) [26, 27], (*O. aureus* and *O. niloticus*) [28, 29], and (*X. hellerii* and *X. maculatus*) [30, 31]. Different versions of goldfish (*C. auratus*) and common carp (*C. carpio*) genomes were downloaded.

The determination of chromosome collinearity between the two parents of the hybrid group was performed using the Multiple Collinearity Scan toolkit and BLASTP method (e-value: 1e^−10^). The distribution of K_s_ and K_a_/K_s_ were calculated with yn00 programme via maximum likelihood method. A combination of homology-based and de novo approaches were used. We customized a de novo repeat library of the genome using RepeatModeler2 (v. 2.0.1) [60]. Then full-length long terminal repeat retrotransposons (fl-LTR-RTs) were identified using both LTRharvest (v. 1.5.9) [61] and LTR_FINDER (v. 1.1) [62]. A non-redundant species-specific TE library was constructed by combining the above de novo TE sequence library with the Repbase (v. 19.06), REXdb (v. 3.0), and Dfam (v. 3.2) databases. Simple sequence repeats (SSRs) were annotated by Tandem Repeats Finder (v. 409) [63] and MIcroSAtellite identification tool (v. 2.1) [64].

### Genomic recombination

To investigate structural variations, especially HR, whole-genome sequencing data of five allotetraploid progenies (F_24__1~F_24__5) were generated on the Illumina NovaSeq 6000 platform; Illumina NovaSeq 6000 and DNBSEQ-T7 sequencing were performed on a mixture containing equal amounts of goldfish and common carp DNA; DNBSEQ sequencing was conducted on muscle and ovary tissues of two F_1_ individuals (F_1__1 and F_1__2); and DNBSEQ sequencing was performed on muscle and gonad tissues of three allotetraploids (F_22__3~F_22__5). Some whole-genome resequencing data were obtained using DNA nanoball (DNB) technology, which combines single-stranded circular library construction, generation, and loading of DNBs onto patterned nanoarrays, and combinatorial probe anchor synthesis sequencing. The raw data of Illumina and DNBSEQ were performed quality checking and adapter removal using fastp. Then, the high-quality clean reads were mapped to the combined genome of goldfish and common carp using BWA with default parameters. Structural variations, including HR, were detected using Manta (v. 1.6.0) with default parameters [65]. The above BAM output files of whole-genome resequencing were also obtained to calculate the number of mapped reads in the coding region of each gene using htseq-count (v. 0.12.4) [66] with a threshold of “-m union --nonunique=none”. The ratio of the mapped read number in each base of R homoeolog *vs.* those of C homoeolog (log_10_ ((R_reads number_/R_length_)/(C_reads number_/C_length_))) was used to predict the copy number changes of R *vs.* C homoeologs led by unequal HR.

### DNA methylation

Total DNA from four developmental stages (blastula (oblong), gastrula (50%-epiboly), segmentation (3-somite), and hatching period (1 h after hatching)) was obtained from goldfish, common carp, the intergeneric F_1_, and allotetraploid F_22_ using a QIAamp DNA Mini Kit (Qiagen, Chatsworth, CA, USA). Whole genome bisulfite sequencing libraries were constructed following the standard protocol. High-quality libraries were sequenced on NovaSeq 6000 Sequencing System with paired-end (2 × 150 bp). After filtering low-quality sequences, the clean reads of the two hybrids were mapped to the assembled genome of the allotetraploid, while the high-quality clean reads of goldfish (Genome Warehouse in BIG Data Center BioProject No.: PRJCA001234) [16] and common carp (NCBI accession No.: PRJNA510861) [15] were mapped to the corresponding genomes. The analysis pipeline of Bismark (v. 0.22.3) was used to detect methylated loci [13, 67]. The unique mapped reads were retained in subsequent analyses. A binomial distribution test was performed to identify 5-methylcytosine (5mC) at each cytosine site. The potential methylation sites were checked according to the thresholds of depth > 4X and FDR < 0.05. Average CpG methylation was detected in different genome regions, including 2 kb upstream of TSS, gene body, and 2 kb downstream of TTS with 20 windows for each region. Average CpG MLs in upstream and downstream transposon regions (2 kb) were calculated and plotted using R.

The regions with different CpG MLs were detected using MOABS [68]. The R packages DSS and bsseq were used to call DMRs based on a threshold of e-value < 1e^−5^. The DMRs in 2 kb upstream of TSS were used to detect DMGs. The DMGs of OGPs in the two inbred parents and HGPs in the hybrids were classified into the following two categories: (1) hyper-DMGs conforming to the thresholds of an absolute value of differences in the methylation ratio between goldfish and common carp (|DMGs_A-B_|) > 0.6 and an absolute value of differences in the methylation ratios between the two homoeologs of the hybrids (|DMGs_As-Bs_|) < 0.3, and (2) hypo-DMGs conforming to the threshold of 0 < |DMGs_A-B_| < 0.6 in the inbred parents and |DMGs_As-Bs_| < 0.2 in hybrid.

### Gene expression

Total RNA from the four periods (as in the DNA methylation analysis) was isolated from goldfish, common carp, F_1_, and F_22_ individuals and purified via a TRIzol extraction method. Additionally, total RNA of barbel tissues (including skin at the root of barbel) in the four fishes (only skin tissue was obtained in goldfish) was isolated using the RNeasy Plus Universal Mini kit (Qiagen) according to the manufacturer’s instructions. The purified RNA was quantified using a 2100 Bioanalyzer system (Agilent, Santa Clara, CA, USA). Illumina mRNA-seq libraries of the four samples from each of the four periods were prepared according to a standard high-throughput method. These libraries were sequenced according to the paired-end (2 × 150 bp) setting using the NovaSeq 6000 Sequencing System (Illumina, Sad Diego, CA, USA). The transcriptome data of barbel tissue was obtained using DNA nanoball (DNBSEQ-T7) technology according to corresponding methods. All samples were conducted with three biological replicates. Additionally, the transcriptome data of the goldfish, common carp, and F_1_ and F_22_ liver tissues were downloaded from Short Read Archive of NCBI database [7]. After quality filtering and adapter trimming, all mRNA-seq reads of the two hybrids were mapped to the assembled genome of the allotetraploid using HISAT2 (v. 2.1.0) with default parameters, while the clean reads of goldfish and common carp were mapped to the corresponding genomes. Then, the mapped files were manipulated with SAMtools (v. 1.10) and the unique mapped reads were obtained using htseq-count. The gene expression value of each sample was normalized based on the total mapped reads among all samples. The analyses of gene silencing on total expression level (combined expression values of R and C homoeologs in hybrids) were performed on the OGPs of the two inbred parents and the HGPs of the hybrids. Silent genes were filtered according to showing a silenced state (mapped reads = 0) or an expressed state (mapped reads ≥ 5) in three biological replicates of the different fishes.

### *Cis-* and *trans-*regulatory effects

Analyses of homoeolog expression were performed only among filtered genes with ≥ 5 mapped reads of each homoeolog in all three biological replicates. The distribution of HEB in hybrids was detected based on the log base 2 value of the R homoeolog expression level divided by C homoeolog expression level (expression values of log_2_ ($\frac{\mathrm{R}\ \mathrm{homoeologs}}{\mathrm{C}\ \mathrm{homoeologs}}$) or log_2_ (R*/*C)), while the log base 2 value of the OGP expression level in goldfish divided by that in common carp (log_2_ (R*/*C)) was considered the reference value (*in silico* hybrid) [14]. Then, HEBs were determined with the threshold of |log_2_ (R*/*C)| > 1 in hybrids, while the potential HEBs were classified based on the threshold of 1 > |log_2_ (R*/*C)| > 0 in hybrids. To further investigate the mechanisms regulating expression divergence, seven *cis*- and/or *trans*-regulatory patterns (“*cis* only”, “*trans* only”, “*cis* + *trans*”, “*cis* x *trans*”, “Conserved”, “Compensatory”, and “Ambiguous”) were established based on significant expression differences between the log_2_ (R*/*C) values of parents and hybrids. The detailed classification methods are described in McManus et al*.* [33]. Significant differences were determined with edgeR (fold change < 4 and *p* < 0.01) in R package. Analyses of significant differences were performed via Student’s *t*-test (*p* < 0.01) of log_2_ (R*/*C) values between parents and hybrids. In addition, we further split the seven *cis*- and/or *trans*-regulatory patterns into 13 patterns based on the plus-minus log_2_ (R*/*C) values of parents or hybrids. The following thresholds were used in this analysis: log_2_ (R*/*C) < log_2_ (0.25) or log_2_ (R*/*C) > log_2_ (4) and DM (DMGs_A-B_ or DMGs_As-Bs_ > 0.3). Pearson’s rank correlation coefficients were used to assess the correlations between the distribution of “*cis* only” or “*trans* only” genes and MRGs.

## Supplementary Information

**Additional file 1: Table S1.** The sampling information of the allotetraploid lineage for the genome, transcriptome, and DNA methylation. **Table S2.** The information of Illumina sequencing data in the allotetraploid. **Table S3.** The information of whole genome sequencing in allotetraploid using Nanopore sequencing. **Table S4.** The information of Bionano genomics data. **Table S5.** The information of Hi-C sequencing data in allotetraploid. **Table S6.** The summary of full-length transcriptome. **Table S7.** The statistics of genome assembly after polishing with Illumina data. **Table S8.** The summary of genome correction and assembly based on analysis pipelines combined with Nanopore, Illumina, and BioNano data. **Table S9.** The statistics of valid mapping results of Hi-C data in the allotetraploid. **Table S11.** Summary of whole genome anchored by HI-C data. **Table S12.** The statistics of BUSCO completeness (v. 4.0). **Table S13.** The gene prediction annotated with the three methods, including ab initio, homology-based, and transcriptome. **Table S14.** The gene number annotated with different databases. **Table S15.** The annotation of repeat sequences in assembly genome of the allotetraploid. **Table S16.** The statistics of SSR distribution in the allotetraploid. **Table S17.** The statistics of non-coding RNAs annotation. **Table S18.** Summary of the 11 genome sequences in our studies. **Table S19.** The divergence time between the two inbred parents in the five hybrid groups. **Table S20.** The statistics of genome assembly results in three versions of goldfish (*C. auratus*) and two versions of common carp (*C****.*** *carpio*) in published papers. **Table S21.** The statistics of BUSCO completeness (v. 4.0). **Table S24.** The statistics of simple sequence repeats (SSR) in the two parent genomes of the five hybrid groups. **Table S26.** The information of whole genome re-sequencing using Illumina and DNB sequencing. **Table S27.** The unequal HRs in muscle, gonad, and caudal fin tissues of the diploid F_1_, the allotetraploid F_22_, and F_24_ individuals based on Illumina and DNB data. **Table S28.** Depth of the allotetraploid reads in regions of the CACTA and hAT superfamilies. **Table S29.** The methyl-seq data in embryos of the allotetraploid lineage. **Table S30.** The mapping information in methyl-seq data of the allotetraploid lineage. **Table S31.**The summary of 5-methylcytosine (5mC) of the allotetraploid lineage. **Table S32.** The information of transcriptomic data of goldfish (RCC), common carp (CC), diploid F_1_ (F_1_), and allotetraploid F_22_ (F_22_). **Table S33.** Summary of mapped reads in transcriptome data. **Table S34.** The silencing of total expression level in goldfish, common carp, F_1_, and F_22_. **Table S35.** The silencing of homoeologs R or C detected in diploid F_1_ and allotetraploid F_22_. **Table S36.** The distribution of homoeologous expression bias (HEB) based on log_2_ (R *vs.* C) in diploid F_1_ and the allotetraploid F_22_ of goldfish and common carp. **Fig. S1.** Heatmap of the allotetraploid constructed by distance of the interactions within and among chromosomes according to Hi-C analyses. Chromosomes predicted by Lachesis were cut into bins of an equal length of 200 kb and a heatmap was constructed based on the interaction signals that were revealed by valid mapped read pairs between bins. **Fig. S2.** The lengths of annotated genes in the allotetraploid, *C. carpio*, *C. auratus*, *Culter alburnus*, *Danio rerio,* and *Ctenopharyngodon idella.* (A) Length of coding sequences in each gene was obtained from these species and the allotetraploid F_22_. (B) Length of gene sequences in each gene. (C). Length of intron sequences in each gene. (D) Length of each exon. **Fig. S3.** Genome synteny of the two subgenomes in goldfish and common carp. Block represents the assembled chromosome. The subgenome M in goldfish and subgenome B in common carp (yellow river carp) were derived from a common maternal ancestor (yellow), while subgenome P in goldfish and subgenome A in common carp were derived from a common paternal ancestor (green). The grey line represents the homologous gene pairs (OGPs) between goldfish and common carp, while black line represents the paralogous gene pairs between the two subgenomes. **Fig. S4.** The genome synteny in two parents of the five hybrid groups, including the hybrid group of *C. carpio* (A, 2n = 100) *vs. C. auratus* (B, 2n = 100), the hybrid group of *C. alburnus* (A, 2n = 48) *vs. M. amblycephala* (B, 2n = 48), the hybrid group of *T. rubripes* (A, 2n = 44) *vs. T. flavidus* (B, 2n = 44), the hybrid group of *O. aureus* (A, 2n = 44) *vs. O. niloticus* (B, 2n = 44), the hybrid group of *X. hellerii* (A, 2n = 48) *vs. X. maculatus* (B, 2n = 48). Colored lines indicate the orthologous sites of gene blocks and their colinear relationships between genomes A and B. The numbers in order were based on the collinearity relationships with the zebrafish genome. **Fig. S5.** Analysis of orthologous genes in the two parental genomes of five hybrid lineages. The rate represents the orthologous gene markers with clear origins of the ancestral parents determined by orthologous gene analysis. Chromosome numbers are ordered according to the collinearity relationships with the zebrafish genome. OCP: orthologous chromosome pair. **Fig. S6.** Analysis of K_a_/K_s_ and K_s_ values in the two parental genomes of five hybrid lineages. (A) The distribution of K_a_/K_s_ values among OGPs. (B) The distribution of K_s_ value among OGPs. **Fig. S7.** The density of TEs in the OCPs of *C. alburnus* (A) *vs. M. amblycephalavs* (B). **Fig. S8.** The density of TEs in the OCPs of *T. rubripes* (A) *vs. T. flavidus* (B). **Fig. S9.** The density of TEs in the OCPs of *O. aureus* (A) *vs. O. niloticus* (B). **Fig. S10.** The density of TEs in the OCPs of *X. hellerii* (A) *vs. X. maculatus* (B). **Fig. S11.** The distribution of TE rates in OCPs. The average deviation value reflects the TE differences between the two parents of the five hybrid groups (*C. carpio vs. C. auratus, C. alburnus vs. M. amblycephala, T. rubripes vs. T. flavidus, O. aureus vs. O. niloticus,* and *X. hellerii vs. X. maculatus*). **Fig. S12.** Genomic variation checked in genome assembly. (A) The three gene interchromosomal translocations (GITs) of the allotetraploid were checked using the mapped PacBio reads (red and blue lines represent forward and reverse reads, respectively). (B) Unequal HR in AT_chr39 (3,426,224-6,057,586 bp) HCP. The mapped PacBio reads (red and blue lines represent forward and reverse reads, respectively) in the allotetraploid (F_22__1) confirmed no assembly error in the breakpoints of syntenic region (black arrow). **Fig. S13.** Determination of unequal HR in the diploid F_1_ and allotetraploid F_24_. (A) Ratio of gene copy numbers of R *vs.* C homoeologs in muscle and gonad tissues of the diploid F_1_ and allotetraploids F_22_ and F_24_. The red solid lines represent the *in silico* prediction of the ratio of R *vs.* C homoeologs (Log_10_(1) = 0). The black dashed lines represent the threshold values of R *vs.* C homoeologs (Log_10_(0.5) = -0.30103 and Log_10_(2) = 0.30103. The dot represent the ratios of R *vs.* C homoeologs (Log_10_(x)) obtained from the numbers of mapped reads of R and C homoeologs in HCPs (different colours) for coding regions. “M” and “G” represent muscle and gonad tissues, respectively. (B) Ratios of gene copy numbers of R *vs.* C homoeologs and homoeologous expression bias (HEB) in the caudal fin tissue of five allotetraploid individuals (F_24_). **Fig. S14.** Correlation relationship between the distribution of TEs and structural variations in each TE type (*t-*test). **Fig. S15.** Methylation levels (MLs) of homoeologs R and C of TSS and TTS in the four development stages of goldfish, common carp, F_1_, and F_22_. (A) DNA methylation in 2 kb upstream of TSS. (B) Difference analyses between MLs of homoeologs R and C in 2 kb upstream of TSS. (C) DNA methylation in 2 kb downstream of TTS. (D) Difference analyses between MLs of homoeologs R and C in 2 kb downstream of TTS. (E) Difference analyses between two adjacent embryonic developmental periods. The symbols “BL”, “G”, “S”, and “H” represent periods of blastula, gastrula, segmentation, and hatching, respectively. **Fig. S16.** The distribution of MLs (2k upstream of TSS) in orthologous gene pairs (OGPs) or HGPs, R or C species-specific genes (SSGs), of goldfish, common carp, F_1_, and F_22_, respectively. Dotted line represents the corresponding average values of methylation rate of the R (orange) or C (light blue) SSGs in the two inbred parents (goldfish and common carp). **Fig. S17.** Differentially methylated regions (DMRs) of the four development stages in subgenomes R and C. In subgenome R, dark red represents higher ML in the former, while light red represents higher ML in the latter. In subgenome C, dark blue represents higher ML in the former, while light blue represents higher ML in the latter. **Fig. S18.** The top 20 pathways of the DMGs led by polyploidization and transgenerational inheritance. (A) The DNA methylation changes in these DMGs induced by hybridization and recovered to the state of the inbred parents by polyploidization and transgenerational inheritance. (B) The novel DNA methylation changes in these DMGs induced by polyploidization and transgenerational inheritance in the allotetraploid lineage. **Fig. S19.** The distribution of MLs in the blastula period. The difference values were detected based on the difference in MLs of homoeologs R and C in the inbred parents, F_1_, and F_22_. **Fig. S20.** The distribution of MLs in the gastrula period. The difference values were detected based on the difference in MLs of homoeologs R and C in the inbred parents, F_1_, and F_22_. **Fig. S21.** The distribution of MLs in the segmentation period. The difference values were detected based on the difference in MLs of homoeologs R and C in the inbred parents, F_1_, and F_22_. **Fig. S22.** The distribution of MLs in the hatching period. The difference values were detected based on the difference in MLs of homoeologs R and C in the inbred parents, F_1_, and F_22_. **Fig. S23.** PCA and cluster analyses performed on expression in F_1_, F_22_, and their inbred parents. (A) PCA analyses. (B) Cluster analyses. Analyses were performed using the Euclidean distance between the 72 samples, which included expression values of homoeologs R and C in F_1_, F_22_ and expression values in their inbred parents (goldfish and common carp). The symbols “BL”, “G”, “S”, “H”, “L”, and “BA” represent periods of blastula, gastrula, segmentation, and hatching, liver and barbel tissues, respectively. **Fig. S24.** The distribution of expressed genes and silencing of homoeolog C (CHS) genes in embryonic development of the four fishes. (A) Maternal-to-zygotic transition was observed in embryonic development of goldfish, common carp, and the two hybrids. Meanwhile, a decreasing number of expressed genes from BL to G and an increasing number of expressed genes from G to H were affected by zygotic genome activation and the elimination of maternal transcripts. (B) An obvious difference was observed between the gene numbers of CHS of F_1_ and F_22_ in different embryonic development stages. The symbols “BL”, “G”, “S”, and “H” represent periods of blastula, gastrula, segmentation, and hatching, respectively. **Fig. S25.** The gene expression level of OGPs or HGPs and R or C SSGs in goldfish, common carp, F_1_, and F_22_, respectively. The gene expression levels of OGPs or HGPs were higher than those in SSGs in all comparisons, except the ones in BA, G, and S periods of F_1_, in which the gene expression level of OGs was lower than those in SSGs. **Fig. S26.** Differential expression between R (red) and C (blue) homoeologous genes in F_1_ and F_22_ and orthologous genes in goldfish (red) and common carp (blue), respectively. **Fig. S27.** Homoeolog expression bias (HEB) of blastula period distributed on each chromosome. HEB was detected based on Log_2_ (R *vs.* C) in parents, F_1_, and F_22_. **Fig. S28.** Homoeolog expression bias (HEB) of gastrula period distributed on each chromosome. HEB was detected based on Log_2_ (R *vs.* C) in parents, F_1_, and F_22_. **Fig. S29.** Homoeolog expression bias (HEB) of segmentation period distributed on each chromosome. HEB was detected based on Log_2_ (R *vs.* C) in parents, F_1_, and F_22_. **Fig. S30.** Homoeolog expression bias (HEB) of hatching period distributed on each chromosome. HEB was detected based on Log_2_ (R *vs.* C) in parents, F_1_, and F_22_. **Fig. S31.** *Cis*- and *trans*-regulation genes distributed in different development stages and tissues. **Fig. S32.** The analyses of K_a_/K_s_ values in *cis*- and/or *trans*-regulatory patterns. (A) The distribution of K_a_/K_s_ values of OGPs in “*cis* only”, “*trans* only”, and the total genes (all) of F_22_. A student's *t* test was performed using “ggstatsplot” package. The mean value of the group is signed with a red dot in box. (B) Difference analysis of K_a_/K_s_ values between the genes in four patterns (“*cis* only”, “*trans* only”, “Conserved”, and “Compensatory”) and all genes, respectively. *P*-value is signed and described by heat map. The symbol ‘*’ represents the *p*-value < 0.05 in student's *t* test; symbol ‘**’ represents the 0.001< *p*-value < 0.01; symbol ‘***’ represents the *p*-value < 0.001. The symbol ‘NA’ represents no value. **Fig. S33.** Gene expression regulated by DNA methylation. Correlation analyses between the gene expression ratios of homoeologs R and C (log_2_ (R *vs.* C)) and values of differential methylation (DM). Red dot indicates the negative correlation between values of DE and DM. Black dot indicates the other values of them. The strict thresholds were settled in the analyses with 0.4 in DM and FC = 4 in DE. **Fig. S34.** Correlation analyses of potential DNA methylation-regulated genes (MRGs) and “*tran* only” genes. **Fig. S35.** The distribution of *cis*-regulated genes and potential DNA methylation-regulated genes (MRGs). The “*cis* only” genes were obtained from analysis pipeline of *cis*- and/or *trans*-regulatory patterns, while MRGs were predicted by a negative correlation between DM and DE in hybrids.

**Additional file 2: Table S10.** The final sets of pseudo chromosomes after anchoring by HI-C data.

**Additional file 3: Table S22.** The summary of gene information in the two parental genomes of the five hybrid groups.

**Additional file 4: Table S23.** The orthologous gene pairs in the two parental genomes of the five hybrid groups.

**Additional file 5: Table S25.** The summary of repeat sequences in the two parental genomes of the five hybrid groups.

**Additional file 6: Table S37.** The summary of *cis*- and *trans*-regulatory divergences in the allotetraploid lineage.

## Data Availability

All data generated or analysed during this study are included in this published article, its supplementary information files, and publicly available repositories. In particular, Additional file 1 of this study has been deposited at the figshare repository [69]. The assembled genomes of goldfish and common carp were downloaded from National Genomics Data Center (accession number: PRJCA001234) [70] and NCBI (accession number: PRJNA510861) [71], respectively. The whole genome sequence data of the allotetraploid was submitted to NCBI BioProject database (https://www.ncbi.nlm.nih.gov) (accession number: PRJNA764075) [72]. All raw data of DNA methylation, mRNA-seq, and whole genome re-sequencing were submitted to National Genomics Data Center (https://ngdc.cncb.ac.cn/?lang=en) (accession number: PRJCA003625) [73].

## Abbreviations

Mya: Million years ago
TEs: Transposable elements
Ks: Synonymous substitutions
Ka: Nonsynonymous substitutions
OCP: Orthologous chromosome pair
HR: Homoeologous recombination
HEB: Homoeologous expression bias
DMRs: Differentially methylated regions
DMGs: Differentially methylated genes
TSS: Transcription start site
TTS: Transcription termination site
MLs: Methylation levels
SSGs: Species-specific genes
OGPs: Orthologous gene pairs
MRGs: Methylation-regulated genes
DM: Differential methylation
F: Caudal fin
M: Muscle
G: Gonad
BL: Blastula
G: Gastrula
S: Segmentation
H: Hatching
HCPs: Homoeologous chromosome pairs
