# Supplementary material for: Symmetric subgenomes and balanced homoeolog expression stabilize the establishment of allopolyploidy in cyprinid fish
Source: BMC Biol. 2022 Sep 14;20:200. doi: 10.1186/s12915-022-01401-4 (PMC9472340; doi:10.1186/s12915-022-01401-4)
Supplement: Supplementary file 1 — Additional file 1: Table S1. The sampling information of the allotetraploid lineage for the genome, transcriptome, and DNA methylation. Table S2. The information of Illumina sequencing data in the allotetraploid. Table S3. The information of whole genome sequencing in allotetraploid using Nanopore sequencing. Table S4. The information of Bionano genomics data. Table S5. The information of Hi-C sequencing data in allotetraploid. Table S6. The summary of full-length transcriptome. Table S7. The statistics of genome assembly after polishing with Illumina data. Table S8. The summary of genome correction and assembly based on analysis pipelines combined with Nanopore, Illumina, and BioNano data. Table S9. The statistics of valid mapping results of Hi-C data in the allotetraploid. Table S11. Summary of whole genome anchored by HI-C data. Table S12. The statistics of BUSCO completeness (v. 4.0). Table S13. The gene prediction annotated with the three methods, including ab initio, homology-based, and transcriptome. Table S14. The gene number annotated with different databases. Table S15. The annotation of repeat sequences in assembly genome of the allotetraploid. Table S16. The statistics of SSR distribution in the allotetraploid. Table S17. The statistics of non-coding RNAs annotation. Table S18. Summary of the 11 genome sequences in our studies. Table S19. The divergence time between the two inbred parents in the five hybrid groups. Table S20. The statistics of genome assembly results in three versions of goldfish (C. auratus) and two versions of common carp (C. carpio) in published papers. Table S21. The statistics of BUSCO completeness (v. 4.0). Table S24. The statistics of simple sequence repeats (SSR) in the two parent genomes of the five hybrid groups. Table S26. The information of whole genome re-sequencing using Illumina and DNB sequencing. Table S27. The unequal HRs in muscle, gonad, and caudal fin tissues of the diploid F1, the allotetraploid F22, and F24 individ [file 12915_2022_1401_MOESM1_ESM.pdf]

# Additional File 1

# Supplementary Method, Tables, and Figures for:

## Symmetric subgenomes and balanced homoeolog expression stabilizes the establishment of allopolyploidy in cyprinid fish

Li Ren[1,*], Xin Gao[1,*], Jialin Cui[1,*], Chun Zhang[1,*], He Dai[2,*], Mengxue Luo[1], Shaofang He[3], Qinbo Qin[1], Kaikun Luo[1], Min Tao[1], Jun Xiao[1], Jing Wang[1], Hong Zhang[1], Xueyin Zhang[1], Yi Zhou[1], Jing Wang[2], Xin Zhao[4], Guiming Liu[4], Guoliang Wang[4], Linhe Huo[4], Shi Wang[1], Fangzhou Hu[1], Rurong Zhao[1], Rong Zhou[1], Yude Wang[1], Qinfeng Liu[1], Xiaojing Yan[1], Chang Wu[1], Conghui Yang[1], Chenchen Tang[1], Wei Duan[1] and Shaojun Liu[1,†]

[1]State Key Laboratory of Developmental Biology of Freshwater Fish, College of Life Sciences, Hunan Normal University, Changsha, 410081, China

[2]Biomarker Technologies Corporation, Beijing, 101300, China

[3]Wuhan Carbon Code Biotechnologies Corporation, Wuhan, 430070, China

[4]Beijing Agro-Biotechnology Research Center, Beijing Academy of Agriculture and Forestry Sciences, Beijing, 100097, China

†Corresponding author, Email: lsj@hunnu.edu.cn

*These authors contributed equally to this work.

Supplementary Method ........ 3
1 Genome sequencing of the allotetraploid ........ 3
1.1 Sample determination ........ 3
1.2 Genomic sequencing using Oxford Nanopore Technology ........ 3
1.3 Genomic sequencing using Illumina platform ........ 3
1.4 Optical map (BioNano Genomics Irys) data collection ........ 3
1.5 Chromosome confirmation capture sequencing ........ 4
1.6 Full length transcriptome data ........ 4
2 The assembly and annotation of allotetraploid genome ........ 4
2.1 *De novo* assembly of allotetraploid genome ........ 4
2.2 Chromosomal organization by Hi-C ........ 5
2.3 Gene prediction and annotation ........ 5
2.4 Prediction of transposon element (TE) and tandem repeats (TRs) ........ 6
2.5 Non-coding RNAs annotation ........ 6
3 Genomic divergences of the inbred parents ........ 6
3.1 Accessions of referenced genomes in the five hybrid groups ........ 6
3.2 Genomic differentiation between two inbred parents in the five hybrid groups ........ 6
3.3 Prediction of repeat sequences in the five hybrid groups ........ 7
4 Comparative genomics analyses in the allotetraploid of goldfish and common carp ........ 7
4.1 Collinearity analysis ........ 7
4.2 Detection of structural variations using whole genome re-sequencing ........ 7
4.3 Detection of unequal homoeologous recombination using short length data ........ 8
4.4 Correlation analysis between distribution of TE region and structural variations ........ 8
5 DNA methylations in different development stages and tissues ........ 8
5.1 Whole genome bisulfite sequencing ........ 8
5.2 Mapping of Methyl-seq reads ........ 9
5.3 Methylation level in transposons and differential methylation analysis ........ 9
5.4 DNA methylation changes in homoeologs R and C ........ 9
6 Gene expression profiles in different development stages and tissues ........ 10
6.1 RNA isolation and transcriptome sequencing ........ 10
6.2 Mapping of mRNA-seq reads ........ 10
6.3 Gene silencing of total expression and homoeolog expression level ........ 10
6.4 Homoeolog expression bias ........ 11
6.5 *Cis*- and *trans*-regulatory patterns ........ 11
6.6 Correlation analysis of *cis*- and *trans*-regulations and $K_a/K_s$ ........ 11
6.7 Gene expression diversity regulated by DNA methylation ........ 11
Supplementary Table ........ 12
Supplementary Figure ........ 33

**Supplementary Method**

## 1 Genome sequencing of the allotetraploid

### 1.1 Sample determination

The DNA content of erythrocytes of goldfish, common carp, intergeneric $F_1$, allotetraploid $F_{22}$ and $F_{24}$ (number = 10 in each type) was measured using a flow cytometry (Cell Counter Analyzer, Partec, Germany). About 0.5–1 ml of blood from each fish was collected from the caudal vein using a syringe containing 100–150 units of sodium heparin. The blood samples were treated. The kidney cells of different samples were used for chromosome observation at the metaphase of mitosis. Concanavalin A was injected into the abdominal cavity of the samples one to three times at a dosage of 2-8μg/g. The interval time was 12-24 h. Two to six hours prior to harvest, colchicine (2-4μg/g) was used to arrest the chromosome at the metaphase. All the kidney tissue in the above samples was ground into 0.8% NaCl. The hypotonic treatment was accomplished with 0.075 mol/L KCl for 40-60 min, followed by fixation in 3:1 methanol-acetic acid. The cells were spread on clean slides. Fluorescence in situ hybridization (FISH) is used to assess the chromosomal location of 5S rDNA according to the method described by Masaru and Hideo with minor modifications. Images were captured with CW4000 FISH software (Leica, Germany). Good-quality metaphase spreads were photographed and used for analysis of karyotypes.

### 1.2 Genomic sequencing using Oxford Nanopore Technology

High-quality genomic DNA of the allotetraploid of generation 22 was isolated from the muscle tissue of male allotetraploid (Sample ID: $F_{22}$_1, Table S1) using DNeasy Blood & Tissue Kits (Qiagen). The quality of DNA was checked by NanoDrop® ND-1000 spectrophotometer with a 260/280 ratio and an 260/230 ratio. Then, Blue Pippin was used to collect long length DNA fragments for gel extraction. And the long length DNA fragments were amplified with magnetic beads. 2μg of gDNA was repaired using NEB Blunt/TA Ligase Master Mix (M0367, USA) and subsequently processed using the ONT Template prep kit (SQK-LSK108, UK) according to the manufacturer's instructions. All complete libraries were put into flow cells for SMRT sequencing using Oxford Nanopore Technologies GridION X5.

### 1.3 Genomic sequencing using Illumina platform

Genomic DNA of the allotetraploid (Sample ID: $F_{22}$_1, Table S1) was used to construct a paired-end library (250 bp) according to the Illumina standard operating procedure (Illumina X Ten). Before the beginning of assembly, the sequencing adaptors of raw reads were removed. Then, contaminated reads (bacterial and viral sequences, etc.) were filtered by aligning them to the NCBI-NR database using BWA (v. 0.7.17-r1188) with default parameters. Fastp (v. 0.21.0) was used to remove duplicated read pairs and low-quality Illumina reads based on the following criteria: reads with ≥ 10% unidentified nucleotides (N); reads with ≥ 10 nt aligned to the adaptor, allowing ≤ 10% mismatches; and reads with ≥ 50% of bases having a Phred quality < 5.

### 1.4 Optical map (BioNano Genomics Irys) data collection

Venous blood from the allotetraploid (Sample ID: $F_{22}$_1, Table S1) was collected for genomic DNA extraction. For extracting long genomic DNA molecules from blood samples, peripheral mononuclear cells from the whole blood were isolated after density gradient centrifugation with Ficoll-Paque PLUS medium and BioNano's commercial plug

lysis protocol. The mega-sized DNA molecules were then extracted according to IrysPrep TM Plug Lysis Long DNA Isolation protocol. Qubit 2.0 was used to quantify DNA concentration in a solution. DNA samples that met the following criteria were used for further experiments: 1) the concentration of DNA; 2) the coefficient of variation from top, middle, and bottom of the DNA solution was less than 25 %; and 3) retaining mega base-size DNA as measured by pulsed field gel electrophoresis.

After quality control of mega base-sized DNA molecules, sequence specific labeling of DNA molecules was performed according to IrysPrep Reagent Kit protocol (BioNano Genomics): sequence specificity was provided by Nickase Nt.BspQ1; labeling was carried out by a nick translation process in the presence of a fluorophore labeled nucleotide; labeled nicks were repaired to restore strand integrity; and DNA molecules were stained for backbone visualization. Automated by the Irys system, stained DNA molecules were loaded into BioNano Genomics NanoChannel chips by electrophoresis. Twelve volts was applied to concentrate DNA molecules into the port, 30v was used to unwind DNA molecules within the pillar structures and to move long molecules into NanoChannel with buffers, and 10v was applied to linearize DNA molecules in 45 × 45 nm NanoChannel. The label positions and lengths of DNA molecules were recorded by the on-board CCD camera using green and blue lasers in the BioNano Genomics Irys system.

### 1.5 Chromosome confirmation capture sequencing

Hi-C libraries were created from whole-blood cells of the allotetraploid (Sample ID: $F_{22}$_2, Table S1). Briefly, cells were fixed with formaldehyde and lysed, and the cross-linked DNA was digested with HindIII enzyme. Sticky ends proximity ligated to form chimeric junctions that were enriched for and then physically sheared to a size of 300–700 bp as illustrated in Rao et al. Chimeric fragments representing the original cross-linked long-distance physical interactions were then processed into paired-end sequencing libraries.

### 1.6 Full length transcriptome data

To annotate the assembled genome, we performed PacBio sequencing of mRNA to obtain the long length or full-length transcripts. Thus, transcripts of the allotetraploid were obtained with PacBio SMRT sequencing. The total RNA from seven tissues (liver, muscle, ovary, kidney, eye, spleen, and heart) was obtained and mixed in equal amounts. The RNA was reversely transcribed using the SMARTer PCR cDNA Synthesis Kit, and PCR amplification was performed using KAPA HiFi PCR Kits. The PCR product (size = 0.5-6 Kb and > 6 Kb) was selected based on the agarose gel electrophoresis method. Then, libraries were constructed from these cDNA products using the SMRTbell Template Prep Kit 1.0. After library preparation, the library template and enzyme mixture were used in the PacBio Sequel™ system for sequencing. Then, the low-quality data (adapter sequences, length of subreads < 50 bp, accuracy rate < 0.75) was deleted from the raw data. Sequence reads from the SMRT chip were processed through PacBio's SMRT-Portal analysis suite to generate circular consensus sequences (CCSs). To obtain more accurate reads, the reads (number of cycles of CCS > 1 and accuracy > 0.8) were used to obtain full-length reads (lengths > 300 bp, poly (A) tails, 5' primers and 3' primers) based on the software of SMRT Analysis (v. 2.1).

## 2 The assembly and annotation of allotetraploid genome

### 2.1 *De novo* assembly of allotetraploid genome

The adapter, low quality bases and short reads (<500bp) of Nanopore sequencing data were filtered before assembly. All clean reads were used in genome assembly using Canu software. After removing adapters and low-quality bases, clean reads of Illumina data in the allotetraploid were aligned against the Quiver-polished assemblies using BWA with default parameters. Based on resulting BAM files, inconsistencies between polished contigs and Illumina reads were identified with samtools (v. 1.10) and VCFtools (v. 1.3.1). Credible homozygous variation with quality > 20, mapping quality > 40, and the sum of high-quality alt-forward and alt-reverse bases > 2 in the Quiver-polished assemblies were replaced with the called bases. The IrysView (BioNano Genomics, v2.5.1) software package (https://bionanogenomics.com/support/software-downloads/) was used to produce single-molecule maps and *de novo* assembled maps into a genome map. As for the genome size of the allotetraploid, the default parameters of optArguments_human.xml were chosen following the protocol recommendation. After anchoring the sequences onto the genome map, the analysis of molecular quality report was performed based on map reads in the allotetraploid.

## 2.2 Chromosomal organization by Hi-C

The clean reads of Hi-C were obtained from the trimming of adapter sequences and low quality pair-end reads in the raw data. Then, the only clean Hi-C reads, accounting for 69 fold coverage of the allotetraploid genome, were first truncated at the putative Hi-C junctions and then the resulting trimmed reads were aligned to the assembly results with BWA. Only unique mapped reads with a mapping quality of more than 20 remained for further analysis. Invalid read pairs, including Dangling-End and Self-cycle, Re-ligation, and Dumped products, were filtered by HiC-Pro (v. 2.8.1). Any two segments which showed inconsistent connection with information from the raw scaffolds were checked manually. These corrected scaffolds were then assembled with LACHESIS. The parameters for running LACHESIS were listed as follows: CLUSTER_MIN_RE_SITES = 534, CLUSTER_MAX_LINK_DENSITY = 2, ORDER_MIN_N_RES_IN_TRUNK = 798, ORDER_MIN_N_RES_IN_SHREDS = 966 in the allotetraploid. After this step, placement and orientation errors exhibited obvious discrete chromatin interaction patterns were manually adjusted.

## 2.3 Gene prediction and annotation

The three methods, including *de novo* prediction, homology search, and transcript-based assembly, were used to annotate protein-coding genes. The *de novo* gene models were predicted using Augustus (v. 2.4) and SNAP (release on 2006-07-28). In homolog-based analysis, GeMoMa (v. 1.7) software was performed by using reference gene models from the five species (*C. carpio*, *Ctenopharyngodon idella*, *Danio rerio*, *C. auratus* and *Culter alburnus*). In transcript-based prediction analysis, RNA-sequencing data (Illumina platform) of some embryos and tissues, including liver, testis, muscle, ovary, kidney, eye, spleen, and heart, were mapped to the reference genome using Hisat (v. 2.0.4) and assembled by Stringtie (v. 1.2.3). While gene prediction was based on the assembled transcripts using GeneMarkS-T (v. 5.1), PASA (v. 2.0.2) was used to predict genes based on the unigenes (and full-length transcripts from the PacBio sequencing), which were assembled by Trinity (v. 2.11). Gene models from these different methods were combined using the EVM software (v. 1.1.1) and updated by PASA. After the further filtering was performed with the threshold of sequence similarity > 50% and coverage rate > 50% with homology and transcriptome, 86,180 genes were retained in our next analysis. The final gene models were annotated by searching the GenBank

Non-Redundant (NR, 20200921), TrEMBL (202005), Pfam (v. 33.1), SwissProt (202005), eukaryotic orthologous groups (KOG, 20110125), gene ontology (GO, 20200615), and Kyoto Encyclopedia of Genes and Genomes (KEGG, 20191220) databases.

**2.4 Prediction of transposon element (TE) and tandem repeats (TRs)**

Transposon element (TE) is annotated by the following analysis pipeline: Firstly, TE was identified by a combination of homology-based and *de novo* approaches. We customized a *de novo* repeat library of the genome using RepeatModeler2 (v. 2.0.1), which can automatically execute two *de novo* repeat finding programs, including RECON (v. 1.08) and RepeatScout (v. 1.0.6). Then full-length long terminal repeat retrotransposons (fl-LTR-RTs) were identified using both LTRharvest (v. 1.5.9) (-minlenltr 100 -maxlenltr 40000 -mintsd 4 -maxtsd 6 -motif TGCA -motifmis 1 -similar 85 -vic 10 -seed 20 -seqids yes) and LTR_FINDER (v. 1.1) (-D 40000 -d 100 -L 9000 -l 50 -p 20 -C -M 0.9). The high-quality intact fl-LTR-RTs and non-redundant LTR library were then produced by LTR_retriever. A non-redundant species-specific TE library was constructed by combining the *de novo* TE sequences library above with the known Repbase (v. 19.06), REXdb (v. 3.0), and Dfam (v. 3.2) databases. Finally, TEs were identified and classified by homology search against the library using RepeatMasker, while TRs were annotated by Tandem Repeats Finder (v. 409) and MIcroSAtellite identification tool (v. 2.1).

**2.5 Non-coding RNAs annotation**

Non-coding RNAs, including miRNA, rRNA, tRNA, snoRNA, and snRNA were predicted based on different databases as follows: tRNA was predicted using tRNAscan-SE (v. 1.3.1) with eukaryote parameters. Identification of the rRNA genes was conducted by barrnap (v. 0.9). miRNAs were identified by searching miRBase databases (release 21). The snoRNA and snRNA genes were predicted using INFERNAL against the Rfam (release 12.0) database.

**3 Genomic divergences of the inbred parents**

**3.1 Accessions of referenced genomes in the five hybrid groups**

In total, genome sequences and annotation files of 10 species, which were the inbred parents of five hybrid groups, were downloaded in our analysis, including an allotetraploid complex originating from intergeneric hybridization (*Cyprinus carpio* and *Carassius auratus*, 2n = 100, group 1), diploid complex origin from intergeneric hybridization (*Culter alburnus* and *Megalobrama amblycephala*, 2n = 48, group 2), diploid complex origin from the interspecific hybridization (*Takifugu rubripes* and *T. flavidus*, 2n = 44, group 3), diploid complex origin from the interspecific hybridization (*Oreochromis aureus* and *O. niloticus*, 2n = 44) (group 4), diploid complex origin from the interspecific hybridization (*Xiphophorus hellerii* and *X. maculatus*, 2n = 48) (group 5). Among these genomes, the three genome versions of goldfish (*C. auratus*) and two versions of common carp (*C. carpio*) were found in published database. According to population classification of the two inbred parents of the allotetraploid, version 1 of *C. auratus* and *C. carpio* (> 90% complete BUSCOs) was obtained in the next analyses (Tables. S20 and S21).

**3.2 Genomic differentiation between two inbred parents in the five hybrid groups**

To investigate hybridization incompatibilities between goldfish and common carp, we focused on the divergence between the two paternal genomes. Some features of the genomes and genes were obtained in the five hybrid groups

based on their annotation files. The lowest rate of orthologous gene number between the two parents (39.83% in common carp and 41.19% in goldfish) reflected their high genome diversity. The determination of chromosome collinearity between the two inbred parents was performed using the Multiple Collinearity Scan toolkit (MCScanX) and BLASTP method (e-value: $1e^{-10}$).

### 3.3 Prediction of repeat sequences in the five hybrid groups

A combination of homology-based and *de novo* approaches were used in the annotation of repeat sequences. We customized a *de novo* repeat library of the genome using RepeatModeler2 (v. 2.0.1), which can automatically execute two *de novo* repeat finding programs, including RECON (v. 1.08) and RepeatScout (v. 1.0.6). Then full-length long terminal repeat retrotransposons (fl-LTR-RTs) were identified using both LTRharvest (v. 1.5.9) (-minlenltr 100 -maxlenltr 40000 -mintsd 4 -maxtsd 6 -motif TGCA -motifmis 1 -similar 85 -vic 10 -seed 20 -seqids yes) and LTR_FINDER (v. 1.1) (-D 40000 -d 100 -L 9000 -l 50 -p 20 -C -M 0.9). The high-quality intact fl-LTR-RTs and non-redundant LTR library were then produced by LTR_retriever. A non-redundant species-specific TE library was constructed by combining the *de novo* TE sequences library above with the known Repbase (v. 19.06), REXdb (v. 3.0), and Dfam (v. 3.2) databases. Finally, simple sequence repeats (SSRs) were annotated by Tandem Repeats Finder (v. 409) and MIcroSAtellite identification tool (v. 2.1). The different categories of repeat sequences were identified and classified by homology search against the library using RepeatMasker (v. 4.0.5).

## 4 Comparative genomics analyses in the allotetraploid of goldfish and common carp

### 4.1 Collinearity analysis

The syntenic gene analyses were performed among the allotetraploid, common carp, goldfish, and zebrafish. The proteins of the allotetraploid, common carp, goldfish, and zebrafish were used to perform all–against–all reciprocal BLASTP (v 2.2.26) comparisons with default parameters. The alignments were used with MCScan (Python version) (https://github.com/tanghaibao/jcvi) to determine syntenic blocks with default parameters. The 29,028 gene pairs were used to determine the subgenomes R (origin from goldfish) and C (origin from common carp) in the allotetraploid, while 29,781 gene pairs between goldfish and common carp were used to assess the 50 OCPs. Finally, 13,244 homologous gene pairs were obtained in both the two inbred parents and the allotetraploid progenies and used in the next analyses. Gene synteny and collinearity were displayed as a schematic diagram created with Circos (v 0.69-6) (http://circos.ca). Conserved genome synteny in the two subgenomes (n=25) of goldfish and common carp exhibited the recent allopolyploidization in both of them, which was led by the hybridization and ployploidization in 13.75 Mya. Circos was also used as a visualization tool for gene interchromosomal translocation (GIT) events between parents and hybrid progeny. Syntenic gene analyses in each homoeologous chromosome pairs (HCPs) of the allotetraploid and corresponding OCPs of the two inbred parents could use to detect the potential unequal homoeologous recombination (HR) based on two types of results of synteny block.

### 4.2 Detection of structural variations using whole genome re-sequencing

To investigate structural variations, especially HR, whole-genome data was obtained from sequencing of Illumina NovaSeq 6000 in the five allotetraploid individuals with generation 24 ($F_{24}$_1~$F_{24}$_5), Illumina NovaSeq 6000 and DNA nanoball (DNBSEQ-T7) in the equally mixed DNA of goldfish and common carp, DNBSEQ-T7 in muscle and

ovary tissues of $F_1$_1 and 2, respectively, and DNBSEQ-T7 in muscle and gonad tissues of the three allotetraploid individuals with generation 22 ($F_{22}$_3~$F_{22}$_5). The Illumina data of the two inbred parents (goldfish and common carp), which was obtained from NCBI database (accession nos.: SRR2061049.1-SRR2061053.1, SRR2060961.1, and SRR924680.1) (Table S1).

Parts of whole genome re-sequencing data were obtained using DNBSEQ-T7 technology, which combined single-stranded circular library construction, generation, and loading of DNBs onto patterned nanoarrays, and combinatorial probe anchor synthesis sequencing. The raw data of Illumina and DNBSEQ-T7 were performed quality checking and adapter removing using fastp. Then, the high-quality reads were mapped to the combined genome of goldfish and common carp using BWA with the default parameters. Meanwhile, structural variations were detected using Manta (v. 1.6.0) with default parameters.

### 4.3 Detection of unequal homoeologous recombination using short length data

Short mapped reads of Illumina and DNB sequencing could be used to assess unequal HR in somatic and germ cells of intergeneric $F_1$ and allotetraploid $F_{22}$ population. Coordinate-sorted BAM output files of whole genome re-sequencing were obtained to calculate the number of mapped reads in coding region of each homoeologous gene pair (HGP) using htseq-count (v. 0.12.4) with threshold of "-m union --nonunique=none". The ratio of mapped read number in each base of homoeolog R *vs.* ones in homoeolog C ($\log_{10}$ (($R_{\text{reads number}}/R_{\text{length}}$)/($C_{\text{reads number}}/C_{\text{length}}$)) could use to assess the changes of copy number of homoeologs R *vs.* C led by unequal HR. For obtaining the accurate results, the equally mixing DNA of goldfish and common carp was sequenced using Illumina and DNB platform. Then, the values of $\log_{10}$ (($R_{\text{reads number}}/R_{\text{length}}$)/($C_{\text{reads number}}/C_{\text{length}}$)) of them were used as calibration for corresponding data in different sequencing platforms. Whole-genome and transcriptome data of caudal fin tissue were obtained from the five allotetraploid progenies ($F_{24}$_1~$F_{22}$_5) using Illumina NovaSeq 6000 system, providing us a way to investigate unequal HR contributing to homoeologous expression bias (HEB). Unfortunately, these analyses were not performed in other tissues, while potential phenotypes shaped by their effects were not observed in the five individuals.

### 4.4 Correlation analysis between distribution of TE region and structural variations

A correlation analysis was performed between the distribution of the TE region and structural variations. The TE region was defined as a 1 kb region near the TE, while the no-TE region was the other region in the genome. Furthermore, we performed analyses of the distribution of the structural variations and TE in each TE type. The number of structural variations in each TE region was calculated with the average structural variations in the population of allotetraploid individuals based on whole genome re-sequencing.

## 5 DNA methylations in different development stages and tissues

### 5.1 Whole genome bisulfite sequencing

Total DNA of the four development stages (19-22℃), including blastula (Oblong), gastrula (50%-Epiboly), segmentation (3-Somite) and hatching period (1h after hatch) of the allotetraploid lineage (goldfish, common carp, intergeneric $F_1$ and allotetraploid $F_{22}$) were extracted using a QIAamp DNA Mini Kit (Qiagen, Chatsworth, CA, USA), and then was fragmented with a Covaris S2 Ultrasonicator. Whole Genome Bisulfite Sequencing (WGBS) libraries were constructed following the standard protocol. Briefly, genomic DNA with a 0.5% unmethylated lambda DNA

(Fermentas) spike-in was first sonicated into 200–300 bp fragments using Covaris S220 system. Bisulfite conversion was conducted with EZ DNA Methylation™ Kit (Zymo Research), after bisulfite conversion, the converted templates were PCR amplified and quantified with Qubit2.0. Then, the length of insert fragment in libraries was detected by Agilent 2100, and the effective concentration (>2 nM) was quantified by a qPCR assay. The final quality-insured libraries were sequenced on NovaSeq 6000 Sequencing System with paired-end (2 × 150 bp).

**5.2 Mapping of Methyl-seq reads**

After quality check of the Methyl-seq reads, the clean reads of the two hybrids were mapped to the assembly genome of the allotetraploid, while the high-quality clean reads of goldfish were mapped to their genomes (goldfish: Genome Warehouse in BIG Data Center BioProject No.: PRJCA001234) (common carp: NCBI accession No.: PRJNA510861), respectively. The analysis pipeline of Bismark (v. 0.22.3) was used to detect the methylated loci with mapped parameters (--score-min L,0,-0.2 --ignore-quals --no-mixed --no-discordant). Total million reads mapped to the unique sites were used for the next analyses. The clean reads were mapped to the reference genome four times, and only the reads mapped to the same position of the reference genome were retained in the next analyses. A binomial distribution test was performed to identify 5-methylcytosine (5mC) for each cytosine site. Then, the potential methylation sites were checked with the threshold of coverage > 4X and false discovery rate < 0.05.

**5.3 Methylation level in transposons and differential methylation analysis**

Average CpG methylation was detected in different genome regions, including 2 kb upstream of the transcription start site (TSS), gene body, and 2 kb downstream of the transcription termination sites (TTS) with 20 windows for each region. A series of CpG methylation level changes (2 kb upstream of TSS) were observed in the 50 HGP based on the ratio of MLs of homoeologs R *vs.* C in each HGP. Transposons of goldfish, common carp, and $F_{22}$ were predicted using RepeatMasker, and the average CpG methylation in upstream and downstream transposon regions (2 kb) was calculated and plotted using R. The regions with different CpG methylation were detected using MOABS. The R packages of DSS and bsseq were used to detect differentially methylated regions (DMRs) based on a threshold of e-value < $1e^{-5}$.

**5.4 DNA methylation changes in homoeologs R and C**

The DMRs in 2 kb upstream of TSS were used to detect differentially methylated genes (DMGs). The analyses on DMGs were performed in the comparison of HGPs of R and C in the hybrids ($F_1$ and $F_{22}$), comparison of OGPs between goldfish and common carp, the comparison of R or C among parents and the hybrids. The DMGs of OGPs in the two inbred parents and HGPs in the hybrids were classified into the following two categories: 1) hyper-DMGs conforming to the thresholds of an absolute value of differences in the methylation ratio between goldfish and common carp ($|DMGs_{A\text{-}B}|$) > 0.6 and an absolute value of differences in the methylation ratios between the two homoeologs of the hybrids ($|DMGs_{As\text{-}Bs}|$) < 0.3; and 2) hypo-DMGs conforming to the threshold of $0 < |DMGs_{A\text{-}B}| < 0.6$ in the inbred parents and $|DMGs_{As\text{-}Bs}| < 0.2$ in hybrid.

## 6 Gene expression profiles in different development stages and tissues

### 6.1 RNA isolation and transcriptome sequencing

To obtain gene expression profiles of goldfish, common carp, $F_1$ and $F_{22}$, total RNA of the samples from the periods of BL, G, S, and H (the samples as described in the method of DNA methylation) was isolated and purified by the TRIzol extraction method. Meanwhile, total RNA of barbel tissue (including skin at the root of the barbel) of the four fishes (only the skin tissue obtained in goldfish) was isolated using the RNeasy Plus Universal Mini kit (Qiagen) according to the manufacturer's instructions and included an enzymatic DNase (Qiagen) digestion step. Then, all the RNA concentrations were measured using NanoDrop technology. Total RNA samples were treated with DNase I (Invitrogen) to remove any contaminating genomic DNA. The purified RNA was quantified using a 2100 Bioanalyzer system (Agilent, Santa Clara, CA, USA). 1 μg of isolated mRNA was fragmented with fragmentation buffer. The resulting short fragments were reverse transcribed and amplified to produce cDNA. Illumina mRNA-seq libraries of the four samples from the BL, G, S, and H periods were prepared according to the standard high-throughput method. The quality of the cDNA library was assessed by the Agilent Bioanalyzer 2100 system. Then, the library was sequenced with a paired-end (2 × 150 bp) setting using the NovaSeq 6000 Sequencing System (Illumina, Sad Diego, CA, USA). The transcriptome data of barbel tissue was obtained using DNA nanoball (DNBSEQ-T7) technology according to the standard method. All samples were conducted with three biological replicates. Meanwhile, the transcriptome data of liver (two years old goldfish, common carp, $F_1$, and $F_{22}$) were downloaded from Short Read Archive (SRA) of NCBI database (accession numbers SRX668436, SRX175397, SRX668453, SRX177691, SRX671568, SRX671569, SRX668467, and SRX1610992). Then, low-quality bases and adapters were trimmed out using fastp. The high-quality reads were used in the next analysis.

### 6.2 Mapping of mRNA-seq reads

All the mRNA-seq reads of the hybrids were mapped to the assembly genome of the allotetraploid using HISAT2 (v. 2.1.0) with default parameters, while the high-quality clean reads of goldfish and common carp were mapped to their genomes, respectively. Then, the mapped files were handled with samtools (v. 1.10) and the unique mapped reads were obtained using htseq-count. The expression value was normalized based on the ratio of the number of mapped reads for each gene to the total number of mapped reads for the entire genome. These unique mapped reads satisfied with species-specific SNPs and loci in corresponding subgenomes would be used to calculate expression values of homoeologs R and C in $F_1$ and $F_{22}$.

### 6.3 Gene silencing of total expression and homoeolog expression level

The analyses of gene silencing on total expression level (combined expression values of homoeologs R and C in hybrids) were performed on OGPs of the inbred parents and HGPs of the hybrids. The silent genes were fileted with the silencing state (mapped reads = 0) or the expression state (mapped reads ≥ 5) in three biological replicates of the different fishes. In addition, the silencing of homoeolog R or C was detected based on a threshold of mapped read counts ≥ 5 in three biological replicates of goldfish and common carp, with no mapped reads in homoeolog R or C in three biological replicates of each hybrid. In comparison of Silencing of homoeolog C (CHS) between the adjacent developmental stages of $F_1$, the novel CHS (specific in latter stage) reflected the specific expression of homoeolog C in specific development stage. The CHS shared between the contiguous development stages showed no transcriptional

activation of homoeolog C in them.

### 6.4 Homoeolog expression bias

To avoid the negative effect of expression noise, the analyses on homoeolog expression were performed on only filtered genes with a number of mapped reads ≥ 5 in each homoeolog of all three biological replicates. The distribution of homoeolog expression bias (HEB) in the hybrids was detected based on log base 2 ($log_2$) of expression level of homoeolog (HEL) R divided by C HEL ($log_2$ (R *vs.* C)), while log base 2 of expression level of goldfish divided by common carp ($log_2$ R *vs.* C) in OGPs in the inbred parents was considered as the reference values (*in silico* hybrid). Then, HEBs were determined with the threshold of $|log_2$ (R *vs.* C)$| > 1$ in hybrids, while the potential HEBs were classified based on the threshold of $1 > |log_2$ (R *vs.* C)$|) > 0$ in hybrids.

### 6.5 *Cis*- and *trans*-regulatory patterns

To further investigate the potential mechanisms of expression divergence that raise from changes in homoeologs R and C, seven *cis*- and/or *trans*-regulatory patterns ("*cis* only", "*trans* only", "*cis* + *trans*", "*cis* x *trans*", "Conserved", "Compensatory", and "Ambiguous") were established based on significant expression differences between values of $log_2$ (R *vs.* C) in the inbred parents and hybrids, the detailed classification methods as described in McManus *et al.*. Significant differences were performed with edgeR (fold change < 4 and $p < 0.01$) in R package. The significant differences analyses were performed with a student's $t$ test ($p < 0.01$) of $log_2$ (R *vs.* C) values between parents and hybrids. In addition, we further classified the seven *cis*- and/or *trans*-regulatory patterns into 13 patterns based on the plus-minus of $log_2$ (R *vs.* C) values in parents or hybrids.

### 6.6 Correlation analysis of *cis*- and *trans*-regulations and $K_a/K_s$

To investigate the potential regulatory mechanisms of *cis*- and *trans*-regulatory expression, correlation analyses were performed in $K_a/K_s$ values between each other of the four *cis*- and/or *trans*-regulatory patterns ("*cis* only", "*trans* only", "Conserved", and "Compensatory") and the all genes using Student's $t$ test in 'ggstatsplot' of R package. Further correlation analyses were performed between $K_a/K_s$ and the absolute value of the difference between $log_2$ (R *vs.* C) in the inbred parents and the two hybrids using Pearson's rank correlation coefficients in the 'ggstatsplot' of the R package.

### 6.7 Gene expression diversity regulated by DNA methylation

To investigate how DNA methylation in promoter region regulated homoeolog expression levels in different development stages and tissues, correlation analyses were performed between $log_2$ (R *vs.* C) and $DMGs_{A-B}$ in the inbred parents or $DMGs_{As-Bs}$ in the hybrids. The thresholds for differential expression (DE) ($log_2$ (R *vs.* C) $< log_2$ (0.25) and $log_2$ (R *vs.* C) $> log_2$ (4)) and differential methylation (DM) ($DMGs_{A-B}$ or $DMGs_{As-Bs} > 0.3$) were used in this analysis. To investigate whether there is a correlation relationship between DNA methylation divergence and *cis*- or *trans*- regulatory expression, which was predicted by the expression divergence of homoeologs, Pearson's rank correlation coefficients were used to assess the correlation between the distribution of "*cis* only" or "*trans* only" genes and DNA methylation regulated genes (MRGs).

**Supplementary Table**

Table S1. The sampling information of the allotetraploid lineage for the genome, transcriptome, and DNA methylation.

| Sampling ID | Individual feature | Gender | Sampling tissue | DNA/mRNA | Sequencing technology |
|---|---|---|---|---|---|
| $F_{22}$_1 | Allotetraploid $F_{22}$ | Male | Muscle | DNA | Nanopore sequencing for genome assembling |
| $F_{22}$_1 | Allotetraploid $F_{22}$ | Male | Muscle | DNA | NGS sequencing on Illumina Hiseq X Ten platform for correction and polishing |
| $F_{22}$_1 | Allotetraploid $F_{22}$ | Male | Venous blood | DNA | Optical map by BioNano platform |
| $F_{22}$_2 | Allotetraploid $F_{22}$ | Male | Venous blood | DNA | Chromosome confirmation capture (Hi-C) sequencing on Illumina Hiseq X Ten platform |
| $F_{24}$_1 ~ $F_{24}$_5 | Allotetraploid $F_{22}$ | Male | Caudal fin | DNA | NGS sequencing on Illumina NovaSeq 6000 platform for recombination analysis |
| *In silico* hybrid | *C. auratus* + *C. carpio* | / | Muscle | Equally mixing DNA | NGS sequencing on Illumina NovaSeq 6000 and DNBSEQ-T7 platform for correcting in recombination analysis |
| $F_1$_1 and 2 | Intergeneric $F_1$ | Female | Muscle + ovary | DNA | NGS sequencing on DNBSEQ-T7 platform for recombination analysis |
| $F_{22}$_3~$F_{22}$_5 | Allotetraploid $F_{22}$ | Male and female | Muscle + gonad | DNA | |
| RCC | *C. auratus* | | Embryo | DNA + mRNA | WGBS for detecting DNA methylation, transcriptome sequencing on Illumina NovaSeq 6000 platform |
| CC | *C. carpio* | | Embryo | DNA + mRNA | WGBS for detecting DNA methylation, transcriptome sequencing on Illumina NovaSeq 6000 platform |
| $F_1$ | Intergeneric $F_1$ | | Embryo | DNA + mRNA | WGBS for detecting DNA methylation, transcriptome sequencing on Illumina NovaSeq 6000 platform |
| $F_{22}$ | Allotetraploid $F_{22}$ | | Embryo | DNA + mRNA | WGBS for detecting DNA methylation, transcriptome sequencing on Illumina NovaSeq 6000 platform |

Note: Whole Genome Bisulfite Sequencing (WGBS).

Table S2. The information of Illumina sequencing data in the allotetraploid.

| Sample | Library Type | Insert Size (bp) | Read number | Base number (bp) |
|---|---|---|---|---|
| Tetraploid | paired-end | 250 | 1048.38 million | 164.9 Gb |

Table S3. The information of whole genome sequencing in allotetraploid using Nanopore sequencing.

| Sample | Total raw data (Gb) | Total clean data (Gb) | No. of clean reads | Average length of clean reads (Kb) | N50 of clean reads (Kb) | Depth |
|---|---|---|---|---|---|---|
| Tetraploid | 126.8 | 119.0 | 7,226,637 | 16.4 | 23.3 | 43 x |

Table S4. The information of Bionano genomics data.

| Sample | Clean data (Gb) | Average label/100 Kb of clean data | N50 of clean data (Kb) | Depth |
|---|---|---|---|---|
| Tetraploid | 552.45 | 11.18 | 219.4 | 184 x |

Table S5. The information of Hi-C sequencing data in allotetraploid.

| | Read number | Total base | GC (%) | Q20 (%) | Q30 (%) |
|---|---|---|---|---|---|
| Tetraploid | 697,563,897 | 209,269,169,100 | 40.08 | 97.74 | 94.55 |

Table S6. The summary of full-length transcriptome.

| | Tetraploid |
|---|---|
| The sequencing data (Gb) | 30.49 |
| No. of subreads | 18,340,253 |
| Average length of subreads (bp) | 1,714 |
| N50 of subreads (bp) | 2,139 |
| Max of subreads (bp) | 125,630 |

Table S7. The statistics of genome assembly after polishing with Illumina data.

| | Tetraploid | |
|---|---|---|
| | Contig length (bp) | Contig number |
| N50 | 2,833,377 | 287 |
| N60 | 2,036,819 | 420 |
| N70 | 1,398,561 | 606 |
| N80 | 773,923 | 908 |
| N90 | 216,826 | 1,636 |
| Longest | 24,801,351 | 1 |
| Total | 3,164,612,733 | 6,179 |
| Length ≥ 1 kb | 3,164,612,733 | 6,179 |
| Length ≥ 2 kb | 3,164,612,733 | 6,179 |

| | | |
|---|---|---|
| Length ≥ 5 kb | 3,164,526,199 | 6,154 |

Table S8. The summary of genome correction and assembly based on analysis pipelines with combined with Nanopore, Illumina, and BioNano data.

| | Tetraploid | | | |
|---|---|---|---|---|
| | Contig Length (bp) | Contig Number | Scaffold Length (bp) | Scaffold Number |
| N50 | 2,392,008 | 335 | 4,659,828 | 190 |
| N60 | 1,746,760 | 488 | 3,532,762 | 269 |
| N70 | 1,146,864 | 707 | 2,392,721 | 376 |
| N80 | 642,325 | 1,067 | 1,263,640 | 552 |
| N90 | 190,858 | 1,939 | 252,354 | 1,051 |
| Longest contigs | 25,169,294 | 1 | 25,169,294 | 1 |
| Total | 3,129,657,713 | 6,562 | 3,157,327,662 | 5,594 |
| Length ≥ 1 kb | 3,129,657,413 | 6,557 | 3,157,327,362 | 5,589 |
| Length ≥ 2 kb | 3,129,656,153 | 6,556 | 3,157,326,102 | 5,588 |
| Length ≥ 5 kb | 3,129,567,385 | 6,529 | 3,157,237,334 | 5,561 |

Table S9. The statistics of valid mapping results of Hi-C data in the allotetraploid.

| | Tetraploid | |
|---|---|---|
| Unique paired alignments | 298,729,554 | 100% |
| Valid interaction pairs | 170,012,784 | 56.91% |
| Dangling end pairs | 55,776,626 | 18.67% |
| Re-ligation pairs | 6,216,096 | 2.08% |
| Self-cycle pairs | 62,516,010 | 20.93% |
| Dumped pairs | 4,208,038 | 1.41% |

Table S11. Summary of whole genome anchored by HI-C data.

| | Tetraploid | |
|---|---|---|
| | Scaffold | Contig |
| Number | 2,812 | 4,202 |
| Total length (bp) | 2,953,563,945 | 2,953,424,945 |
| N50 (bp) | 26,740,681 | 2,863,522 |
| N90 (bp) | 18,507,213 | 473,835 |
| Max length (bp) | 47,189,010 | 13,873,778 |

Table S12. The statistics of BUSCO completeness (v. 4.0).

| | Tetraploid |
|---|---|
| Complete BUSCOs | 3,234 (96.42%) |
| Complete and single-copy BUSCOs | 326 (9.72%) |
| Complete and duplicated BUSCOs | 2,908 (86.70%) |
| Fragmented BUSCOs | 28 (0.83%) |
| Missing BUSCOs | 92 (2.74%) |
| Total BUSCO groups searched | 3,354 |

Table S13. The gene prediction annotated with the three methods, including ab initio, homology-based, and transcriptome.

| Method | Software | Reference species | Gene number |
|---|---|---|---|
| Ab initio | Augustus | - | 105,604 |
| | SNAP | - | 343,969 |
| Homology-based | GeMoMa | *C. alburnus* | 72,399 |
| | | *C. auratus* | 82,228 |
| | | *C. idellus* | 73,800 |
| | | *D. rerio* | 74,029 |
| RNA-seq | GeneMarkS-T | - | 66,848 |
| | PASA | - | 18,843 |
| Integration | EVM | - | 89,224 |

Table S14. The gene number annotated with different databases.

| | Number of protein (length: 100 to 300 bp) | Number of protein (length >=300 bp) | All |
|---|---|---|---|
| GO | 13,476 | 36,412 | 50,533 |
| KEGG | 10,174 | 34,010 | 44,692 |
| KOG | 12,515 | 44,707 | 57,709 |
| TrEMBL | 22,829 | 60,975 | 84,838 |
| NR | 23,043 | 61,070 | 85,140 |
| Total | 23,087 | 61,086 | 85,214 |

Note: Database: NR (release: 202009, ftp://ftp.ncbi.nlm.nih.gov/blast/db), EggNOG (v 5.0, http://eggnog5.embl.de/download/eggnog_5.0/), GO (release: 20200615, http://geneontology.org), KEGG (release: 20191220, http://www.genome.jp/kegg), SWISS-PROT (release: 202005, http://ftp.ebi.ac.uk/pub/databases/swissprot), Pfam (v 33.1, http://pfam.xfam.org), and TrEMBL (release: 202005).

Table S15. The annotation of repeat sequences in assembly genome of the allotetraploid.

| Type | Number | Length | Rate (%) |
|---|---|---|---|
| ClassI:Retroelement | 1,384,049 | 399,184,599 | 13.52 |
| ClassI/LTR/Cassandra | 18 | 968 | 0 |

| | | | |
|---|---|---|---|
| ClassI/LTR/Caulimovirus | 116 | 7,079 | 0 |
| ClassI/LTR/Copia | 21,730 | 4,017,520 | 0.14 |
| ClassI/LTR/Gypsy | 303,798 | 115,327,313 | 3.9 |
| ClassI/LTR/Ngaro | 5,706 | 1,535,108 | 0.05 |
| ClassI/LTR/Pao | 39,750 | 6,501,377 | 0.22 |
| ClassI/LTR/RTE-X | 6 | 3,690 | 0 |
| ClassI/LTR/Unknown | 263,383 | 81,760,107 | 2.77 |
| ClassI/LTR/Viper | 453 | 53,343 | 0 |
| ClassI/DIRS | 75,043 | 39,162,874 | 1.33 |
| ClassI/LINE | 452,602 | 125,772,093 | 4.26 |
| ClassI/LTR/ERV | 143,286 | 16,358,997 | 0.55 |
| ClassI/LTR/Unknown | 211 | 15,333 | 0 |
| ClassI/SINE | 77,947 | 8,993,104 | 0.3 |
| ClassII:DNA transposon | 4,707,818 | 891,672,009 | 30.19 |
| ClassII/Academ | 3,362 | 455,378 | 0.02 |
| ClassII/CACTA | 889,991 | 135,626,089 | 4.59 |
| ClassII/Crypton | 119,558 | 20,096,613 | 0.68 |
| ClassII/Dada | 25,295 | 4,369,900 | 0.15 |
| ClassII/Ginger | 18,501 | 1,809,981 | 0.06 |
| ClassII/Helitron | 93,547 | 42,737,610 | 1.45 |
| ClassII/IS3EU | 71,267 | 13,207,372 | 0.45 |
| ClassII/Kolobok | 361,565 | 75,531,009 | 2.56 |
| ClassII/MITE | 168 | 6,361 | 0 |
| ClassII/Maverick | 21,257 | 4,138,970 | 0.14 |
| ClassII/Merlin | 72,427 | 12,164,419 | 0.41 |
| ClassII/Mutator | 48,682 | 5,922,626 | 0.2 |
| ClassII/Novosib | 30,823 | 2,688,731 | 0.09 |
| ClassII/P | 25,873 | 3,663,920 | 0.12 |
| ClassII/PIF-Harbinger | 241,606 | 47,052,928 | 1.59 |
| ClassII/PiggyBac | 87,524 | 23,482,746 | 0.8 |
| ClassII/Sola | 47,340 | 6,615,944 | 0.22 |
| ClassII/Tc1-Mariner | 491,526 | 111,068,062 | 3.76 |
| ClassII/Unknown | 685,250 | 132,985,035 | 4.5 |
| ClassII/Zator | 15,764 | 2,551,470 | 0.09 |
| ClassII/Zisupton | 67,735 | 9,921,152 | 0.34 |
| ClassII/hAT | 1,288,757 | 236,781,342 | 8.02 |
| Total | 6,091,867 | 1,289,350,257 | 43.65 |

Table S16. The statistics of SSR distribution in the allotetraploid.

| | Tetraploid | | | Common carp | | | Goldfish | | |
|---|---|---|---|---|---|---|---|---|---|
| | Number | Length (bp) | Rate (%) | Number | Length (bp) | Rate (%) | Number | Length (bp) | Rate (%) |
| Micro-satellite (1-9 bp in each unit) | 2,417,401 | 77,282,943 | 2.62 | 1,073,550 | 39,790,863 | 2.79 | 1,281,153 | 35,054,555 | 2.14 |
| Mini-satellite (10-99 bp in each unit) | 31,650 | 38,467,916 | 1.3 | 11,489 | 10,370,531 | 0.73 | 17,720 | 20,047,308 | 1.22 |
| Satellite (>=100 bp in each unit) | 36,275 | 77,856,373 | 2.64 | 17,192 | 14,412,844 | 1.01 | 15,228 | 17,319,650 | 1.06 |
| Total | 2,485,326 | 193,607,232 | 6.55 | 1,102,231 | 64,574,238 | 4.53 | 1,314,101 | 72,421,513 | 4.42 |

Table S17. The statistics of non-coding RNAs annotation.

| | Tetraploid |
|---|---|
| rRNA | 16,339 |
| tRNA | 29,790 |
| miRNA | 2,926 |
| snRNA | 1,074 |
| snoRNA | 364 |
| Total | 50,493 |

Table S18. Summary of the 11 genome sequences in our studies.

| | Species | Gene number | Database | Download website |
|---|---|---|---|---|
| Group 1 | *Cyprinus carpio haematopterus* | 44,626 | CNCB | https://bigd.big.ac.cn/gwh/Assembly/497/show |
| | *Carassius auratus* red var. | 43,144 | CNCB | https://bigd.big.ac.cn/gwh/Assembly/211/show |
| Group 2 | *Culter alburnus* | 30,443 | NCBI | https://www.ncbi.nlm.nih.gov/genome/?term=Culter+alburnus |
| | *Megalobrama amblycephala* | 29,994 | NCBI | https://www.ncbi.nlm.nih.gov/genome/?term=Megalobrama+amblycephala |
| Group 3 | *Takifugu rubripes* | 27,342 | NCBI | https://www.ncbi.nlm.nih.gov/genome/?term=Takifugu+rubripes |
| | *T. flavidus* | 29,408 | NCBI | https://www.ncbi.nlm.nih.gov/genome/?term=Takifugu+flavidus |
| Group 4 | *Oreochromis aureus* | 23,117 | NCBI | https://www.ncbi.nlm.nih.gov/genome/?term=Oreochromis+aureus |
| | *O. niloticus* | 26,329 | NCBI | https://www.ncbi.nlm.nih.gov/genome/?term=Oreochromis+niloticus |
| Group 5 | *Xiphophorus hellerii* | 30,235 | NCBI | https://www.ncbi.nlm.nih.gov/genome/?term=Xiphophorus+hellerii |
| | *X. maculatus* | 27,255 | NCBI | https://www.ncbi.nlm.nih.gov/genome/?term=Xiphophorus+maculatus |
| | *Danio rerio* | 25,592 | Ensemble | https://asia.ensembl.org/Danio_rerio/Info/Index |

Note: CNCB-NGDC: The China National Center for Bioinformation; NCBI: National Center for Biotechnology Information.

Table S19. The divergence time between the two inbred parents in the five hybrid groups.

| Species | Divergence time | Hybrid progeny | Genome data |
|---|---|---|---|
| *C. carpio* (♂) <br> *C. auratus* (♀) | 9.95 Mya (Luo et al. 2020) | (Liu et al. 2016) | (Xu et al. 2019) <br> (Luo et al. 2020) |
| *C. alburnus* (♂) <br> *M. amblycephala* (♀) | 12.74 Mya (Ren et al. 2019) | (Xiao et al. 2014) | (Ren et al. 2019) |
| *T. rubripes* (♂) <br> *T. flavidus* (♀) | 1.8–5.3 Mya (Yamanoue et al. 2008) | (Gao et al. 2013) | (Aparicio et al. 2002) <br> (Gao et al. 2014) |
| *O. aureus* (♂) <br> *O. niloticus* (♀) | 23.2 Mya (Bian et al. 2019) | (Hulata et al. 1993) | (Bian et al. 2019) <br> (Conte et al. 2017) |
| *X. maculatus* (♂) <br> *X. hellerii* (♀) | 3 Mya (Powell et al. 2020) | (Lu et al. 2020; Powell et al. 2020) | (Amores et al. 2014) <br> (Shen et al. 2016) |

Table S20. The statistics of genome assembly results in three versions of goldfish (*C. auratus*) and two versions of common carp (*C. carpio*) in published papers.

| | *C. auratus*[1] | *C. auratus*[2] | *C. auratus*[3] | *C. carpio*[4] | *C. carpio*[5] |
|---|---|---|---|---|---|
| Genome size (Gb) | 1.49 | 1.74 | 1.82 | 1.42 | 1.71 |
| Contig N50 (Mb) | 1.16 | 0.61 | 0.82 | 0.02 | 0.08 |
| Contig number | 4,433 | 5,888 | 9,415 | 18,518 | 53,088 |
| Scaffold N50 (Mb) | 34.79 | 31.84 | 22.76 | 1.71 | 7.83 |
| Scaffold number | 5,477 | 1,770 | 6,216 | 211 | 9,378 |

Note: [1] version 1 of *C. auratus* was downloaded from Jing Luo *et al.*

[2] version 2 of *C. auratus* was downloaded from Duo Chen *et al.*

[3] version 3 of *C. auratus* was downloaded from Zelin Chen *et al.*

[4] version 1 of *C. carpio* was downloaded from Peng Xu *et al.*

[5] version 2 of *C. carpio* was downloaded from Peng Xu *et al.*

Table S21. The statistics of BUSCO completeness (v. 4.0).

| | Goldfish | Common carp |
|---|---|---|
| Complete BUSCOs | 3,051 (90.97%) | 3,239 (96.57%) |
| Complete and single-copy BUSCOs | 1,783 (53.16%) | 1,180 (35.18%) |
| Complete and duplicated BUSCOs | 1,268 (37.81%) | 2,059 (61.39%) |
| Fragmented BUSCOs | 141 (4.20%) | 59 (1.76%) |
| Missing BUSCOs | 162 (4.83%) | 56 (1.67%) |
| Total BUSCO groups searched | 3,354 | 3,354 |

Note: Genome data of common carp was downloaded from Peng Xu *et al.* (Xu et al. 2019), while genome of goldfish

was downloaded from Jing Luo *et al.* (Luo et al. 2020).

Table S24. The statistics of simple sequence repeat (SSR) in the two parent genomes of the five hybrid groups.

| | Species | Micro-satellite (bp) | Mini-satellite (bp) | Satellite (bp) | Total (bp) |
|---|---|---|---|---|---|
| Group 1 | *C. carpio haematopterus* (♂) | 35,054,555 (2.14%) | 20,047,308 (1.22%) | 17,319,650 (1.06%) | 72,421,513 (4.42%) |
| | *C. auratus* red var. (♀) | 39,790,863 (2.79%) | 10,370,531 (0.73%) | 14,412,844 (1.01%) | 64,574,238 (4.53%) |
| | Difference rate | 0.65% | 0.49% | 0.05% | 0.11% |
| Group 2 | *C. alburnus* (♂) | 17,323,687 (1.70%) | 15,595,614 (1.53%) | 12,741,041 (1.25%) | 45,660,342 (4.49%) |
| | *M. amblycephala* (♀) | 20,210,000 (1.86%) | 16,800,380 (1.54%) | 14,349,405 (1.32%) | 51,359,785 (4.72%) |
| | Difference rate | 0.16% | 0.01% | 0.07% | 0.23% |
| Group 3 | *T. rubripes* (♂) | 8,906,314 (2.32%) | 8,741,217 (2.28%) | 3,846,105 (1.00%) | 21,493,636 (5.60%) |
| | *T. flavidus* (♀) | 8,377,149 (2.29%) | 6,687,367 (1.83%) | 3,486,428 (0.95%) | 18,550,944 (5.06%) |
| | Difference rate | 0.03% | 0.45% | 0.05% | 0.54% |
| Group 4 | *O. aureus* (♂) | 10,433,529 (1.04%) | 25,951,679 (2.58%) | 13,644,457 (1.36%) | 50,029,665 (4.98%) |
| | *O. niloticus* (♀) | 8,749,587 (0.87%) | 16,038,346 (1.59%) | 16,581,640 (1.65%) | 41,369,573 (4.11%) |
| | Difference rate | 0.17% | 0.99% | 0.29% | 0.87% |
| Group 5 | *X. maculatus* (♂) | 6,756,201 (0.96%) | 5,124,662 (0.73%) | 8,807,895 (1.25%) | 20,688,758 (2.94%) |
| | *X. hellerii* (♀) | 6,320,379 (0.86%) | 6,418,439 (0.88%) | 9,220,637 (1.26%) | 21,959,455 (3.00%) |
| | Difference rate | 0.10% | 0.15% | 0.01% | 0.06% |

Note: Micro-satellite represents the SSR with length range of 1-9 bp in each unit.

Mini-satellite represents the SSR with length range of 10-99 bp in each unit.

Satellite represents the SSR with length range of more than 100 bp in each unit.

Difference Rate: The absolute value of difference of the rate between the two hybrid parents.

Table S26. The information of whole genome re-sequencing using Illumina and DNB sequencing.

| Sample | Total raw data (Gb) | No. of raw reads | Total clean data (Gb) | No. of clean reads | Q20 | Sequencing platform |
|---|---|---|---|---|---|---|

| | | | | | | |
|---|---|---|---|---|---|---|
| RCC+CC | 94.72 | 631,470,296 | 94.22 | 631,443,860 | 97.30% | DNB |
| $F_1$_1_M | 107.80 | 718,693,536 | 106.24 | 711,358,208 | 97.05% | DNB |
| $F_1$_1_G | 95.64 | 637,630,038 | 94.67 | 633,945,714 | 96.94% | DNB |
| $F_1$_2_M | 51.92 | 346,111,372 | 51.55 | 345,961,134 | 95.26% | DNB |
| $F_1$_2_G | 50.30 | 335,359,280 | 49.91 | 335,192,004 | 94.71% | DNB |
| $F_{22}$_3_M | 52.21 | 348,060,172 | 51.86 | 347,908,674 | 95.43% | DNB |
| $F_{22}$_3_G | 50.93 | 339,550,666 | 50.63 | 339,404,560 | 95.09% | DNB |
| $F_{22}$_4_M | 53.61 | 357,424,580 | 53.27 | 357,254,366 | 95.32% | DNB |
| $F_{22}$_4_G | 51.80 | 345,343,444 | 51.60 | 345,148,122 | 94.38% | DNB |
| $F_{22}$_5_M | 52.98 | 353,193,512 | 52.69 | 352,982,856 | 95.99% | DNB |
| $F_{22}$_5_G | 53.63 | 357,502,876 | 53.34 | 357,326,226 | 96.33% | DNB |
| RCC+CC | 133.18 | 887,886,184 | 131.13 | 880,025,966 | 97.07% | Illumina |
| $F_{24}$_1_F | 95.92 | 639,472,492 | 70.16 | 467,741,084 | 98.40% | Illumina |
| $F_{24}$_2_F | 97.06 | 647,060,158 | 72.24 | 481,626,820 | 98.50% | Illumina |
| $F_{24}$_3_F | 94.17 | 627,781,724 | 67.22 | 448,105,996 | 98.37% | Illumina |
| $F_{24}$_4_F | 100.76 | 671,715,452 | 74.82 | 498,792,298 | 98.46% | Illumina |
| $F_{24}$_5_F | 96.37 | 642,461,076 | 70.62 | 470,781,424 | 98.50% | Illumina |
| Total | 1333.00 | 8,886,716,858 | 1196.17 | 8,004,999,312 | | |

Note: The symbols of "F", "M", and "G" represent caudal fin, muscle, and gonad tissues, respectively. Sample "RCC+CC" represent the DNA of muscle of goldfish and common carp mixed in equal amounts.

Table S27. The unequal HRs in muscle, gonad, and caudal fin tissues of the intergeneric $F_1$, the allotetraploid $F_{22}$, and $F_{24}$ individuals based on Illumina and DNB data.

| Sample | $R_{gene\ copy} > C_{gene\ copy}$[a] | | $R_{gene\ copy} < C_{gene\ copy}$[b] | | Total | |
|---|---|---|---|---|---|---|
| | One HGP | Three contiguous HGPs | One HGP | Three contiguous HGPs | One HGP | Three contiguous HGPs |
| $F_1$_1_M | 50 (0.38%) | 15 (0.11%) | 31 (0.23%) | 6 (0.05%) | 81 (0.61%) | 21 (0.16%) |
| $F_1$_1_G | 45 (0.34%) | 12 (0.09%) | 38 (0.29%) | 4 (0.03%) | 83 (0.63%) | 16 (0.12%) |
| $F_1$_2_M | 71 (0.54%) | 13 (0.10%) | 34 (0.26%) | 5 (0.04%) | 105 (0.79%) | 18 (0.14%) |
| $F_1$_2_G | 77 (0.58%) | 35 (0.26%) | 34 (0.26%) | 19 (0.14%) | 111 (0.84%) | 54 (0.41%) |
| $F_{22}$_3_M | 54 (0.41%) | 6 (0.05%) | 37 (0.28%) | 9 (0.07%) | 91 (0.69%) | 15 (0.11%) |
| $F_{22}$_3_G | 62 (0.47%) | 9 (0.07%) | 31 (0.23%) | 8 (0.06%) | 93 (0.70%) | 17 (0.13%) |
| $F_{22}$_4_M | 60 (0.45%) | 15 (0.11%) | 24 (0.18%) | 4 (0.03%) | 84 (0.63%) | 19 (0.14%) |
| $F_{22}$_4_G | 62 (0.47%) | 7 (0.05%) | 33 (0.25%) | 9 (0.07%) | 95 (0.72%) | 16 (0.12%) |
| $F_{22}$_5_M | 61 (0.46%) | 17 (0.13%) | 27 (0.20%) | 3 (0.02%) | 88 (0.66%) | 20 (0.15%) |
| $F_{22}$_5_G | 81 (0.61%) | 8 (0.06%) | 37 (0.28%) | 8 (0.06%) | 118 | 16 (0.12%) |

| | | | | | | |
|---|---|---|---|---|---|---|
| | | | | | (0.89%) | |
| $F_{24}$_1_F | 220 (1.66%) | 152 (1.15%) | 96 (0.72%) | 15 (0.11%) | 316 (2.39%) | 167 (1.26%) |
| $F_{24}$_2_F | 223 (1.68%) | 154 (1.16%) | 146 (1.10%) | 18 (0.14%) | 369 (2.79%) | 172 (1.30%) |
| $F_{24}$_3_F | 73 (0.55%) | 9 (0.07%) | 163 (1.23%) | 17 (0.13%) | 236 (1.78%) | 26 (0.20%) |
| $F_{24}$_4_F | 80 (0.60%) | 15 (0.11%) | 83 (0.63%) | 10 (0.08%) | 163 (1.23%) | 25 (0.19%) |
| $F_{24}$_5_F | 194 (1.46%) | 133 (1.00%) | 85 (0.64%) | 18 (0.14%) | 279 (2.11%) | 151 (1.14%) |

Note: [a] represents that mapped read number in each base of homoeolog R was twice than ones in homoeolog C.

[b] represents that mapped read number in each base of homoeolog C was twice than ones in homoeolog R.

Table S28. Depth of the allotetraploid reads in regions of the CACTA and hAT superfamilies.

| | CACTA superfamily | | hAT superfamily | |
|---|---|---|---|---|
| | Subgenome R | Subgenome C | Subgenome R | Subgenome C |
| AT_Chr01 | 35.27 | 33.85 | 34.67 | 33.96 |
| AT_Chr02 | 36.43 | 30.01 | 34.07 | 31.20 |
| AT_Chr03 | 34.06 | 33.09 | 32.50 | 32.85 |
| AT_Chr04 | 32.61 | 34.73 | 30.43 | 35.80 |
| AT_Chr05 | 33.62 | 32.13 | 32.80 | 31.58 |
| AT_Chr06 | 33.28 | 32.50 | 33.46 | 32.16 |
| AT_Chr07 | 32.21 | 34.54 | 31.54 | 34.80 |
| AT_Chr08 | 26.93 | 33.59 | 28.35 | 33.35 |
| AT_Chr09 | 33.95 | 32.05 | 32.92 | 32.22 |
| AT_Chr10 | 31.81 | 34.81 | 31.39 | 34.76 |
| AT_Chr11 | 34.24 | 33.97 | 34.01 | 34.20 |
| AT_Chr12 | 33.57 | 33.67 | 33.41 | 33.33 |
| AT_Chr13 | 30.45 | 35.02 | 30.44 | 34.33 |
| AT_Chr14 | 34.37 | 33.73 | 33.02 | 34.29 |
| AT_Chr15 | 34.60 | 32.29 | 33.43 | 32.63 |
| AT_Chr16 | 32.57 | 33.44 | 30.11 | 33.58 |
| AT_Chr17 | 33.61 | 33.25 | 33.05 | 33.74 |
| AT_Chr18 | 34.83 | 34.02 | 33.26 | 33.31 |
| AT_Chr19 | 33.43 | 35.15 | 32.33 | 34.59 |
| AT_Chr20 | 32.44 | 33.71 | 31.23 | 33.60 |
| AT_Chr21 | 32.33 | 34.61 | 30.73 | 34.83 |
| AT_Chr22 | 35.68 | 34.29 | 34.44 | 34.85 |
| AT_Chr23 | 35.50 | 33.69 | 34.32 | 33.87 |
| AT_Chr24 | 35.48 | 33.09 | 32.25 | 33.56 |
| AT_Chr25 | 31.62 | 33.13 | 30.60 | 33.38 |
| AT_Chr26 | 31.08 | 35.65 | 30.73 | 35.18 |

| | | | | |
|---|---|---|---|---|
| AT_Chr27 | 35.65 | 34.14 | 34.40 | 34.75 |
| AT_Chr28 | 34.94 | 33.84 | 32.80 | 34.87 |
| AT_Chr29 | 33.67 | 30.37 | 32.82 | 30.37 |
| AT_Chr30 | 35.22 | 33.50 | 34.03 | 33.40 |
| AT_Chr31 | 32.82 | 34.32 | 31.32 | 34.46 |
| AT_Chr32 | 33.55 | 33.97 | 33.46 | 33.34 |
| AT_Chr33 | 33.27 | 32.82 | 33.18 | 32.49 |
| AT_Chr34 | 29.74 | 32.97 | 30.61 | 33.53 |
| AT_Chr35 | 35.00 | 33.11 | 33.18 | 32.82 |
| AT_Chr36 | 31.46 | 34.37 | 31.33 | 33.79 |
| AT_Chr37 | 35.56 | 32.13 | 33.63 | 32.81 |
| AT_Chr38 | 33.71 | 34.00 | 32.47 | 34.66 |
| AT_Chr39 | 34.14 | 33.95 | 34.06 | 33.89 |
| AT_Chr40 | 35.09 | 36.36 | 33.96 | 36.75 |
| AT_Chr41 | 34.28 | 33.40 | 32.84 | 33.64 |
| AT_Chr42 | 30.13 | 34.42 | 30.22 | 34.16 |
| AT_Chr43 | 27.08 | 35.06 | 27.79 | 35.77 |
| AT_Chr44 | 35.49 | 32.05 | 35.46 | 32.18 |
| AT_Chr45 | 34.65 | 32.89 | 35.93 | 32.62 |
| AT_Chr46 | 33.97 | 34.20 | 32.78 | 34.17 |
| AT_Chr47 | 33.70 | 31.93 | 33.44 | 31.64 |
| AT_Chr48 | 34.28 | 33.02 | 32.53 | 32.67 |
| AT_Chr49 | 34.67 | 31.64 | 33.24 | 31.20 |
| AT_Chr50 | 34.69 | 34.24 | 32.39 | 33.99 |
| Mean depth | 33.44 | 33.53 | 32.48 | 33.59 |

Table S29. The methyl-seq data in embryos of the allotetraploid lineage.

| Samples | Raw Reads | Clean Reads | Raw Base (Gb) | Clean Base (Gb) | Q20 (%) | Q30 (%) |
|---|---|---|---|---|---|---|
| RCC-BL | 148,500,510 | 147,673,056 | 44.55 | 44.30 | 96.25 | 89.95 |
| RCC-G | 151,675,489 | 150,622,630 | 45.50 | 45.19 | 96.43 | 90.47 |
| RCC-S | 158,653,911 | 157,459,065 | 47.60 | 47.24 | 96.38 | 90.51 |
| RCC-H | 162,051,655 | 160,866,489 | 48.62 | 48.26 | 96.83 | 91.22 |
| CC-BL | 156,933,357 | 155,825,068 | 47.08 | 46.75 | 96.56 | 90.69 |
| CC-G | 157,615,618 | 156,629,113 | 47.28 | 46.99 | 96.38 | 90.38 |
| CC-S | 175,492,895 | 174,433,926 | 52.65 | 52.33 | 95.19 | 88.09 |
| CC-H | 175,939,800 | 174,974,803 | 52.78 | 52.49 | 96.56 | 90.76 |
| $F_1$-BL | 318,300,146 | 316,649,325 | 95.49 | 94.99 | 95.37 | 88.19 |
| $F_1$-G | 327,271,316 | 322,974,624 | 98.18 | 96.89 | 96.78 | 91.18 |
| $F_1$-S | 332,091,901 | 328,999,983 | 99.63 | 98.70 | 96.44 | 90.56 |
| $F_1$-H | 321,510,765 | 318,545,270 | 96.45 | 95.56 | 96.76 | 91.02 |
| $F_{22}$-BL | 315,049,909 | 313,194,047 | 94.51 | 93.96 | 95.69 | 88.76 |
| $F_{22}$-G | 318,346,753 | 316,566,288 | 95.50 | 94.97 | 96.50 | 90.56 |
| $F_{22}$-S | 360,299,548 | 358,297,426 | 108.09 | 107.49 | 96.43 | 90.53 |

| | | | | | | |
|---|---|---|---|---|---|---|
| $F_{22}$-H | 360,123,069 | 357,828,584 | 108.04 | 107.35 | 96.77 | 91.11 |
| Total | 3,939,856,642 | 3,911,539,697 | 1,182 | 1,173 | | |

Note: The symbols of “BL”, “G”, “S”, and “H” represent periods of blastula, gastrula, segmentation, and hatching, respectively.

Table S30. The mapping information in methyl-seq data of the allotetraploid lineage.

| | NO. of total read pairs | NO. of uniquely mapped read pairs | Uniquely mapped efficiency | NO. of multi-mapped read pairs |
|---|---|---|---|---|
| RCC-BL | 148,500,510 | 78,190,306 | 52.70% | 8,107,793 |
| RCC-G | 151,675,489 | 83,853,223 | 55.30% | 6,863,173 |
| RCC-S | 158,653,911 | 87,574,242 | 55.20% | 7,129,332 |
| RCC-H | 162,051,655 | 92,797,363 | 57.30% | 7,735,671 |
| CC-BL | 156,933,357 | 80,146,974 | 51.10% | 3,600,577 |
| CC-G | 157,615,618 | 77,252,074 | 49.00% | 3,078,872 |
| CC-S | 177,318,271 | 64,065,786 | 36.10% | 2,947,456 |
| CC-H | 175,939,800 | 90,673,699 | 51.50% | 3,487,238 |
| $F_1$-BL | 318,300,146 | 150,829,476 | 47.40% | 8,052,171 |
| $F_1$-G | 327,271,316 | 177,981,859 | 54.40% | 11,353,812 |
| $F_1$-S | 332,091,901 | 172,903,881 | 52.10% | 11,465,261 |
| $F_1$-H | 321,191,475 | 167,652,612 | 52.20% | 11,445,224 |
| $F_{22}$-BL | 315,049,909 | 156,784,396 | 49.80% | 9,093,423 |
| $F_{22}$-G | 318,346,753 | 166,979,148 | 52.50% | 9,930,775 |
| $F_{22}$-S | 360,299,548 | 192,017,168 | 53.30% | 11,672,801 |
| $F_{22}$-H | 360,123,069 | 192,073,062 | 53.30% | 11,702,901 |
| Total | 3,941,362,728 | 2,031,775,269 | | 127,666,480 |

Note: The symbols of “BL”, “G”, “S”, and “H” represent periods of blastula, gastrula, segmentation, and hatching, respectively.

Table S31.The summary of 5-methylcytosine (5mC) of the allotetraploid lineage.

| | Total number of cytosine | Methylated cytosines | | | | Unmethylated cytosines | | | |
|---|---|---|---|---|---|---|---|---|---|
| | | CpG | CHG | CHH | Unknown | CpG | CHG | CHH | Unknown |
| RCC-BL | 4,215,296,346 | 304,458,375 (81.60%) | 8,793,992 (0.90%) | 27,796,489 (1.00%) | 109,494 (3.40%) | 68,638,792 | 919,886,489 | 2,885,722,209 | 3,094,829 |
| RCC-G | 4,100,358,011 | 285,056,760 (82.30%) | 2,557,060 (0.30%) | 7,978,649 (0.30%) | 97,033 (2.80%) | 61,235,324 | 882,172,255 | 2,861,357,963 | 3,309,766 |
| RCC-S | 4,294,902,723 | 300,030,950 (82.30%) | 2,759,086 (0.30%) | 8,506,427 (0.30%) | 105,954 (3.00%) | 64,705,579 | 926,727,542 | 2,992,173,139 | 3,421,849 |
| RCC-H | 4,554,821,500 | 291,007,568 (77.20%) | 3,172,994 (0.30%) | 9,309,095 (0.30%) | 101,245 (2.70%) | 85,711,759 | 974,583,453 | 3,191,036,631 | 3,708,165 |
| CC-BL | 3,882,109,088 | 241,641,491 (80.80%) | 2,749,315 (0.30%) | 9,229,850 (0.30%) | 78,640 (2.60%) | 57,369,717 | 839,227,900 | 2,731,890,815 | 2,971,103 |
| CC-G | 3,683,983,187 | 226,382,019 (80.50%) | 2,661,528 (0.30%) | 8,845,674 (0.30%) | 74,377 (2.50%) | 54,807,941 | 795,882,098 | 2,595,403,927 | 2,913,917 |
| CC-S | 3,229,599,549 | 204,006,365 (79.80%) | 2,812,310 (0.40%) | 9,028,130 (0.40%) | 63,452 (2.50%) | 51,568,646 | 709,884,135 | 2,252,299,963 | 2,472,742 |
| CC-H | 4,365,532,488 | 246,640,103 (75.90%) | 3,459,984 (0.40%) | 10,753,558 (0.30%) | 80,161 (2.30%) | 78,398,705 | 935,212,146 | 3,091,067,992 | 3,423,903 |
| $F_1$-BL | 6,931,266,762 | 418,488,054 (81.10%) | 4,525,659 (0.30%) | 15,389,654 (0.30%) | 144,365 (2.40%) | 97,587,707 | 1,453,383,763 | 4,941,891,925 | 5,813,828 |
| $F_1$-G | 8,547,812,430 | 562,444,022 (81.50%) | 5,498,597 (0.30%) | 17,865,791 (0.30%) | 188,908 (2.80%) | 128,081,274 | 1,842,337,291 | 5,991,585,455 | 6,656,052 |
| $F_1$-S | 8,704,235,871 | 576,700,169 (79.00%) | 6,038,133 (0.30%) | 18,721,369 (0.30%) | 182,076 (2.70%) | 153,211,591 | 1,910,076,769 | 6,039,487,840 | 6,478,504 |

| | | | | | | | | | |
|---|---|---|---|---|---|---|---|---|---|
| $F_1$-H | 8,473,016,342 | 526,083,744 (75.60%) | 7,072,951 (0.40%) | 20,776,452 (0.40%) | 180,548 (2.60%) | 169,587,236 | 1,853,257,769 | 5,896,238,190 | 6,693,637 |
| $F_{22}$-BL | 7,278,942,007 | 453,794,199 (82.50%) | 4,968,217 (0.30%) | 16,695,201 (0.30%) | 161,313 (2.50%) | 96,494,118 | 1,530,587,579 | 5,176,402,693 | 6,165,016 |
| $F_{22}$-G | 7,997,342,395 | 518,215,737 (82.40%) | 5,456,517 (0.30%) | 17,957,496 (0.30%) | 182,658 (2.70%) | 110,611,562 | 1,707,244,019 | 5,637,857,064 | 6,570,272 |
| $F_{22}$-S | 9,332,084,615 | 622,247,189 (82.50%) | 6,573,252 (0.30%) | 21,071,663 (0.30%) | 215,647 (2.80%) | 131,958,541 | 2,016,804,334 | 6,533,429,636 | 7,501,181 |
| $F_{22}$-H | 9,417,947,069 | 581,001,859 (77.20%) | 7,132,396 (0.40%) | 21,549,088 (0.30%) | 203,097 (2.60%) | 171,341,219 | 2,027,406,996 | 6,609,515,511 | 7,579,257 |
| Total | 99,009 million | 6,358 million | 76 million | 241 million | 2 million | 1,581 million | 21,324 million | 69,427 million | 78 million |

Note: The symbols of “BL”, “G”, “S”, and “H” represent periods of blastula, gastrula, segmentation, and hatching, respectively.

Table S32. The information of transcriptomic data of goldfish (RCC), common carp (CC), intergeneric $F_1$ ($F_1$), and allotetraploid $F_{22}$ ($F_{22}$).

| Sample | No. raw reads | No. clean reads | Raw base (Gb) | Clean base (Gb) | Q20 (%) | Q30 (%) |
|---|---|---|---|---|---|---|
| RCC-BL-1 | 27,809,826 | 27,643,652 | 8.34 | 8.29 | 97.72 | 93.81 |
| RCC-BL-2 | 29,978,607 | 29,797,063 | 8.99 | 8.94 | 97.79 | 94.00 |
| RCC-BL-3 | 28,371,241 | 28,209,427 | 8.51 | 8.46 | 97.79 | 94.00 |
| RCC-G-1 | 34,050,452 | 33,785,353 | 10.22 | 10.14 | 97.81 | 93.99 |
| RCC-G-2 | 30,950,221 | 30,768,782 | 9.29 | 9.23 | 97.85 | 94.12 |
| RCC-G-3 | 27,940,977 | 27,790,217 | 8.38 | 8.34 | 97.68 | 93.72 |
| RCC-S-1 | 26,086,470 | 25,928,867 | 7.83 | 7.78 | 97.37 | 93.28 |
| RCC-S-2 | 27,029,424 | 26,807,041 | 8.11 | 8.04 | 97.31 | 93.02 |
| RCC-S-3 | 34,784,463 | 34,240,991 | 10.44 | 10.27 | 97.53 | 93.66 |
| RCC-H-1 | 26,129,239 | 24,548,317 | 7.84 | 7.36 | 96.65 | 92.15 |
| RCC-H-2 | 32,287,295 | 31,122,770 | 9.69 | 9.34 | 96.98 | 92.78 |
| RCC-H-3 | 26,453,579 | 25,148,519 | 7.94 | 7.54 | 97.17 | 93.08 |
| RCC-BA-1 | 52,868,317 | 51,969,556 | 15.86 | 15.59 | 95.64 | 88.91 |
| RCC-BA-2 | 38,664,235 | 38,045,607 | 11.60 | 11.41 | 95.27 | 88.14 |
| RCC-BA-3 | 49,778,259 | 49,081,363 | 14.94 | 14.73 | 95.21 | 87.90 |
| CC-BL-1 | 33,254,451 | 32,495,652 | 9.98 | 9.75 | 97.85 | 94.23 |
| CC-BL-2 | 27,561,890 | 26,483,123 | 8.27 | 7.94 | 97.69 | 93.88 |
| CC-BL-3 | 24,856,256 | 24,218,858 | 7.46 | 7.27 | 97.54 | 93.57 |
| CC-G-1 | 35,905,956 | 34,587,075 | 10.77 | 10.38 | 97.78 | 94.11 |
| CC-G-2 | 28,493,743 | 27,562,758 | 8.55 | 8.27 | 97.67 | 93.87 |
| CC-G-3 | 34,604,643 | 33,404,311 | 10.38 | 10.02 | 97.70 | 93.91 |
| CC-S-1 | 23,920,980 | 23,241,721 | 7.18 | 6.97 | 97.40 | 93.34 |
| CC-S-2 | 29,388,031 | 28,403,922 | 8.82 | 8.52 | 97.36 | 93.23 |
| CC-S-3 | 29,608,258 | 28,496,656 | 8.88 | 8.55 | 97.44 | 93.44 |
| CC-H-1 | 33,422,406 | 32,560,591 | 10.03 | 9.77 | 97.75 | 93.96 |
| CC-H-2 | 27,409,544 | 26,750,235 | 8.22 | 8.03 | 97.87 | 94.25 |
| CC-H-3 | 26,021,750 | 25,436,148 | 7.81 | 7.63 | 97.5 | 93.38 |
| CC-BA-1 | 51,316,073 | 50,495,016 | 15.40 | 15.15 | 95.25 | 88.01 |
| CC-BA-2 | 37,503,574 | 36,941,020 | 11.26 | 11.09 | 94.75 | 86.88 |
| CC-BA-3 | 47,704,067 | 47,036,210 | 14.32 | 14.12 | 95.27 | 88.12 |
| $F_1$-BL-1 | 32,523,829 | 32,290,647 | 9.76 | 9.69 | 97.52 | 93.46 |
| $F_1$-BL-2 | 27,783,566 | 27,638,831 | 8.34 | 8.29 | 97.56 | 93.63 |
| $F_1$-BL-3 | 27,254,009 | 27,069,878 | 8.18 | 8.12 | 97.66 | 93.84 |
| $F_1$-G-1 | 36,380,313 | 36,107,313 | 10.91 | 10.83 | 97.34 | 93.13 |
| $F_1$-G-2 | 25,465,047 | 25,241,061 | 7.64 | 7.57 | 97.58 | 93.75 |

| | | | | | | |
|---|---|---|---|---|---|---|
| $F_1$-G-3 | 32,225,121 | 32,015,039 | 9.67 | 9.60 | 97.39 | 93.34 |
| $F_1$-S-1 | 29,735,992 | 29,584,246 | 8.92 | 8.88 | 97.41 | 93.39 |
| $F_1$-S-2 | 30,651,799 | 30,478,029 | 9.20 | 9.14 | 97.26 | 92.90 |
| $F_1$-S-3 | 29,589,365 | 29,256,885 | 8.88 | 8.78 | 97.31 | 93.16 |
| $F_1$-H-1 | 31,194,026 | 29,826,389 | 9.36 | 8.95 | 97.01 | 92.74 |
| $F_1$-H-2 | 28,336,760 | 27,568,678 | 8.50 | 8.27 | 96.99 | 92.62 |
| $F_1$-H-3 | 35,669,468 | 34,569,684 | 10.70 | 10.37 | 97.10 | 92.85 |
| $F_1$-BA-1 | 30,409,369 | 30,014,047 | 9.12 | 9.00 | 94.91 | 87.10 |
| $F_1$-BA-2 | 46,724,038 | 45,836,281 | 14.02 | 13.75 | 95.13 | 87.79 |
| $F_1$-BA-3 | 48,788,720 | 47,910,523 | 14.64 | 14.38 | 95.28 | 88.06 |
| $F_{22}$-BL-1 | 23,848,099 | 22,632,682 | 7.15 | 6.79 | 97.41 | 93.31 |
| $F_{22}$-BL-2 | 32,459,076 | 31,820,875 | 9.74 | 9.55 | 97.36 | 93.21 |
| $F_{22}$-BL-3 | 32,047,597 | 31,215,147 | 9.61 | 9.36 | 97.50 | 93.46 |
| $F_{22}$-G-1 | 32,649,167 | 31,786,080 | 9.79 | 9.54 | 97.74 | 94.03 |
| $F_{22}$-G-2 | 34,007,161 | 33,165,306 | 10.20 | 9.95 | 97.60 | 93.68 |
| $F_{22}$-G-3 | 34,987,535 | 34,334,254 | 10.50 | 10.30 | 97.72 | 93.98 |
| $F_{22}$-S-1 | 30,109,906 | 29,486,446 | 9.03 | 8.85 | 97.73 | 93.97 |
| $F_{22}$-S-2 | 24,563,076 | 24,087,791 | 7.37 | 7.23 | 97.64 | 93.75 |
| $F_{22}$-S-3 | 32,628,063 | 31,881,830 | 9.79 | 9.56 | 97.37 | 93.14 |
| $F_{22}$-H-1 | 28,546,101 | 27,795,264 | 8.56 | 8.34 | 97.98 | 94.44 |
| $F_{22}$-H-2 | 34,012,037 | 33,310,101 | 10.20 | 9.99 | 98.00 | 94.48 |
| $F_{22}$-H-3 | 31,390,287 | 30,270,442 | 9.42 | 9.08 | 97.68 | 93.81 |
| $F_{22}$-BA-1 | 36,837,821 | 36,137,902 | 11.06 | 10.85 | 94.57 | 86.47 |
| $F_{22}$-BA-2 | 39,427,999 | 38,718,295 | 11.82 | 11.61 | 95.97 | 89.62 |
| $F_{22}$-BA-3 | 60,934,210 | 59,898,328 | 18.28 | 17.97 | 95.44 | 88.38 |
| Total | 1,985,333,784 | 1,944,949,095 | 595.67 | 583.52 | / | / |

Note: the symbols of “BL”, “G”, “S”, “H”, “L”, and “BA” represent periods of blastula, gastrula, segmentation, and hatching, and liver and barbel tissues, respectively.

Table S33. Summary of mapped reads in transcriptome data.

| Sample | No. of total clean reads | No. of unique mapped reads | No. of total mapped reads |
|---|---|---|---|
| RCC-BL-1 | 27,643,652 | 15,139,639 (54.77%) | 17,116,949 (61.92%) |
| RCC-BL-2 | 29,797,063 | 17,932,027 (60.18%) | 20,479,521 (68.73%) |
| RCC-BL-3 | 28,209,427 | 17,187,076 (60.93%) | 19,362,950 (68.64%) |
| RCC-G-1 | 33,785,353 | 20,327,073 (60.17%) | 23,558,526 (69.73%) |
| RCC-G-2 | 30,768,782 | 17,740,975 (57.66%) | 20,753,543 (67.45%) |
| RCC-G-3 | 27,790,217 | 17,005,957 (61.19%) | 19,733,833 (71.01%) |
| RCC-S-1 | 25,928,867 | 14,702,702 (56.70%) | 17,146,759 (66.13%) |

| | | | |
|---|---|---|---|
| RCC-S-2 | 26,807,041 | 15,351,892 (57.27%) | 17,781,110 (66.33%) |
| RCC-S-3 | 34,240,991 | 18,264,126 (53.34%) | 21,164,356 (61.81%) |
| RCC-H-1 | 24,548,317 | 17,316,034 (70.54%) | 19,960,236 (81.31%) |
| RCC-H-2 | 31,122,770 | 22,326,184 (71.74%) | 25,595,366 (82.24%) |
| RCC-H-3 | 25,148,519 | 18,177,062 (72.28%) | 20,765,132 (82.57%) |
| RCC-L-1 | 27,307,425 | 20,813,113 (76.22%) | 25,144,676 (92.08%) |
| RCC-L-2 | 26,819,542 | 20,436,491 (76.20%) | 24,684,706 (92.04%) |
| RCC-L-3 | 21,965,365 | 17,335,941 (78.92%) | 19,683,163 (89.61%) |
| RCC-BA-1 | 51,969,556 | 36,484,862 (69.01%) | 41,819,901 (80.47%) |
| RCC-BA-2 | 38,045,607 | 28,483,145 (73.67%) | 32,175,169 (84.57%) |
| RCC-BA-3 | 49,081,363 | 36,854,267 (74.04%) | 41,635,720 (84.83%) |
| CC-BL-1 | 29,386,495 | 24,035,070 (81.79%) | 26,809,299 (91.23%) |
| CC-BL-2 | 32,495,652 | 26,748,499 (82.31%) | 29,736,771 (91.51%) |
| CC-BL-3 | 26,483,123 | 21,642,999 (81.72%) | 24,144,663 (91.17%) |
| CC-G-1 | 34,587,075 | 27,460,958 (79.40%) | 31,114,532 (89.96%) |
| CC-G-2 | 27,562,758 | 21,829,354 (79.20%) | 24,751,356 (89.80%) |
| CC-G-3 | 33,404,311 | 26,520,332 (79.39%) | 30,043,837 (89.94%) |
| CC-S-1 | 27,968,494 | 21,296,110 (76.14%) | 24,570,321 (87.85%) |
| CC-S-2 | 28,403,922 | 21,583,665 (75.99%) | 24,941,483 (87.81%) |
| CC-S-3 | 28,496,656 | 21,662,042 (76.02%) | 25,020,063 (87.80%) |
| CC-H-1 | 32,560,591 | 25,950,192 (79.70%) | 29,721,307 (91.28%) |
| CC-H-2 | 26,750,235 | 21,387,145 (79.95%) | 24,460,414 (91.44%) |
| CC-H-3 | 25,436,148 | 20,216,709 (79.48%) | 23,195,223 (91.19%) |
| CC-L-1 | 36,893,941 | 24,289,846 (65.84%) | 33,285,713 (90.22%) |
| CC-L-2 | 27,790,630 | 19,108,852 (68.76%) | 24,380,719 (87.73%) |
| CC-L-3 | 31,851,346 | 15,690,065 (49.26%) | 21,977,428 (69.00%) |
| CC-BA-1 | 50,495,016 | 37,285,808 (72.66%) | 43,102,545 (85.36%) |
| CC-BA-2 | 36,941,020 | 27,001,395 (72.00%) | 31,148,668 (84.32%) |
| CC-BA-3 | 47,036,210 | 34,946,093 (73.26%) | 40,352,364 (85.79%) |
| $F_1$-BL-1 | 27,069,878 | 18,296,564 (67.59%) | 20,800,494 (76.84%) |
| $F_1$-BL-2 | 32,290,647 | 21,647,069 (67.04%) | 24,515,059 (75.92%) |
| $F_1$-BL-3 | 27,638,831 | 19,020,152 (68.82%) | 21,508,538 (77.82%) |
| $F_1$-G-1 | 36,107,313 | 21,563,401 (59.72%) | 27,889,288 (77.24%) |
| $F_1$-G-2 | 25,241,061 | 16,065,077 (63.65%) | 18,832,355 (74.61%) |
| $F_1$-G-3 | 32,015,039 | 19,865,410 (62.05%) | 23,473,426 (73.32%) |
| $F_1$-S-1 | 29,584,246 | 20,845,265 (70.46%) | 23,927,738 (80.88%) |
| $F_1$-S-2 | 30,478,029 | 21,537,827 (70.67%) | 24,656,725 (80.90%) |
| $F_1$-S-3 | 29,256,885 | 20,369,451 (69.62%) | 23,279,703 (79.57%) |

| | | | |
|---|---|---|---|
| $F_1$-H-1 | 29,826,389 | 21,890,802 (73.39%) | 25,107,854 (84.18%) |
| $F_1$-H-2 | 27,568,678 | 19,791,994 (71.79%) | 23,687,008 (85.92%) |
| $F_1$-H-3 | 34,569,684 | 25,778,547 (74.57%) | 29,422,258 (85.11%) |
| $F_1$-L-1 | 35,570,194 | 25,601,297 (71.97%) | 33,019,811 (92.83%) |
| $F_1$-L-2 | 23,998,187 | 17,706,241 (73.78%) | 22,390,308 (93.30%) |
| $F_1$-L-3 | 24,979,808 | 14,074,798 (56.34%) | 22,689,159 (90.83%) |
| $F_1$-BA-1 | 30,014,047 | 21,963,420 (72.23%) | 25,280,831 (84.23%) |
| $F_1$-BA-2 | 45,836,281 | 34,443,006 (73.72%) | 39,281,692 (85.70%) |
| $F_1$-BA-3 | 47,910,523 | 35,625,772 (73.02%) | 40,570,630 (84.68%) |
| $F_{22}$-BL-1 | 27,740,382 | 21,646,115 (78.03%) | 24,475,339 (88.23%) |
| $F_{22}$-BL-2 | 31,820,875 | 24,628,832 (77.40%) | 27,865,540 (87.57%) |
| $F_{22}$-BL-3 | 31,215,147 | 24,515,511 (78.54%) | 27,644,134 (88.56%) |
| $F_{22}$-G-1 | 34,334,254 | 27,404,621 (79.82%) | 30,773,791 (89.63%) |
| $F_{22}$-G-2 | 31,786,080 | 25,458,082 (80.09%) | 28,572,507 (89.89%) |
| $F_{22}$-G-3 | 33,165,306 | 26,488,086 (79.87%) | 29,762,545 (89.74%) |
| $F_{22}$-S-1 | 29,486,446 | 23,377,739 (79.28%) | 26,499,469 (89.87%) |
| $F_{22}$-S-2 | 29,661,775 | 23,527,138 (79.32%) | 26,627,375 (89.77%) |
| $F_{22}$-S-3 | 31,881,830 | 25,107,476 (78.75%) | 28,524,673 (89.47%) |
| $F_{22}$-H-1 | 27,795,264 | 22,326,193 (80.32%) | 25,307,587 (91.05%) |
| $F_{22}$-H-2 | 33,310,101 | 26,770,435 (80.37%) | 30,365,488 (91.16%) |
| $F_{22}$-H-3 | 30,270,442 | 24,124,526 (79.70%) | 27,412,912 (90.56%) |
| $F_{22}$-L-1 | 35,893,465 | 26,442,987 (73.67%) | 32,770,733 (91.30%) |
| $F_{22}$-L-2 | 13,835,197 | 8,330,329 (60.21%) | 11,134,566 (80.48%) |
| $F_{22}$-L-3 | 24,059,868 | 16,464,330 (68.43%) | 20,744,418 (86.22%) |
| $F_{22}$-BA-1 | 36,137,902 | 26,717,581 (72.53%) | 30,453,410 (84.27%) |
| $F_{22}$-BA-2 | 38,718,295 | 29,073,789 (73.74%) | 32,949,269 (85.10%) |
| $F_{22}$-BA-3 | 59,898,328 | 44,382,817 (72.84%) | 50,398,453 (84.14%) |
| Total | 2,296 million | 1,653 million | 1,923 million |

Note: the symbols of “BL”, “G”, “S”, “H”, “L”, and “BA” represent periods of blastula, gastrula, segmentation, and hatching, and liver and barbel tissues, respectively.

Table S34. The silencing of total expression level in goldfish, common carp, $F_1$, and $F_{22}$.

| | Blastula period | | | Gastrula period | | | Segmentation period | | | Hatching period | | |
|---|---|---|---|---|---|---|---|---|---|---|---|---|
| | $F_1$ | $F_{22}$ | Shared | $F_1$ | $F_{22}$ | Shared | $F_1$ | $F_{22}$ | Shared | $F_1$ | $F_{22}$ | Shared |
| CC (silencing)-RCC (expression)-hybrid (expression) | 23 | 34 | 23 | 2 | 4 | 2 | 30 | 25 | 19 | 24 | 16 | 13 |
| CC (silencing)-RCC (expression)-hybrid (silencing) | 3 | 0 | 0 | 1 | 0 | 0 | 5 | 5 | 2 | 1 | 4 | 0 |
| CC (silencing)-RCC (silencing)-hybrid (expression) | 122 | 363 | 81 | 89 | 176 | 34 | 24 | 140 | 13 | 0 | 4 | 0 |
| CC (expression)-RCC (silencing)-hybrid (silencing) | 1,201 | 53 | 47 | 1,490 | 48 | 37 | 250 | 19 | 10 | 141 | 2 | 0 |
| CC (expression)-RCC (silencing)-hybrid (expression) | 238 | 1,914 | 231 | 1,395 | 4,306 | 1,352 | 850 | 1,936 | 816 | 58 | 660 | 51 |
| CC (expression)-RCC (expression)- hybrid (silencing) | 126 | 0 | 0 | 10 | 0 | 0 | 2 | 1 | 0 | 2 | 1 | 0 |

Table S35. The silencing of homoeologs R or C detected in intergeneric $F_1$ and allotetraploid $F_{22}$.

| | | Silencing of homoeolog R (RHS) | Silencing of homoeolog C (CHS) | No .of total expressed genes | Ratio of CHS |
|---|---|---|---|---|---|
| Blastula period | $F_1$ | 1 | 602 | 2,393 | 25.16% |
| | $F_{22}$ | 1 | 10 | 3,566 | 0.28% |
| | Shared | 0 | 10 | / | |
| Gastrula period | $F_1$ | 0 | 114 | 1,238 | 9.21% |
| | $F_{22}$ | 0 | 3 | 1,393 | 0.22% |
| | Shared | 0 | 2 | / | |
| Segmentation period | $F_1$ | 4 | 81 | 3,672 | 2.21% |
| | $F_{22}$ | 1 | 10 | 3,735 | 0.27% |
| | Shared | 0 | 7 | / | |
| Hatching period | $F_1$ | 2 | 75 | 8,441 | 0.89% |
| | $F_{22}$ | 1 | 4 | 9,050 | 0.04% |
| | Shared | 1 | 0 | / | |

Table S36. The distribution of homoeologous expression bias (HEB) based on $log_2$ (R *vs.* C) in intergeneric $F_1$ and the allotetraploid $F_{22}$ of goldfish and common carp.

| | | Potential R HEB[a] | Potential C HEB[a] | R HEB[b] | C HEB[b] |
|---|---|---|---|---|---|
| Blastula period | *In silico* hybrid | 2 | 331 | 42 | 663 |
| | $F_1$ | 37 | 1 | 566 | 139 |
| | $F_{22}$ | 4 | 3 | 402 | 303 |
| Gastrula period | *In silico* hybrid | 3 | 427 | 35 | 727 |
| | $F_1$ | 26 | 1 | 599 | 163 |
| | $F_{22}$ | 13 | 0 | 465 | 297 |
| Segmentation period | *In silico* hybrid | 92 | 276 | 1,025 | 1,785 |
| | $F_1$ | 46 | 7 | 1,922 | 888 |
| | $F_{22}$ | 29 | 8 | 1,584 | 1,226 |
| Hatching period | *In silico* hybrid | 43 | 417 | 1,647 | 4,160 |
| | $F_1$ | 56 | 9 | 3,496 | 2,311 |
| | $F_{22}$ | 33 | 7 | 3,571 | 2,236 |
| Liver | *In silico* hybrid | 72 | 46 | 1,690 | 1,349 |
| | $F_1$ | 40 | 33 | 1,715 | 1,324 |
| | $F_{22}$ | 28 | 2 | 1,730 | 1,308 |
| Barbel | *In silico* hybrid | 251 | 49 | 6,156 | 3,551 |
| | $F_1$ | 79 | 26 | 5,701 | 4,006 |
| | $F_{22}$ | 90 | 32 | 5,659 | 4,048 |

Note: [a]The value of $log_2$ (R *vs.* C) < 1 and > 0 were considered as potential R HEB in hybrids. Conversely, it represents as potential C HEB.

[b]The value of $log_2$ (R *vs.* C) > 1 was considered as R HEB in hybrids. Conversely, it represents as C HEB.

## Supplementary Figure

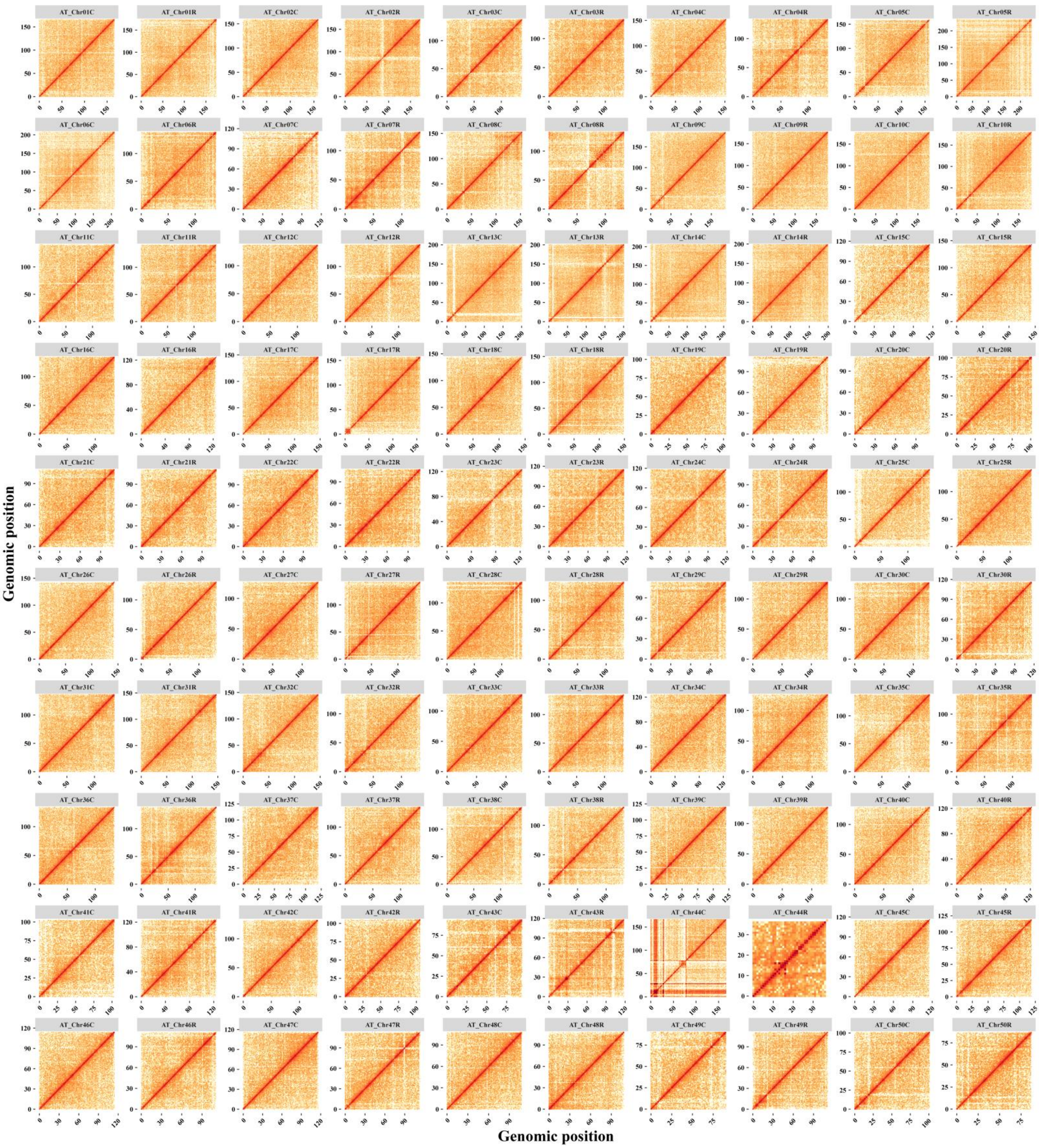


Fig. S1. Heatmap of the allotetraploid constructed by distance of the interactions within and among chromosomes according to Hi-C analyses. Chromosomes predicted by Lachesis were cut into bins of an equal length of 200 kb and a heatmap was constructed based on the interaction signals that were revealed by valid mapped read pairs between bins.

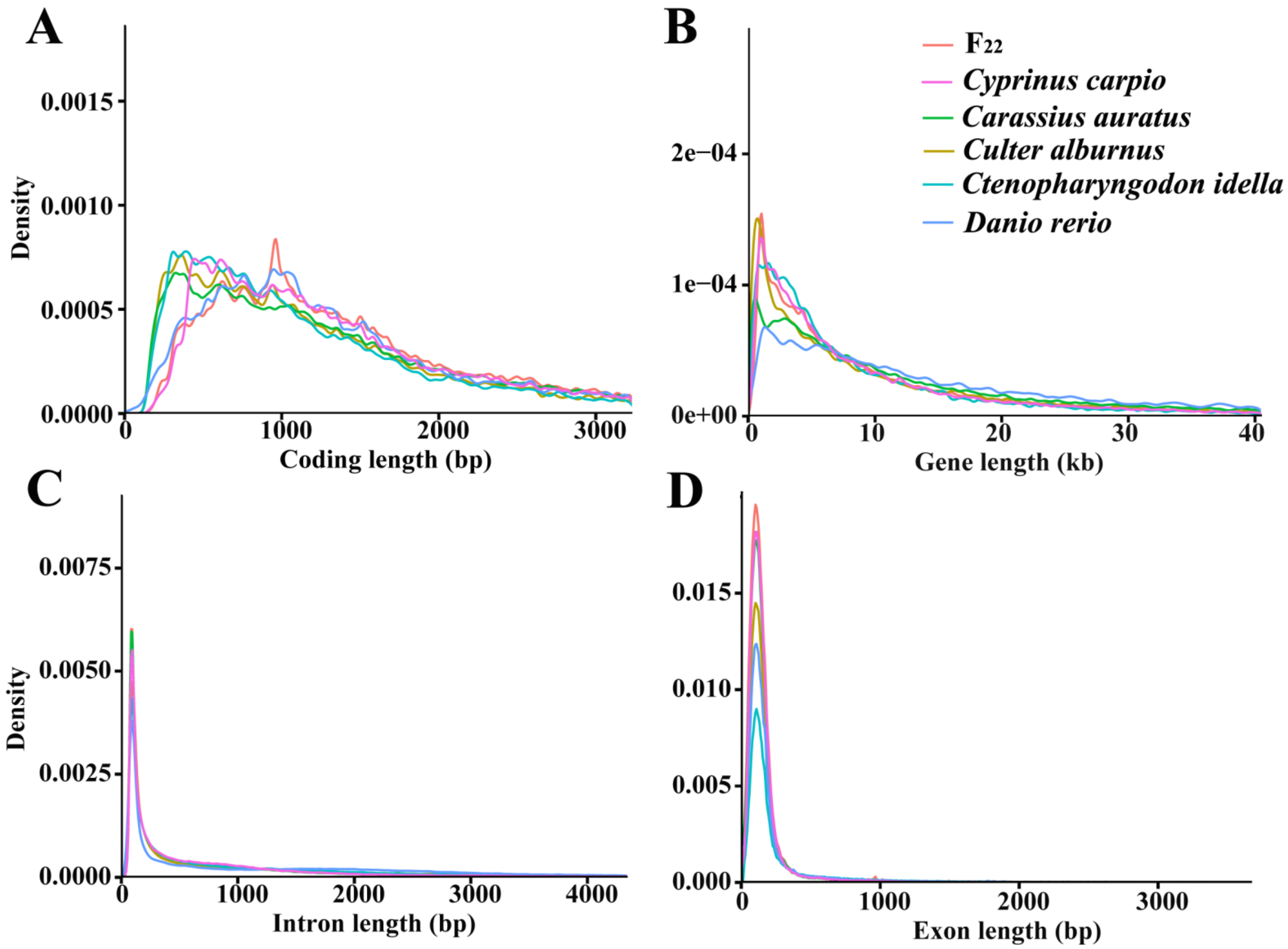


Fig. S2. The lengths of annotated genes in the allotetraploid, *C. carpio*, *C. auratus*, *Culter alburnus*, *Danio rerio*, and *Ctenopharyngodon idella*. (A) Length of coding sequences in each gene was obtained from these species and the allotetraploid $F_{22}$. (B) Length of gene sequences in each gene. (C). Length of intron sequences in each gene. (D) Length of each exon.

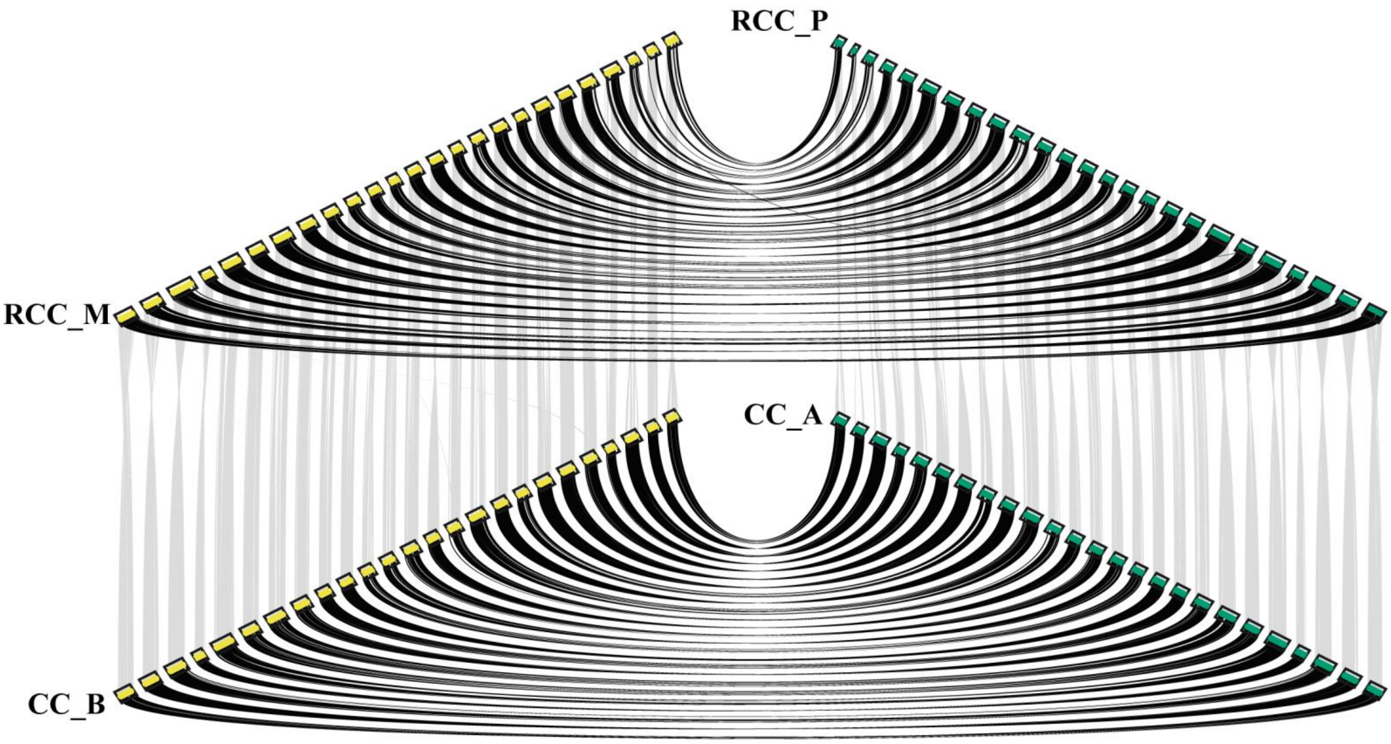


Fig. S3. Genome synteny of the two subgenomes in goldfish and common carp. Block represents the assembled chromosome. The subgenome M in goldfish and subgenome B in common carp (yellow river carp) were derived from a common maternal ancestor (yellow), while subgenome P in goldfish and subgenome A in common carp were derived from a common paternal ancestor (green). The grey line represents the homologous gene pairs (OGPs) between goldfish and common carp, while black line represents the paralogous gene pairs between the two subgenomes.

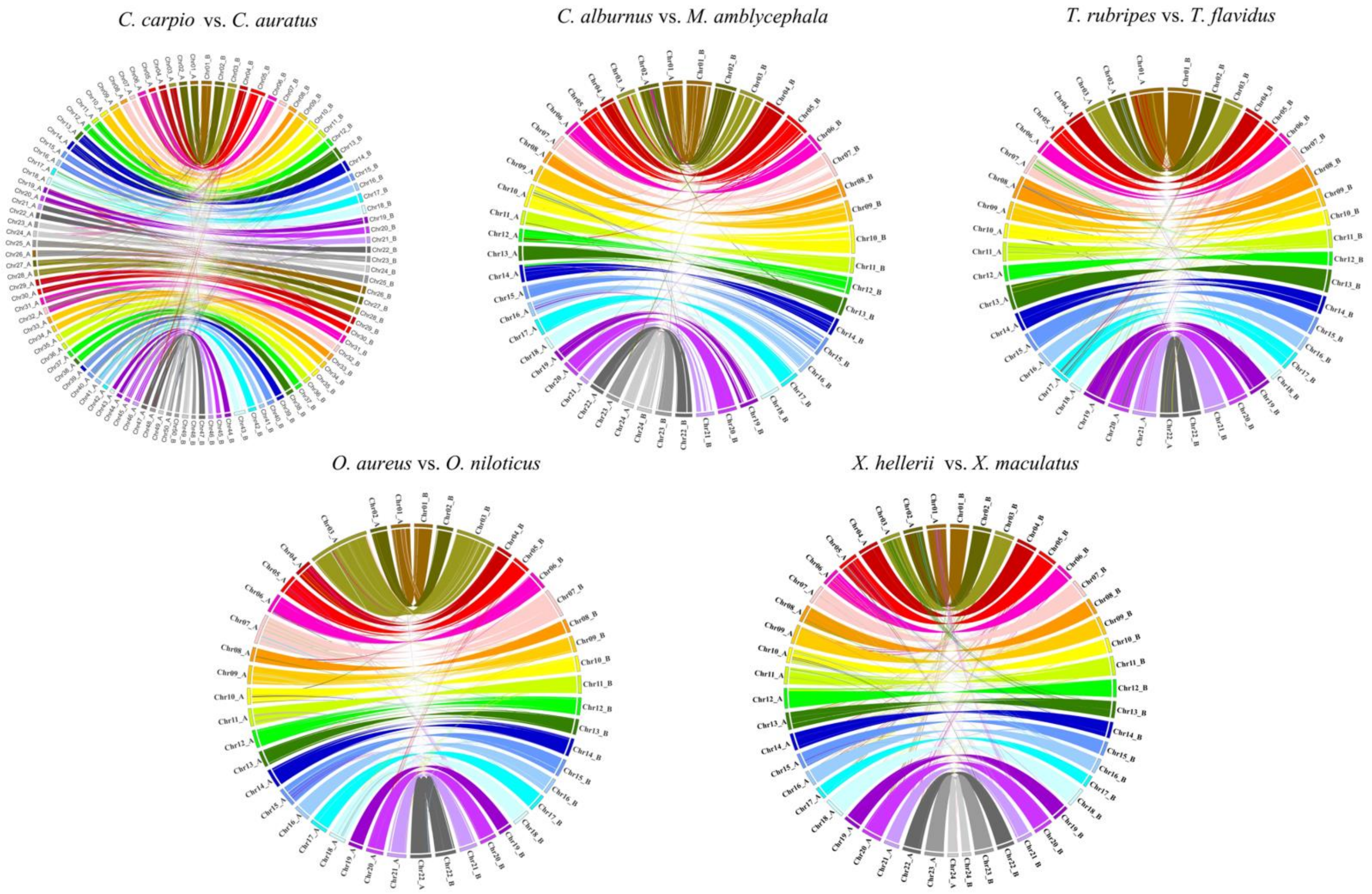


Fig. S4. The genome synteny in two parents of the five hybrid groups, including the hybrid group of *C. carpio* (A, 2n = 100) *vs. C. auratus* (B, 2n = 100), the hybrid group of *C. alburnus* (A, 2n = 48) *vs. M. amblycephala* (B, 2n = 48), the hybrid group of *T. rubripes* (A, 2n = 44) *vs. T. flavidus* (B, 2n = 44), the hybrid group of *O. aureus* (A, 2n = 44) *vs. O. niloticus* (B, 2n = 44), the hybrid group of *X. hellerii* (A, 2n = 48) *vs. X. maculatus* (B, 2n = 48). Colored lines indicate the orthologous sites of gene blocks and their colinear relationships between genomes A and B. The numbers in order were based on the collinearity relationships with the zebrafish genome.

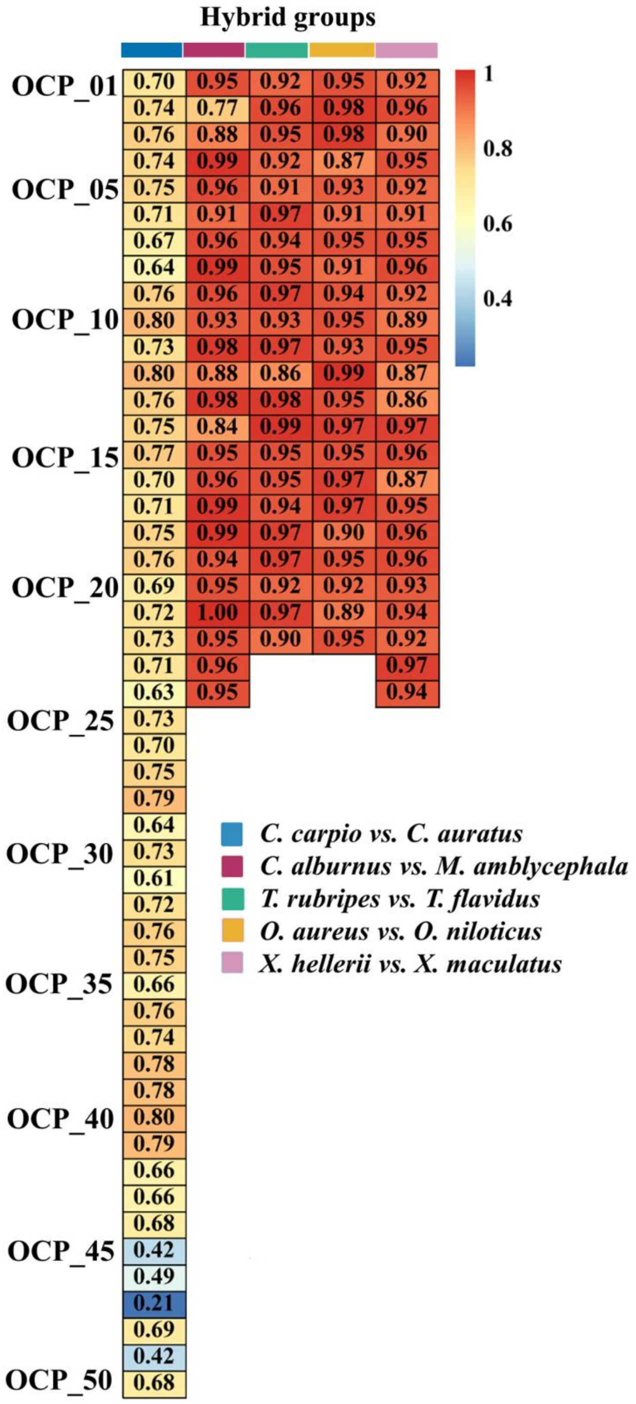


Fig. S5. Analysis of orthologous genes in the two parental genomes of five hybrid lineages. The rate represents the orthologous gene markers with clear origins of the ancestral parents determined by orthologous gene analysis. Chromosome numbers are ordered according to the collinearity relationships with the zebrafish genome. OCP: orthologous chromosome pair.

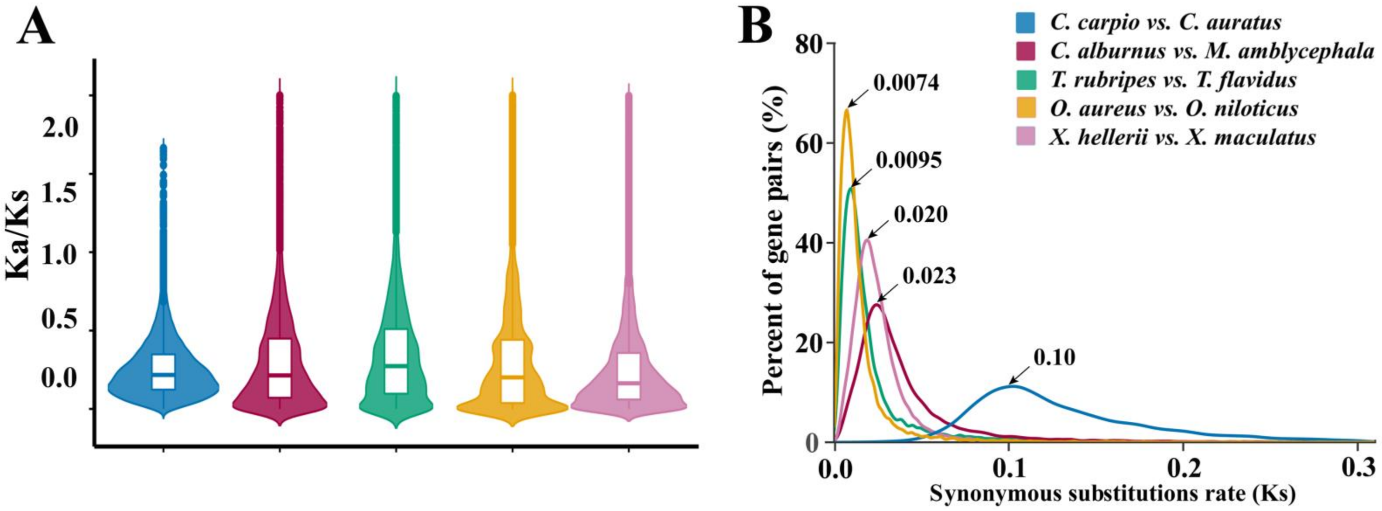


Fig. S6. Analysis of $K_a/K_s$ and $K_s$ values in the two parental genomes of five hybrid lineages. (A) The distribution of $K_a/K_s$ values among OGPs. (B) The distribution of $K_s$ value among OGPs.

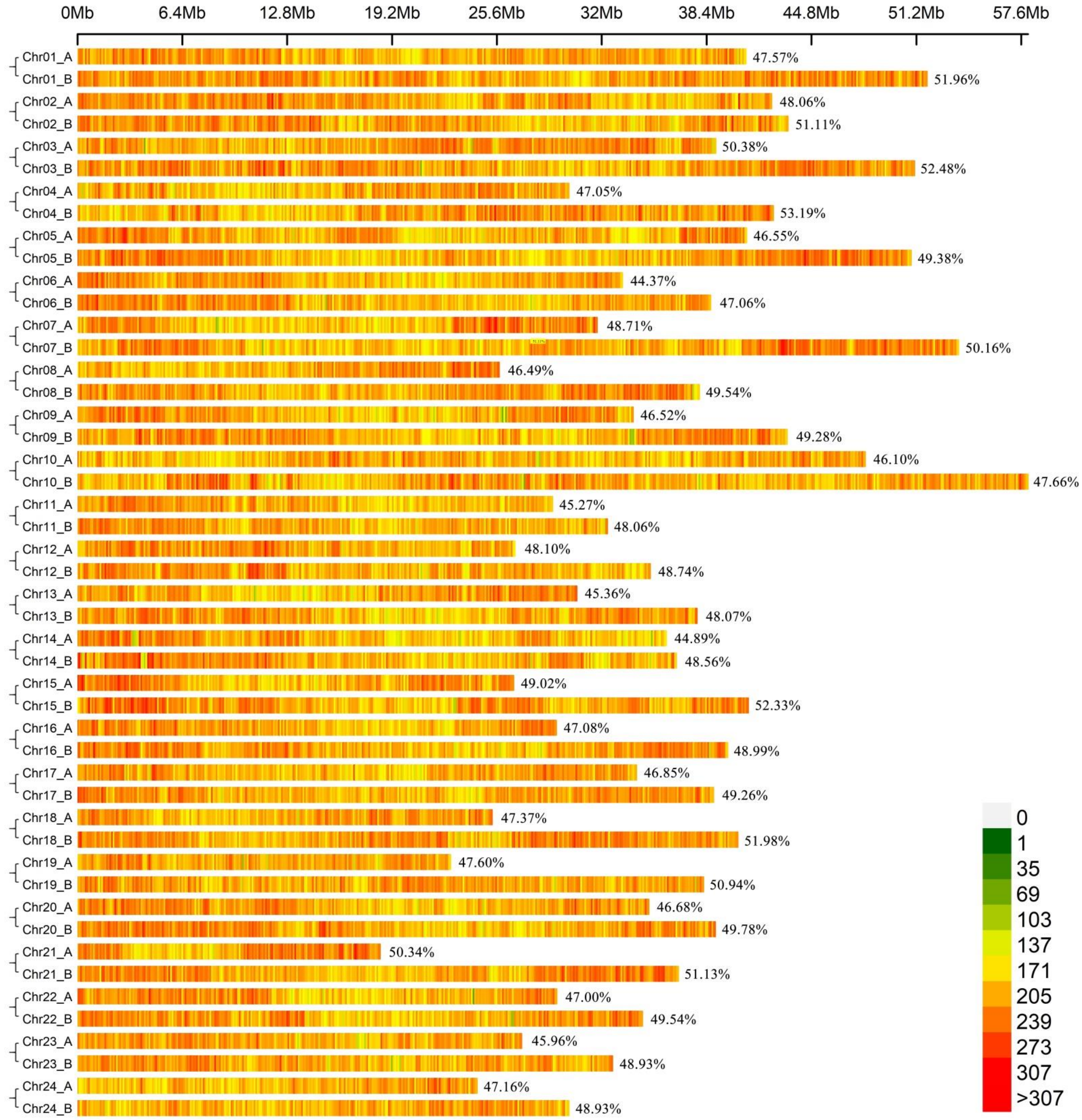


Fig. S7. The density of TEs in the OCPs of *C. alburnus* (A) *vs. M. amblycephalavs* (B).

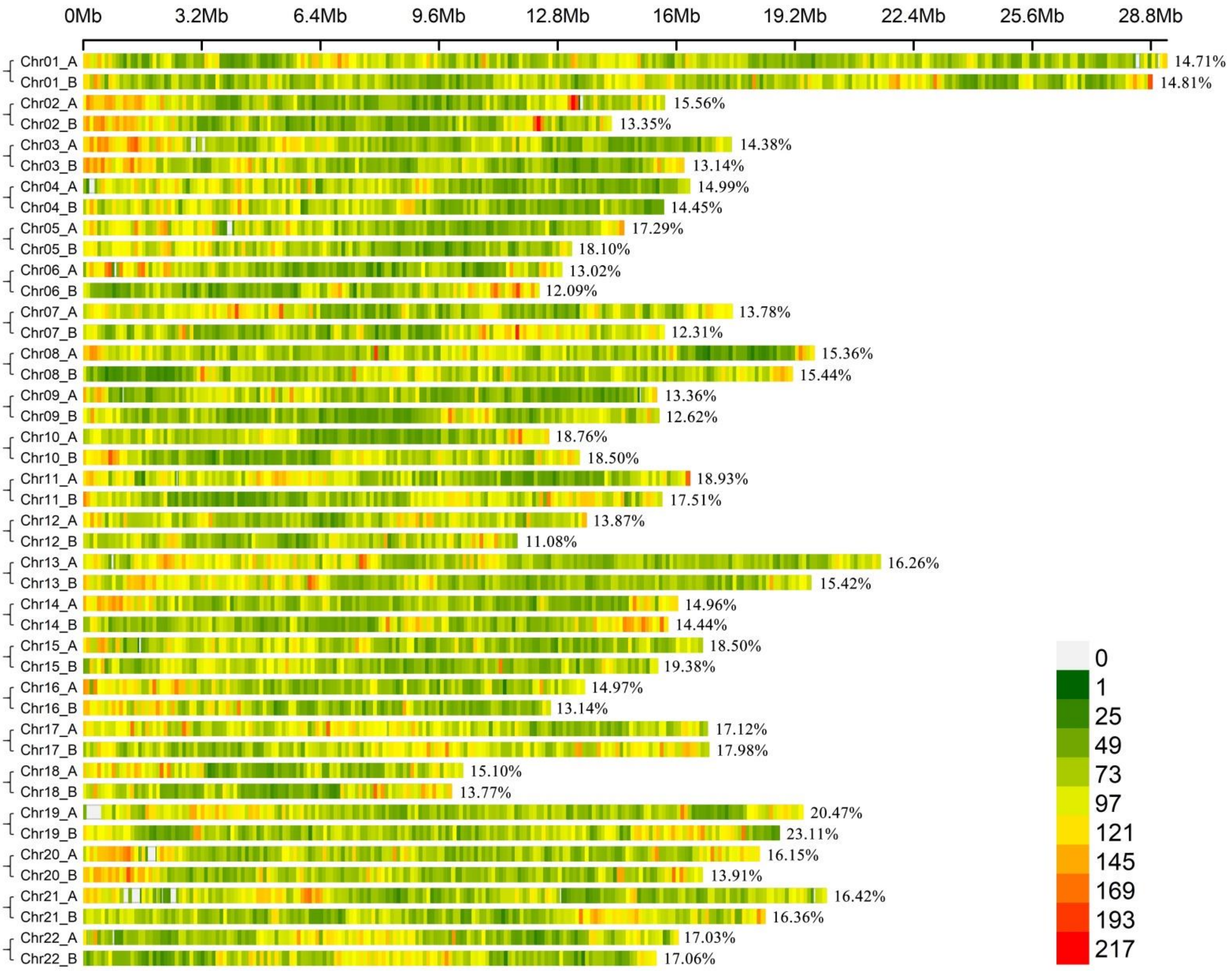


Fig. S8. The density of TEs in the OCPs of *T. rubripes* (A) *vs. T. flavidus* (B).

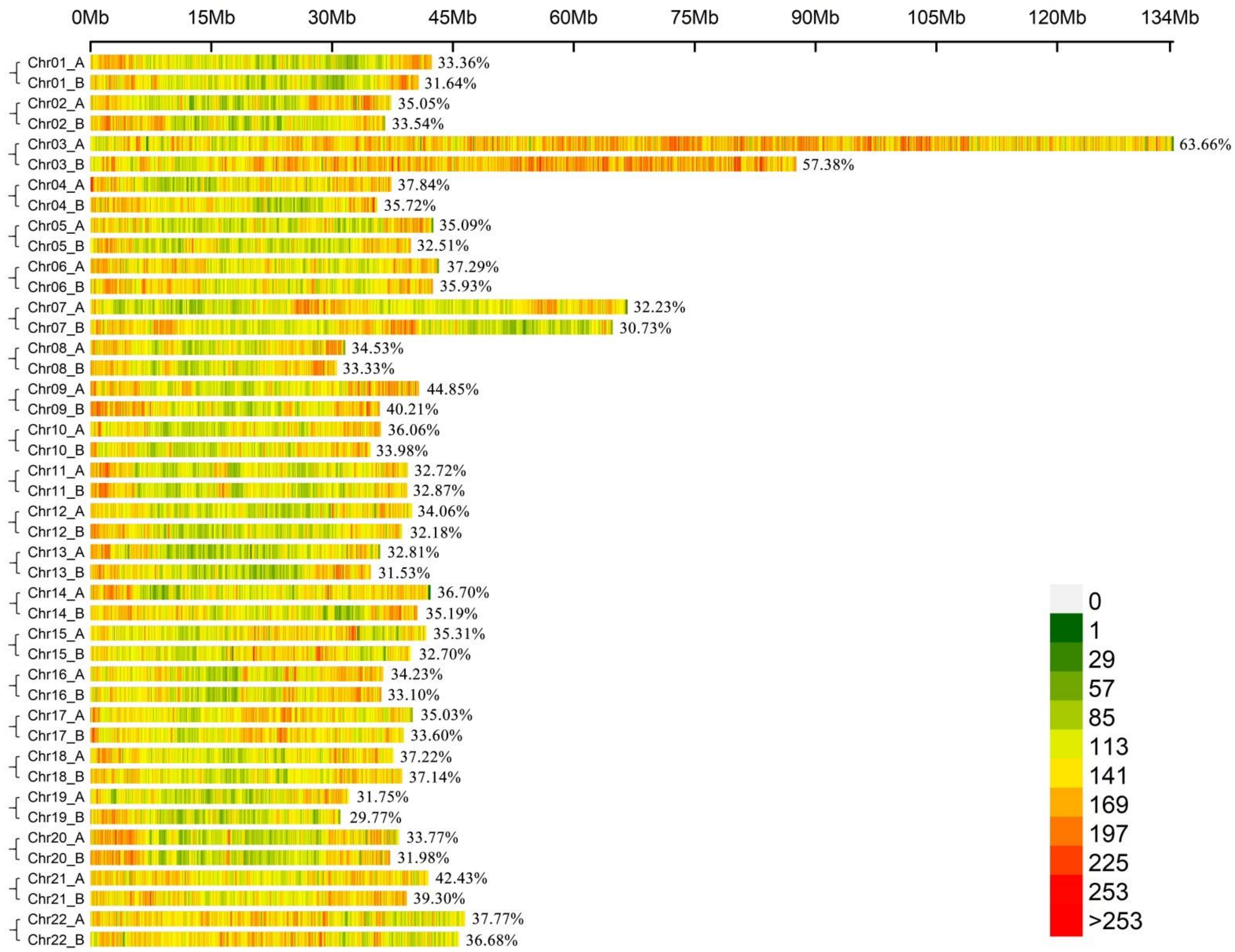


Fig. S9. The density of TEs in the OCPs of *O. aureus* (A) *vs. O. niloticus* (B).

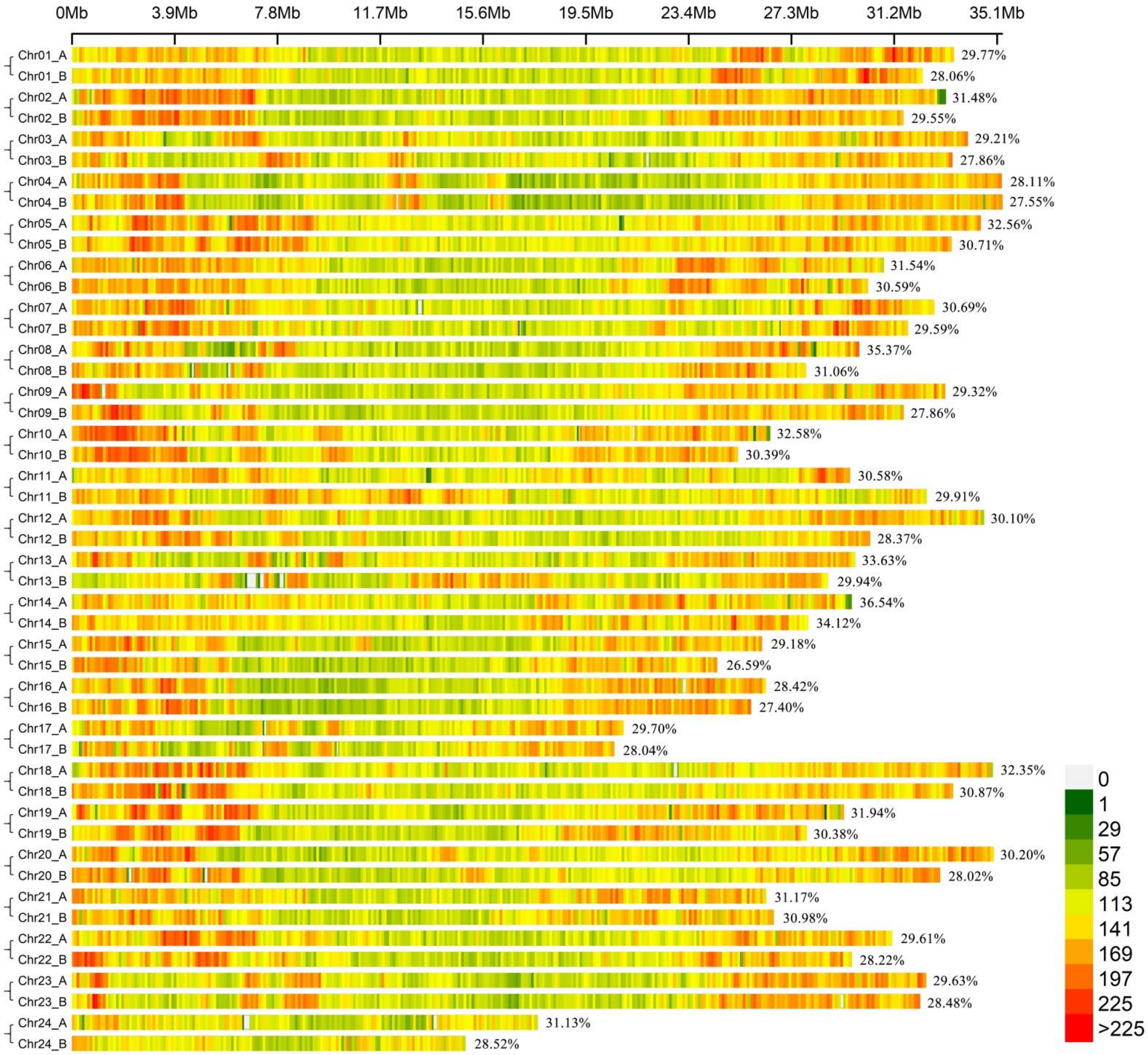


Fig. S10. The density of TEs in the OCPs of *X. hellerii* (A) *vs. X. maculatus* (B).

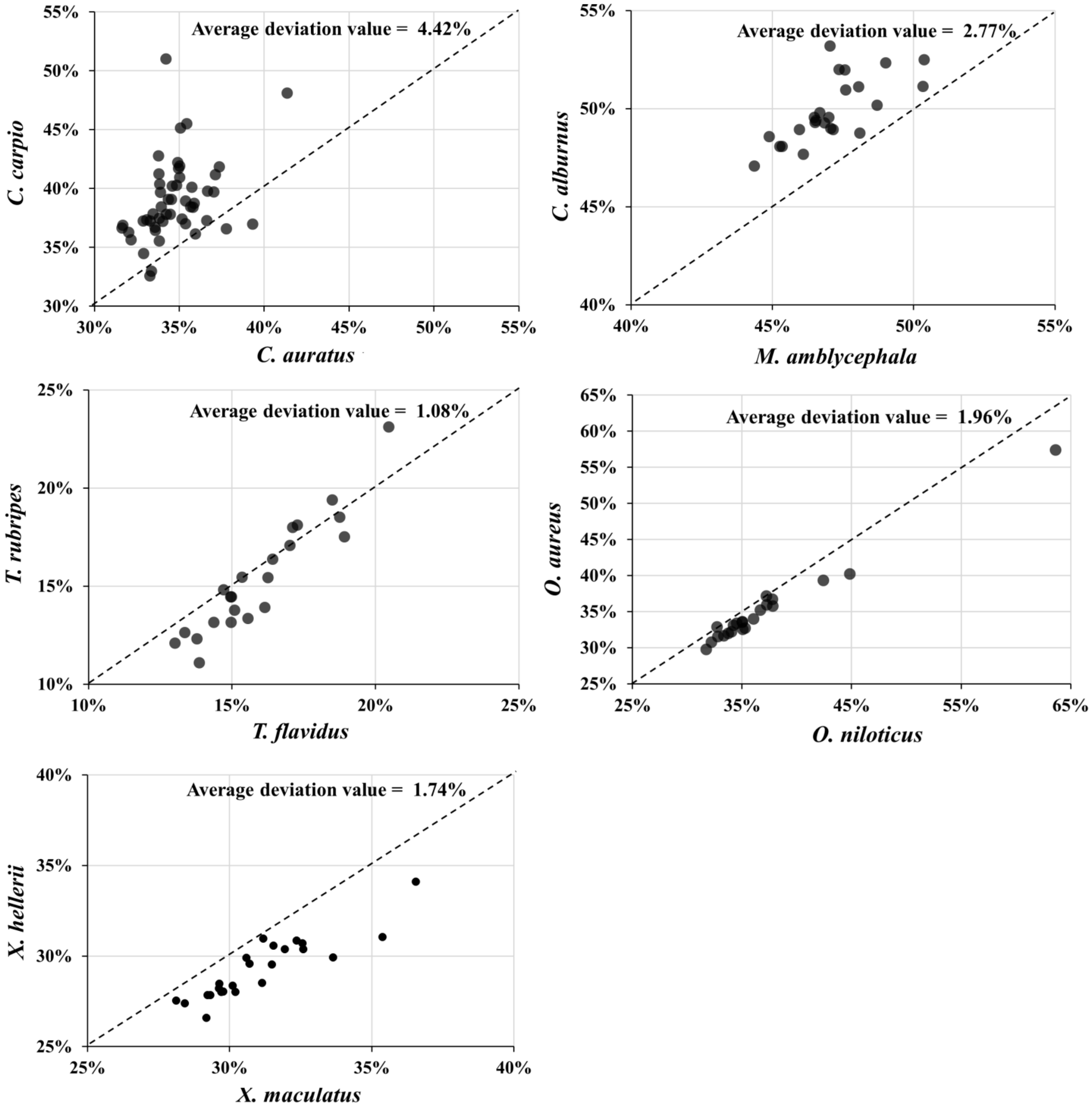


Fig. S11. The distribution of TE rates in OCPs. The average deviation value reflects the TE differences between the two parents of the five hybrid groups (*C. carpio vs. C. auratus, C. alburnus vs. M. amblycephala, T. rubripes vs. T. flavidus, O. aureus vs. O. niloticus,* and *X. hellerii vs. X. maculatus*).

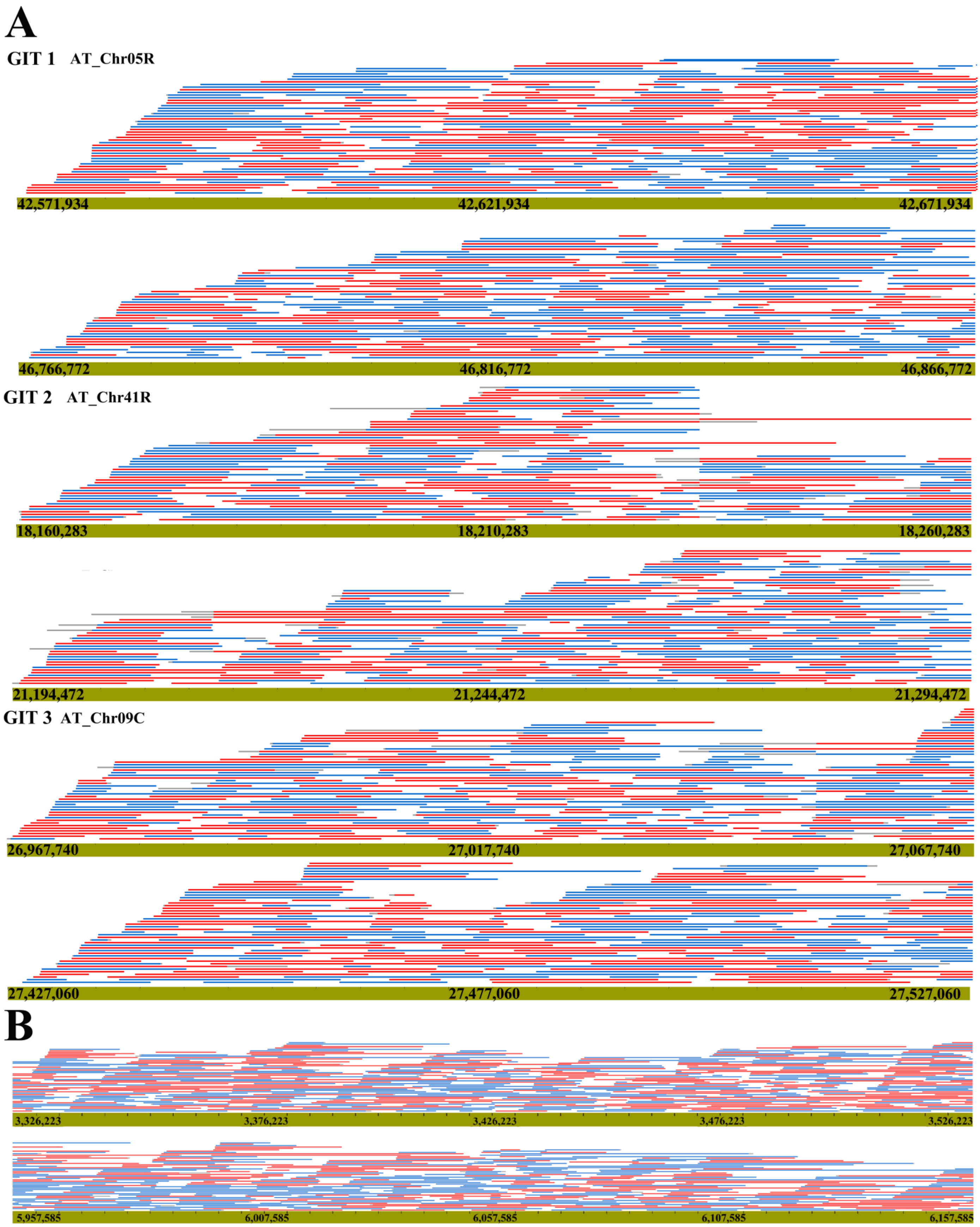


Fig. S12. Genomic variation checked in genome assembly. (A) The three gene interchromosomal translocations (GITs) of the allotetraploid were checked using the mapped PacBio reads (red and blue lines represent forward and reverse reads, respectively). (B) Unequal HR in AT_chr39 (3,426,224-6,057,586 bp) HCP. The mapped PacBio reads (red and

blue lines represent forward and reverse reads, respectively) in the allotetraploid ($F_{22}$_1) confirmed no assembly error in the breakpoints of syntenic region (black arrow).

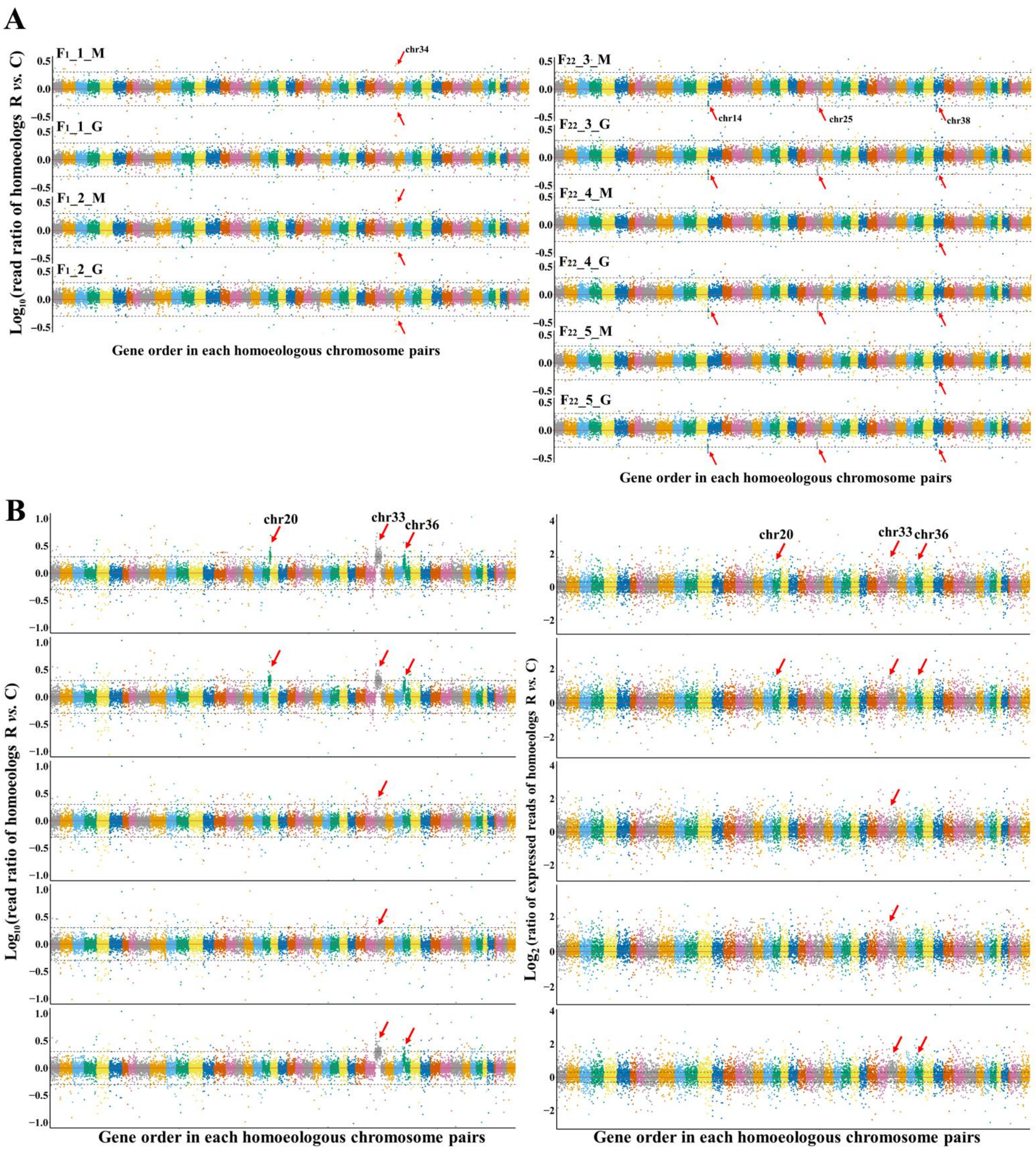


Fig. S13. Determination of unequal HR in the intergeneric $F_1$ and allotetraploid $F_{24}$. (A) Ratio of gene copy numbers of R *vs*. C homoeologs in muscle and gonad tissues of the intergeneric $F_1$ and allotetraploids $F_{22}$ and $F_{24}$. The red solid lines represent the *in silico* prediction of the ratio of R *vs*. C homoeologs ($Log_{10}(1) = 0$). The black dashed lines represent the threshold values of R *vs*. C homoeologs ($Log_{10}(0.5) = -0.30103$ and $Log_{10}(2) = 0.30103$. The dot represent the ratios of R *vs*. C homoeologs ($Log_{10}(x)$) obtained from the numbers of mapped reads of R and C

homoeologs in HCPs (different colours) for coding regions. “M” and “G” represent muscle and gonad tissues, respectively. (B) Ratios of gene copy numbers of R *vs*. C homoeologs and homoeologous expression bias (HEB) in the caudal fin tissue of five allotetraploid individuals ($F_{24}$).

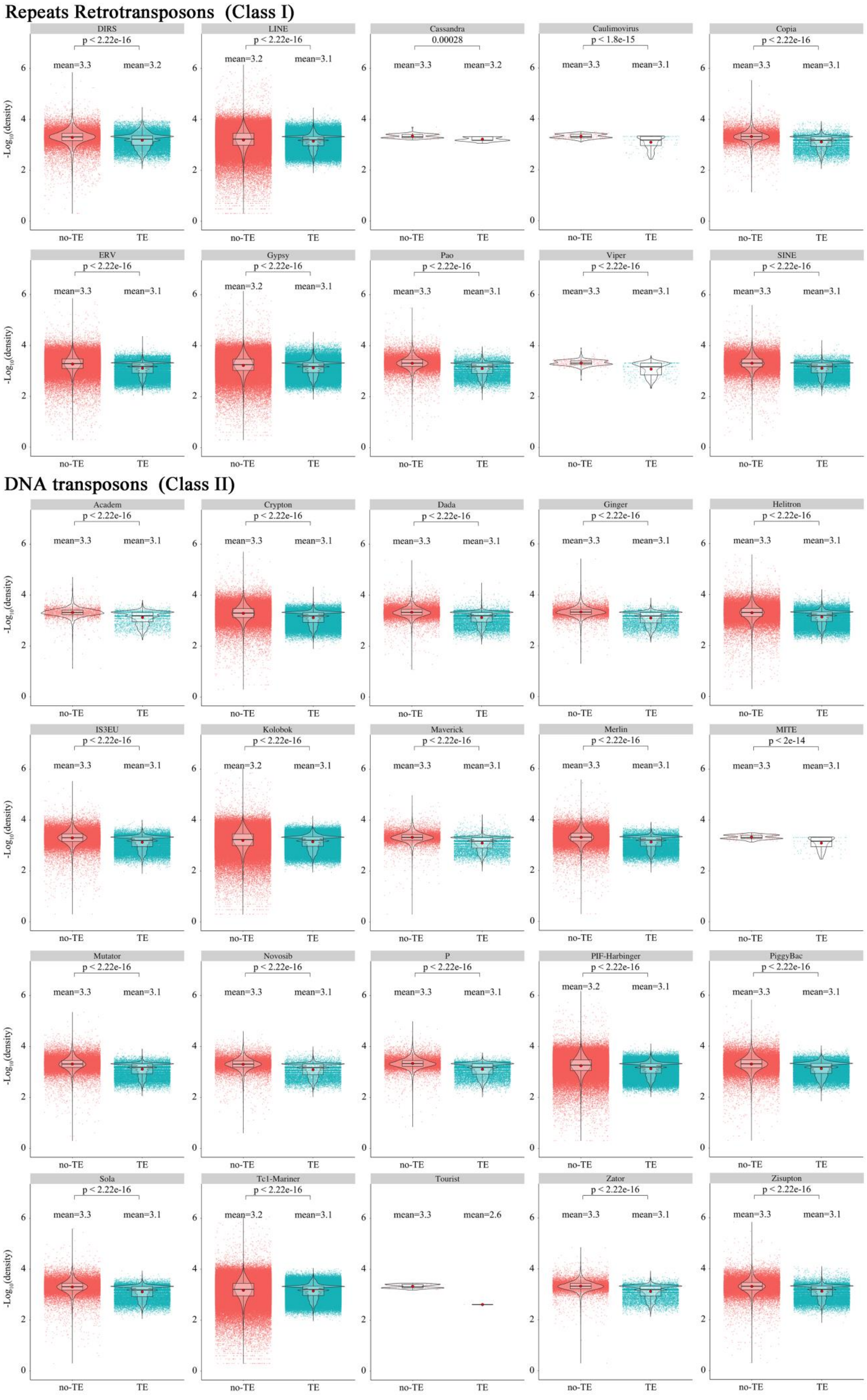


Fig. S14. Correlation relationship between the distribution of TEs and structural variations in each TE type (*t*-test).

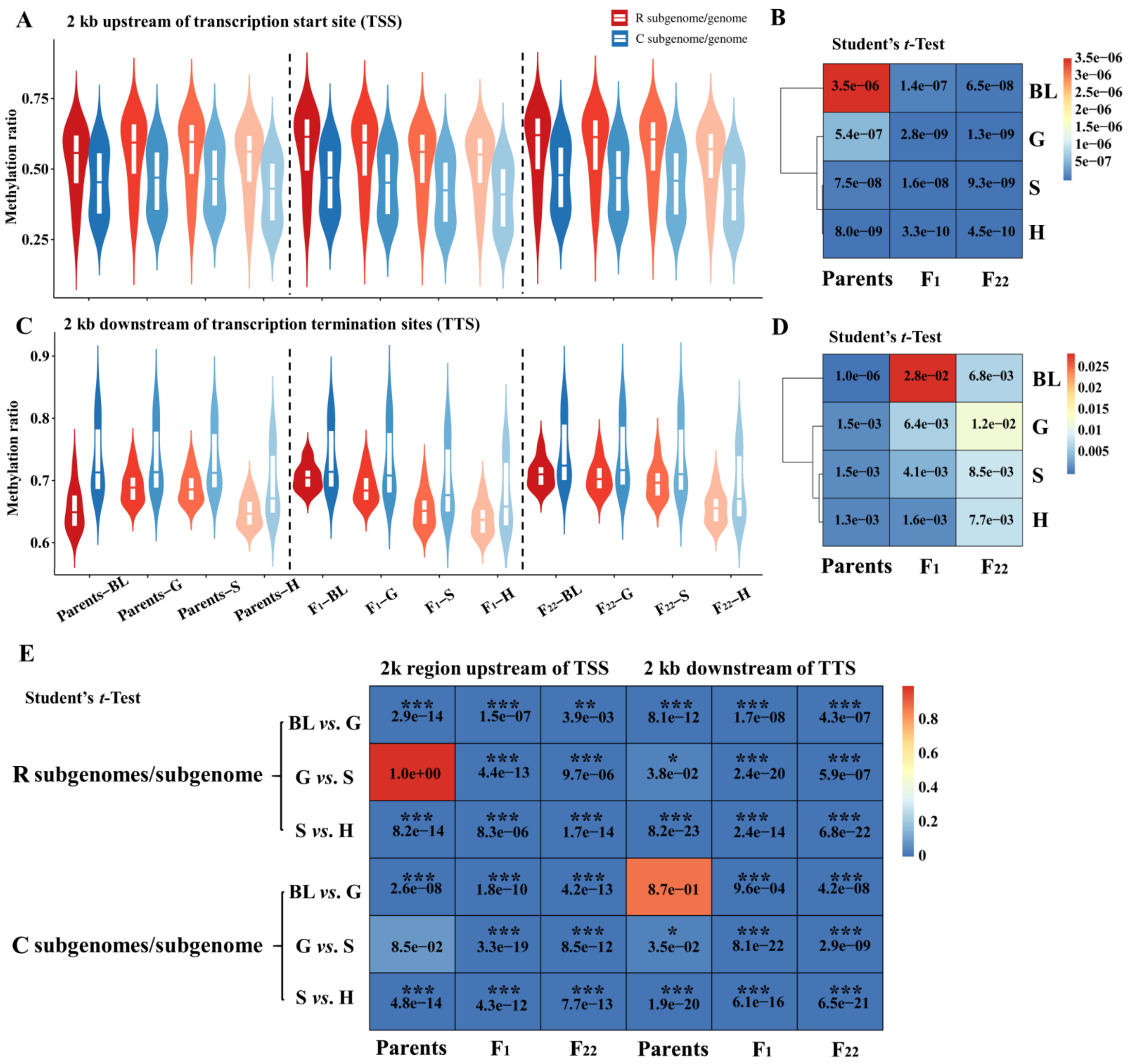


Fig. S15. Methylation levels (MLs) of homoeologs R and C of TSS and TTS in the four development stages of goldfish, common carp, $F_1$, and $F_{22}$. (A) DNA methylation in 2 kb upstream of TSS. (B) Difference analyses between MLs of homoeologs R and C in 2 kb upstream of TSS. (C) DNA methylation in 2 kb downstream of TTS. (D) Difference analyses between MLs of homoeologs R and C in 2 kb downstream of TTS. (E) Difference analyses between two adjacent embryonic developmental periods. The symbols "BL", "G", "S", and "H" represent periods of blastula, gastrula, segmentation, and hatching, respectively.

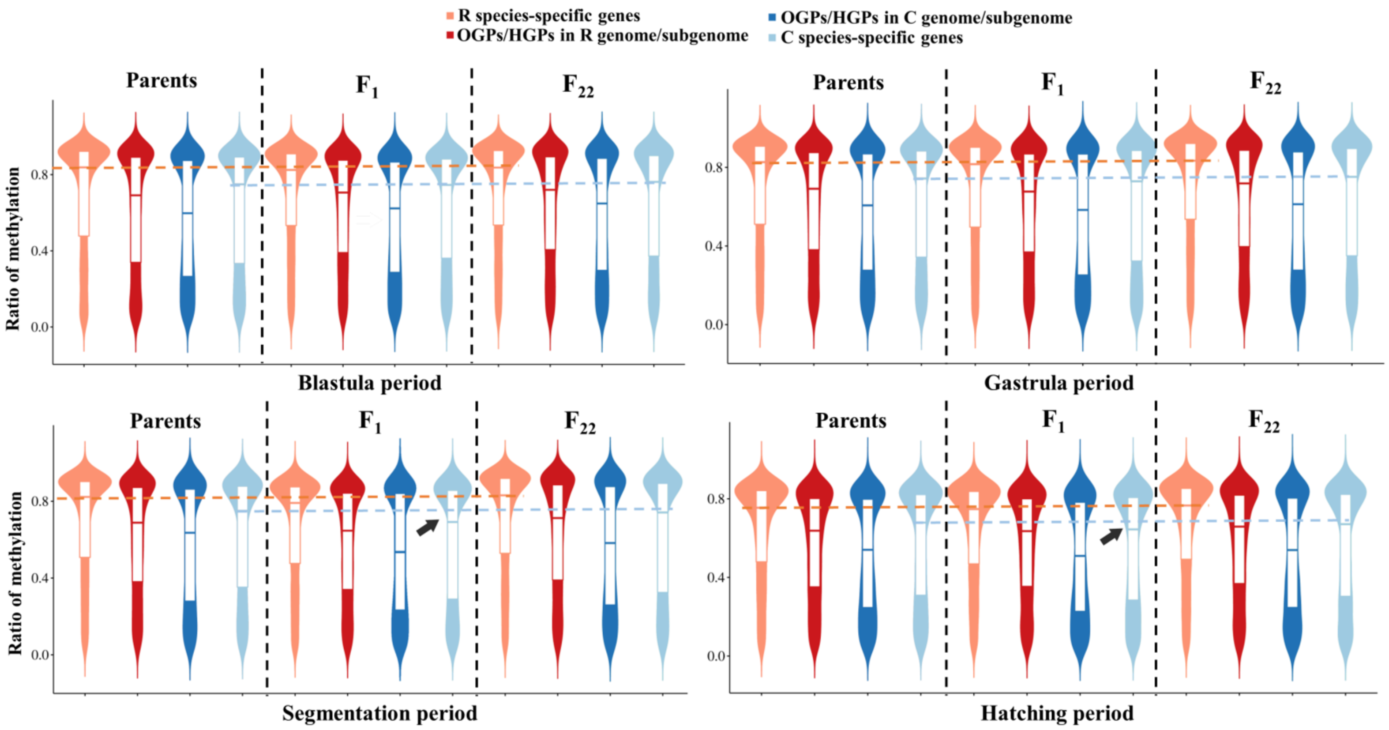


Fig. S16. The distribution of MLs (2k upstream of TSS) in orthologous gene pairs (OGPs) or HGPs, R or C species-specific genes (SSGs), of goldfish, common carp, $F_1$, and $F_{22}$, respectively. Dotted line represents the corresponding average values of methylation rate of the R (orange) or C (light blue) SSGs in the two inbred parents (goldfish and common carp).

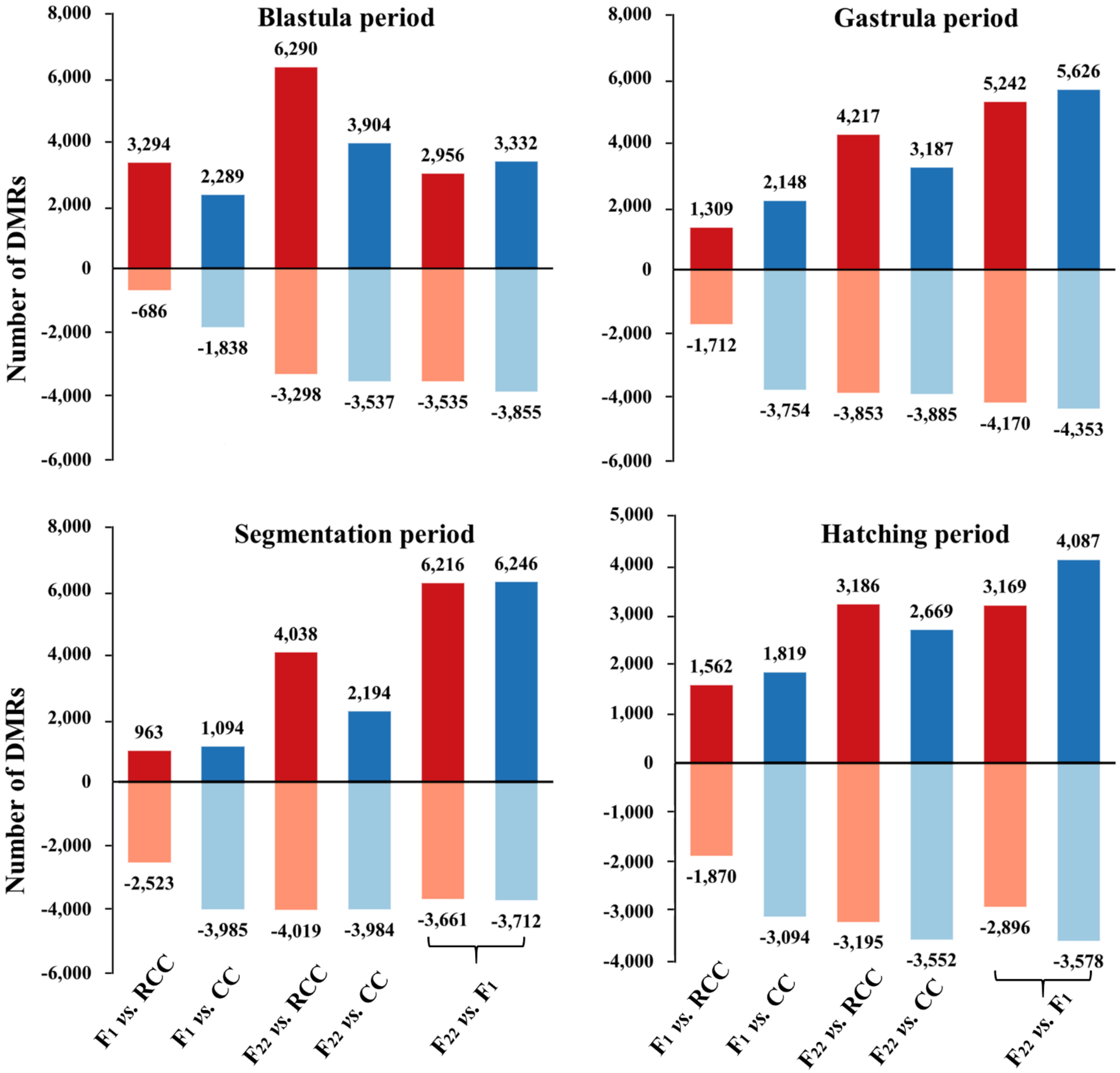


Fig. S17. Differentially methylated regions (DMRs) of the four development stages in subgenomes R and C. In subgenome R, dark red represents higher ML in the former, while light red represents higher ML in the latter. In subgenome C, dark blue represents higher ML in the former, while light blue represents higher ML in the latter.

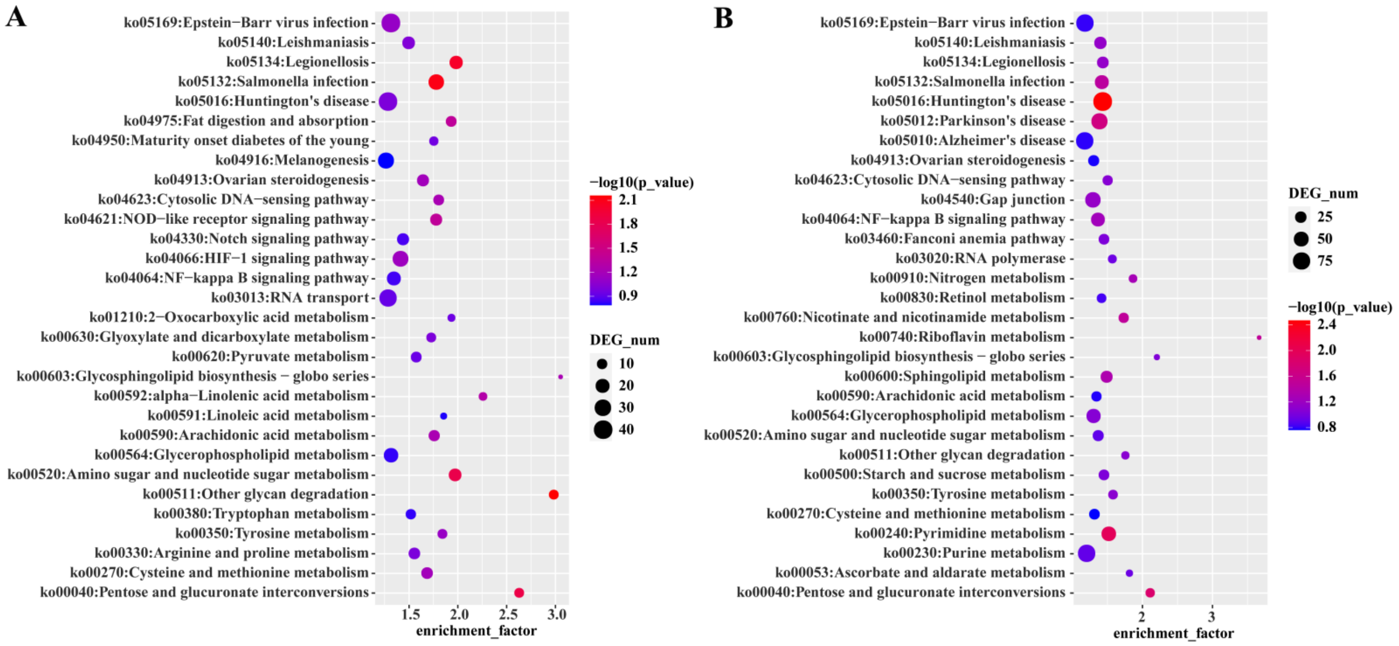


Fig. S18. The top 20 pathways of the DMGs led by polyploidization and transgenerational inheritance. (A) The DNA methylation changes in these DMGs induced by hybridization and recovered to the state of the inbred parents by polyploidization and transgenerational inheritance. (B) The novel DNA methylation changes in these DMGs induced by polyploidization and transgenerational inheritance in the allotetraploid lineage.

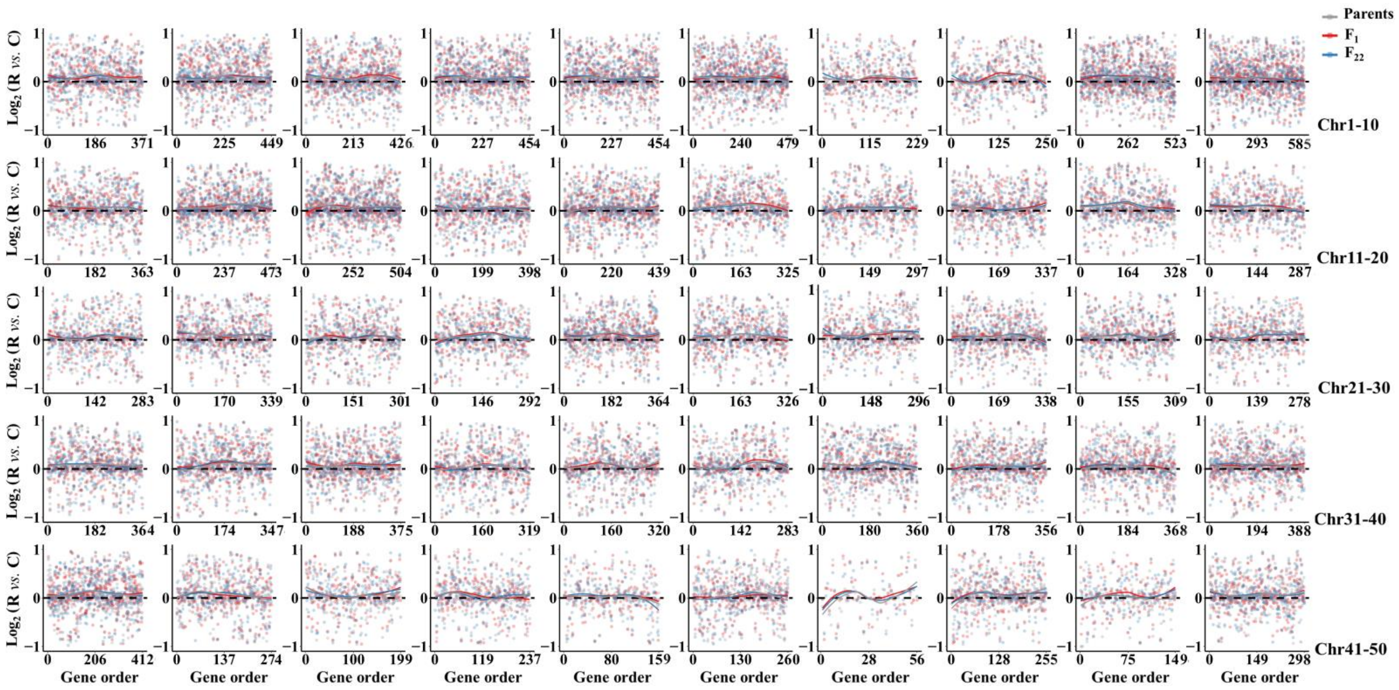


Fig. S19. The distribution of MLs in the blastula period. The difference values were detected based on the difference in MLs of homoeologs R and C in the inbred parents, $F_1$, and $F_{22}$.

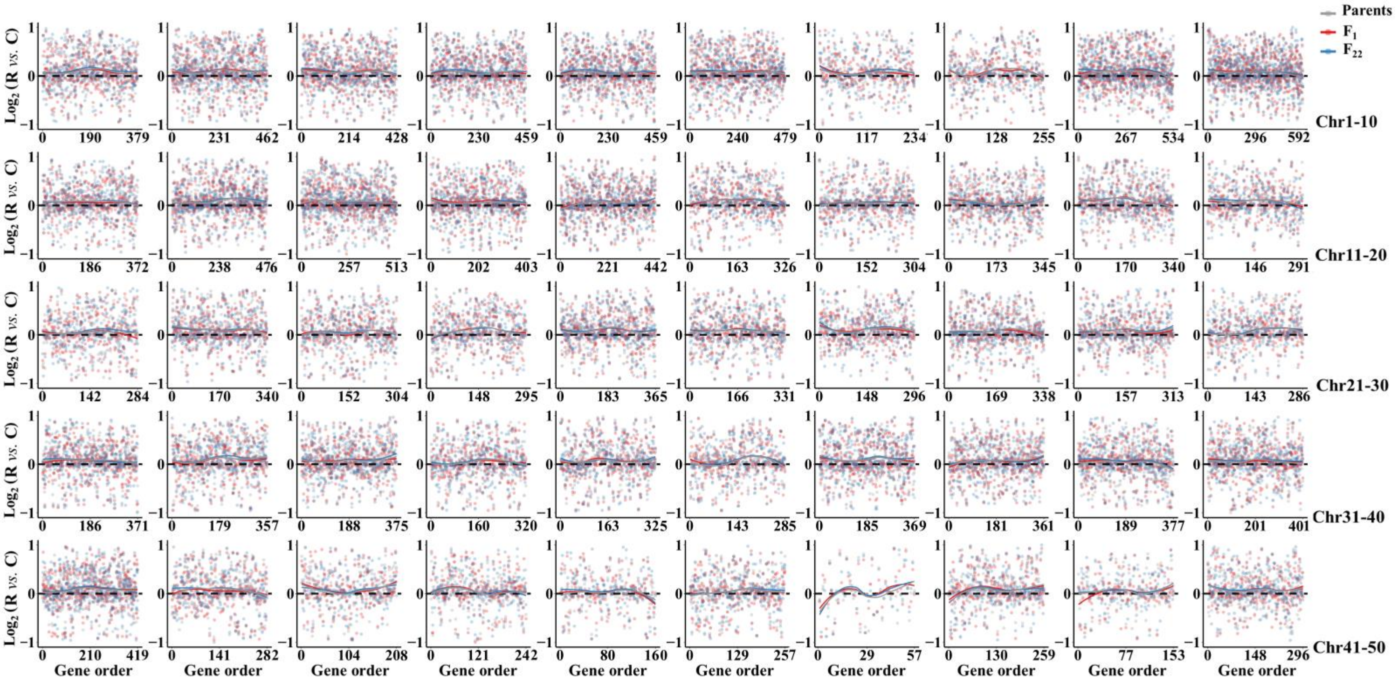


Fig. S20. The distribution of MLs in the gastrula period. The difference values were detected based on the difference in MLs of homoeologs R and C in the inbred parents, $F_1$, and $F_{22}$.

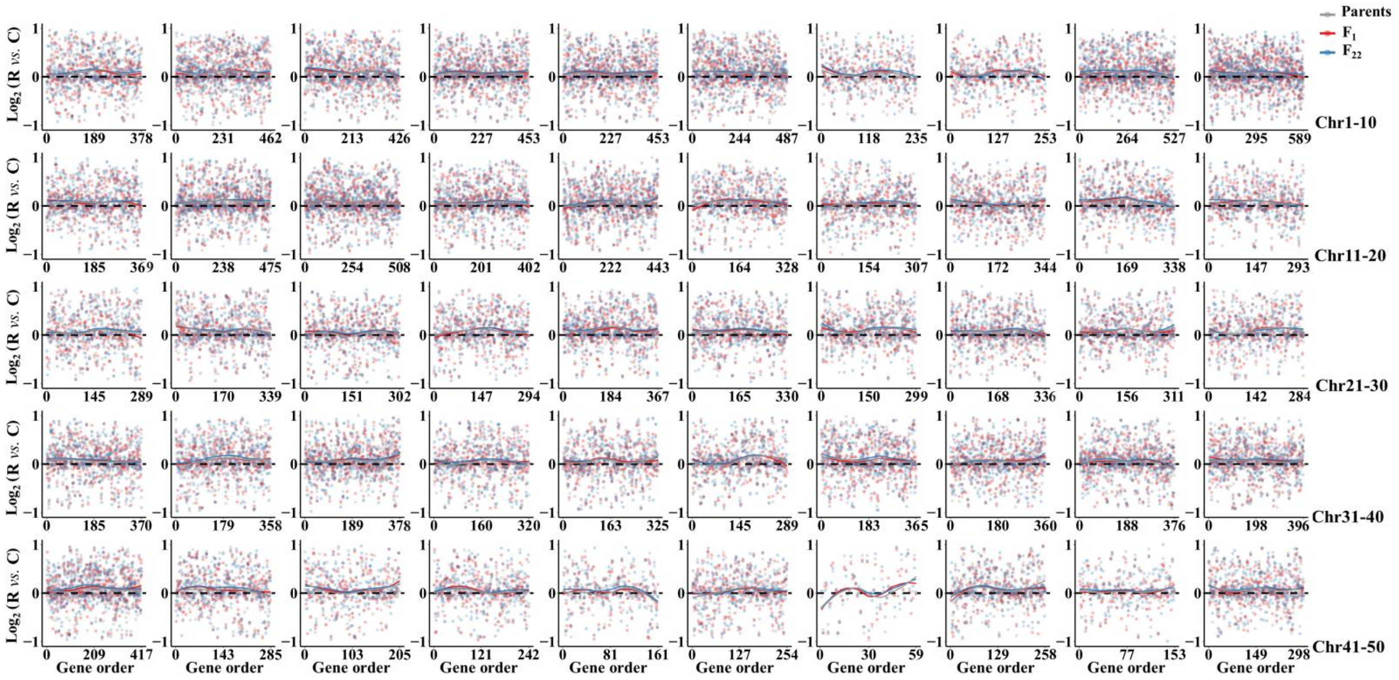


Fig. S21. The distribution of MLs in the segmentation period. The difference values were detected based on the difference in MLs of homoeologs R and C in the inbred parents, $F_1$, and $F_{22}$.

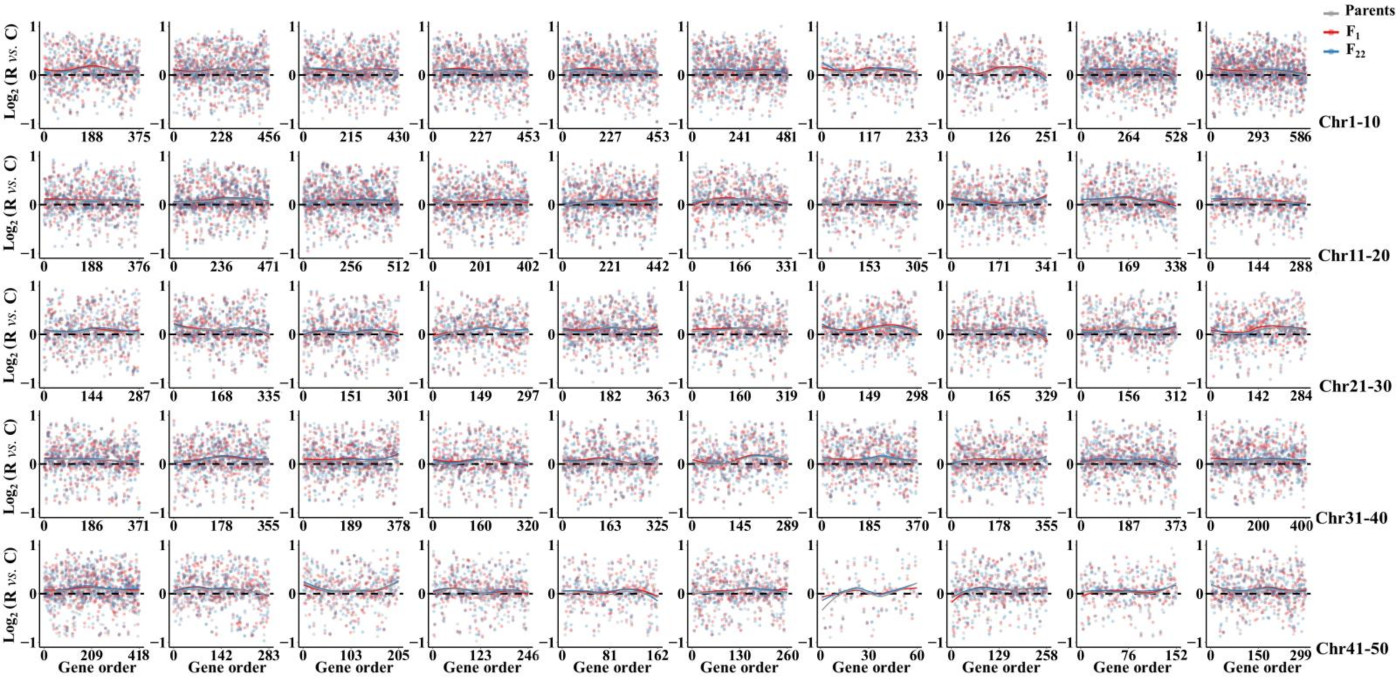


Fig. S22. The distribution of MLs in the hatching period. The difference values were detected based on the difference in MLs of homoeologs R and C in the inbred parents, $F_1$, and $F_{22}$.

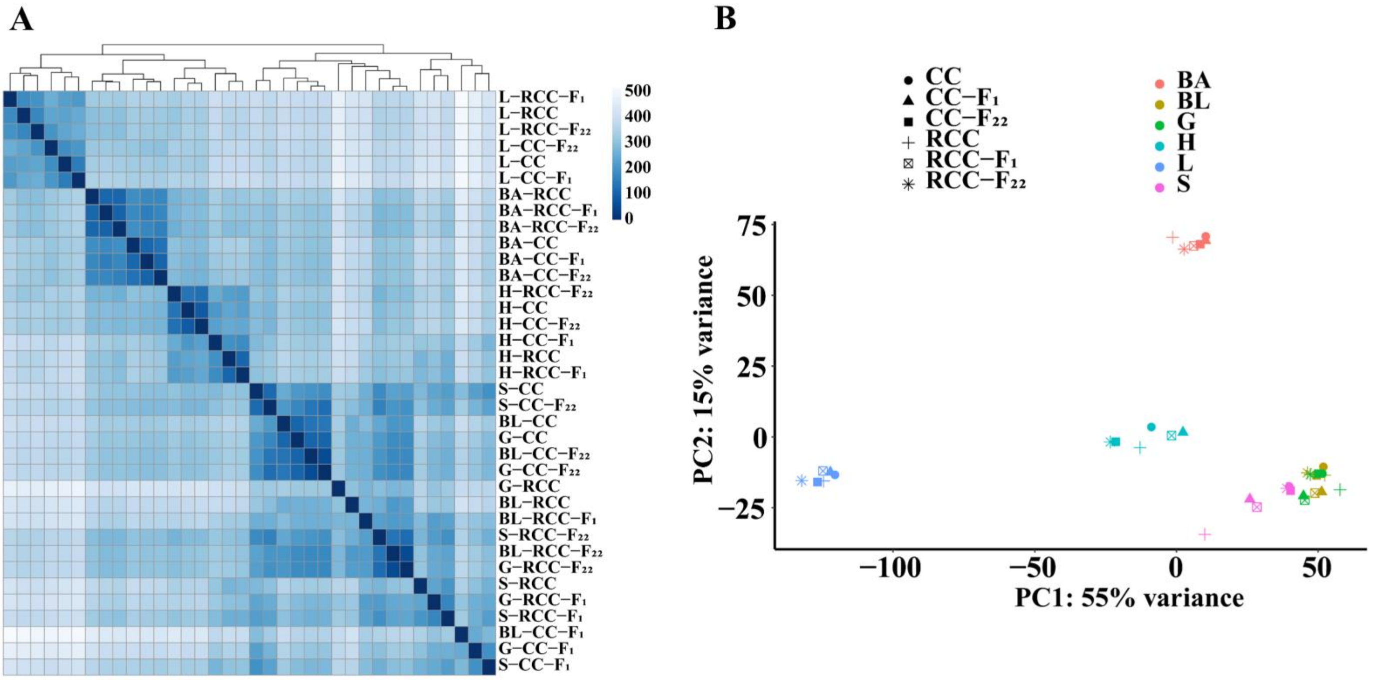


Fig. S23. PCA and cluster analyses performed on expression in $F_1$, $F_{22}$, and their inbred parents. (A) PCA analyses. (B) Cluster analyses. Analyses were performed using the Euclidean distance between the 72 samples, which included expression values of homoeologs R and C in $F_1$ and $F_{22}$, and expression values in their inbred parents (goldfish and common carp). The symbols “BL”, “G”, “S”, “H”, “L”, and “BA” represent periods of blastula, gastrula, segmentation and hatching, and liver and barbel tissues, respectively.

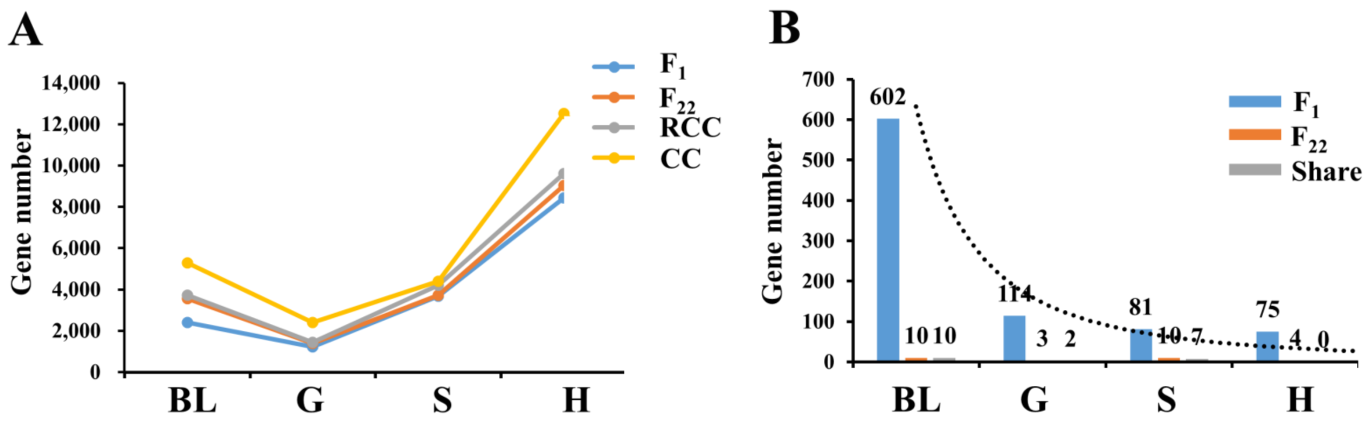


Fig. S24. The distribution of expressed genes and silencing of homoeolog C (CHS) genes in embryonic development of the four fishes. (A) Maternal-to-zygotic transition was observed in embryonic development of goldfish, common carp and the two hybrids. Meanwhile, a decreasing number of expressed genes from BL to G and an increasing number of expressed genes from G to H were affected by zygotic genome activation and the elimination of maternal transcripts. (B) An obvious difference was observed between the gene numbers of CHS of $F_1$ and $F_{22}$ in different embryonic development stages. The symbols “BL”, “G”, “S”, and “H” represent periods of blastula, gastrula, segmentation, and hatching, respectively.

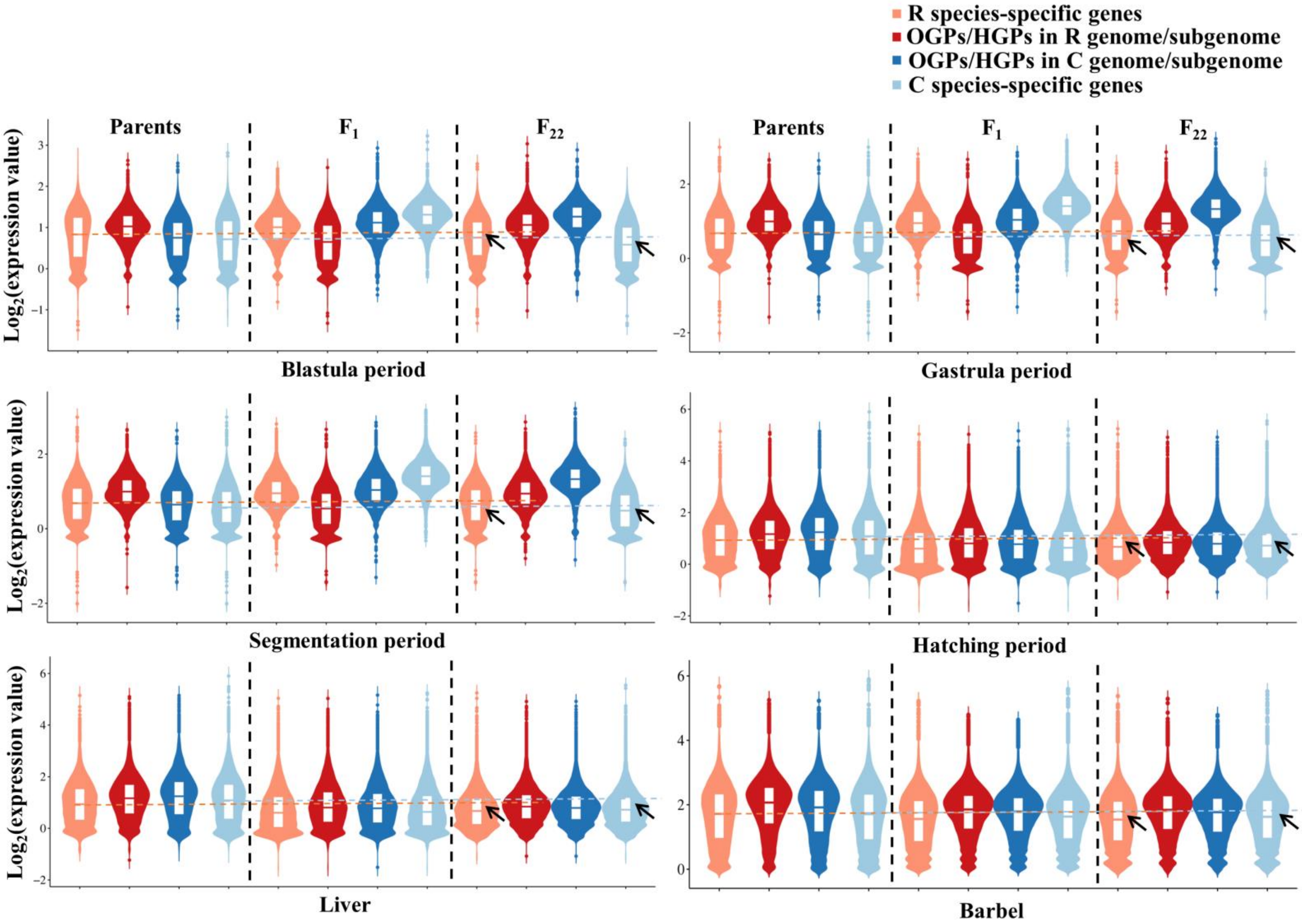


Fig. S25. The gene expression level of OGPs or HGPs and R or C SSGs in goldfish, common carp, $F_1$, and $F_{22}$, respectively. The gene expression levels of OGPs or HGPs were higher than those in SSGs in all comparisons, except

the ones in BA, G, and S periods of $F_1$, in which the gene expression level of OGs was lower than those in SSGs.

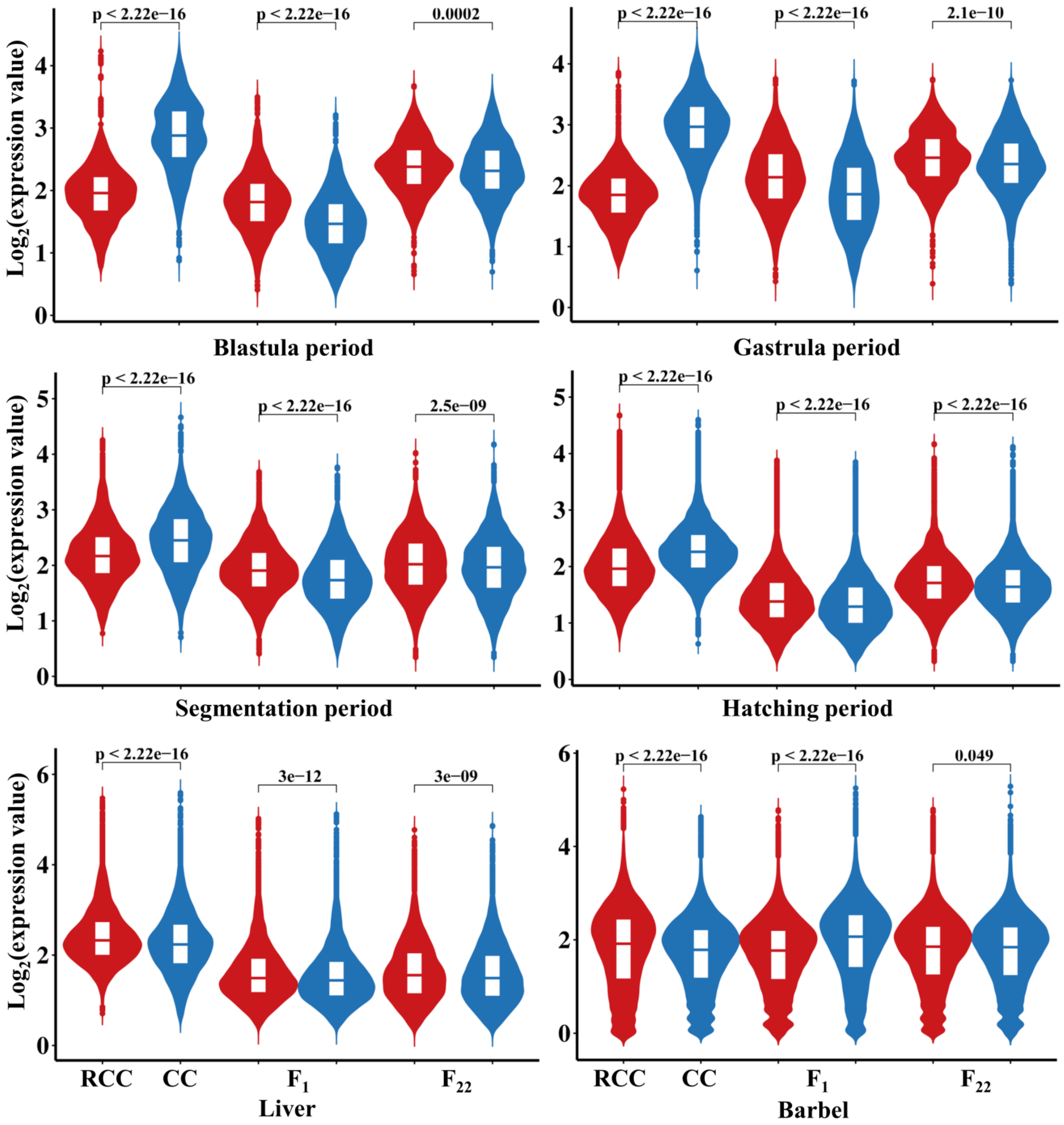


Fig. S26. Differential expression between R (red) and C (blue) homoeologous genes in $F_1$ and $F_{22}$, and orthologous genes in goldfish (red) and common carp (blue), respectively.

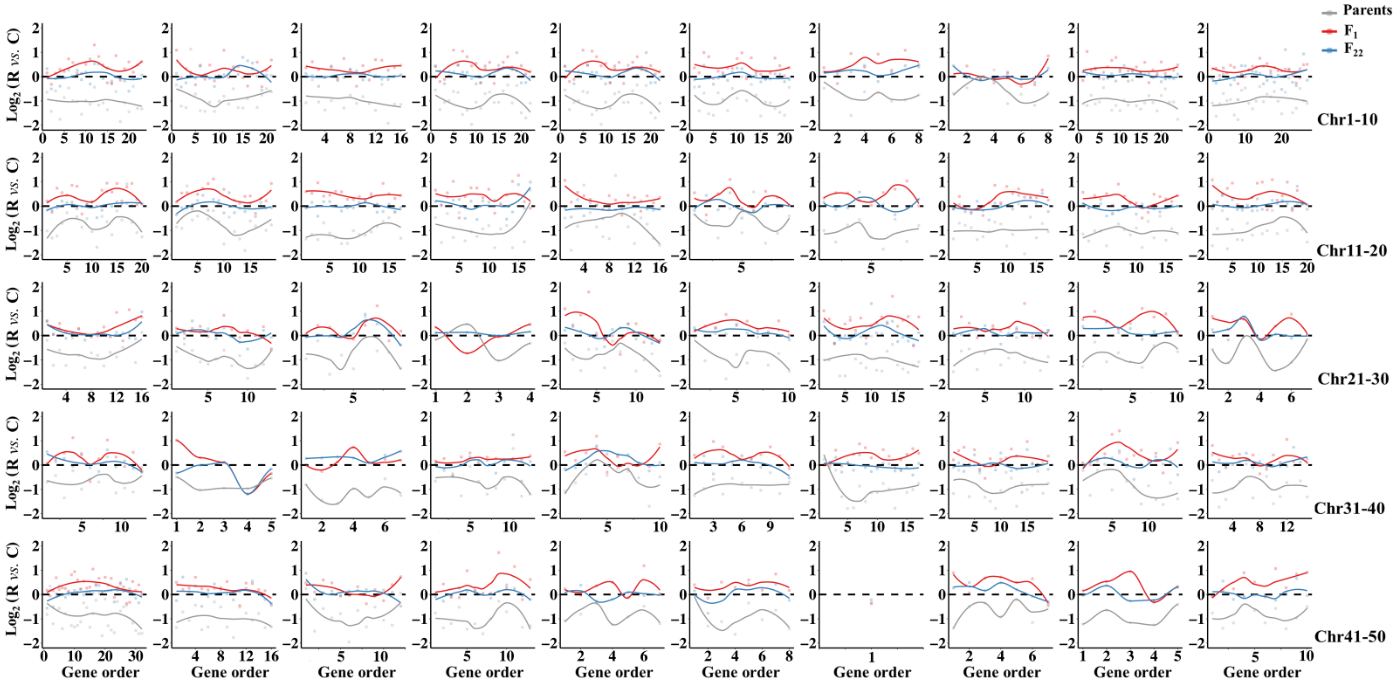


Fig. S27. Homoeolog expression bias (HEB) of blastula period distributed on each chromosome. HEB was detected based on $Log_2$ (R *vs.* C) in parents, $F_1$, and $F_{22}$.

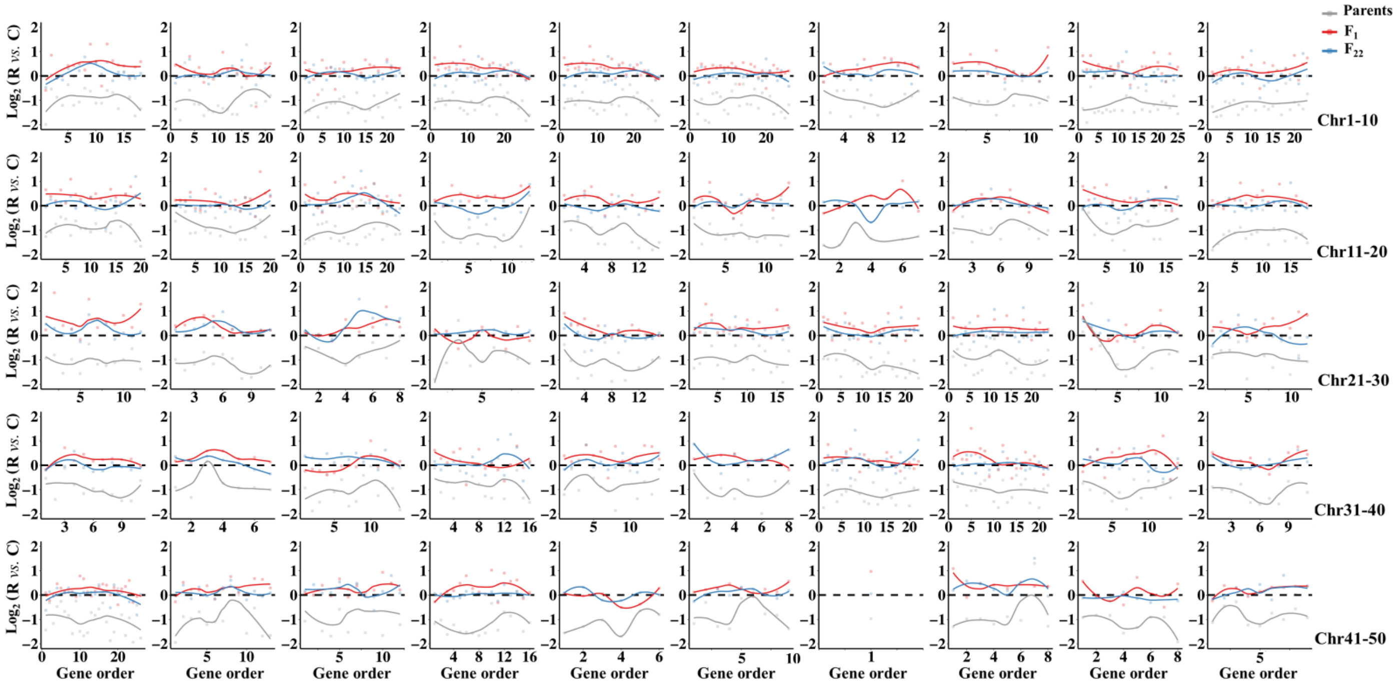


Fig. S28. Homoeolog expression bias (HEB) of gastrula period distributed on each chromosome. HEB was detected based on $Log_2$ (R *vs.* C) in parents, $F_1$, and $F_{22}$.

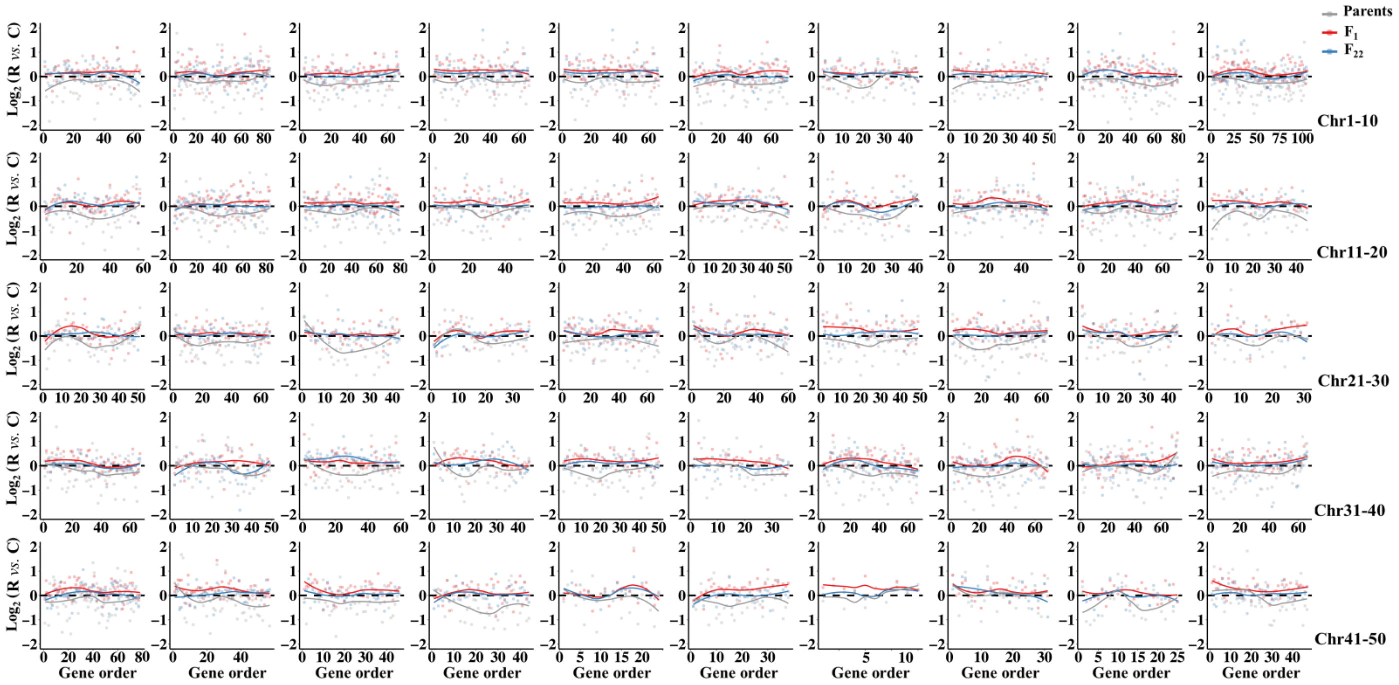


Fig. S29. Homoeolog expression bias (HEB) of segmentation period distributed on each chromosome. HEB was detected based on $Log_2$ (R *vs.* C) in parents, $F_1$, and $F_{22}$.

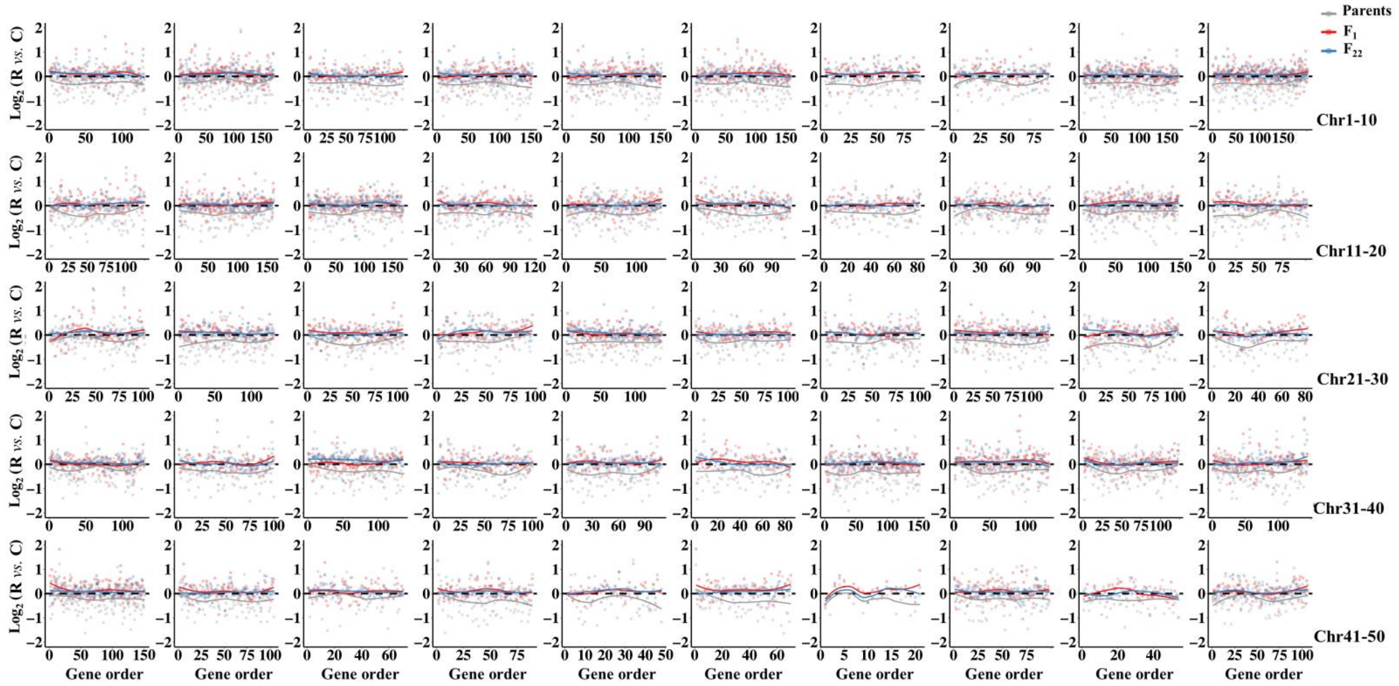


Fig. S30. Homoeolog expression bias (HEB) of hatching period distributed on each chromosome. HEB was detected based on $Log_2$ (R *vs.* C) in parents, $F_1$, and $F_{22}$.

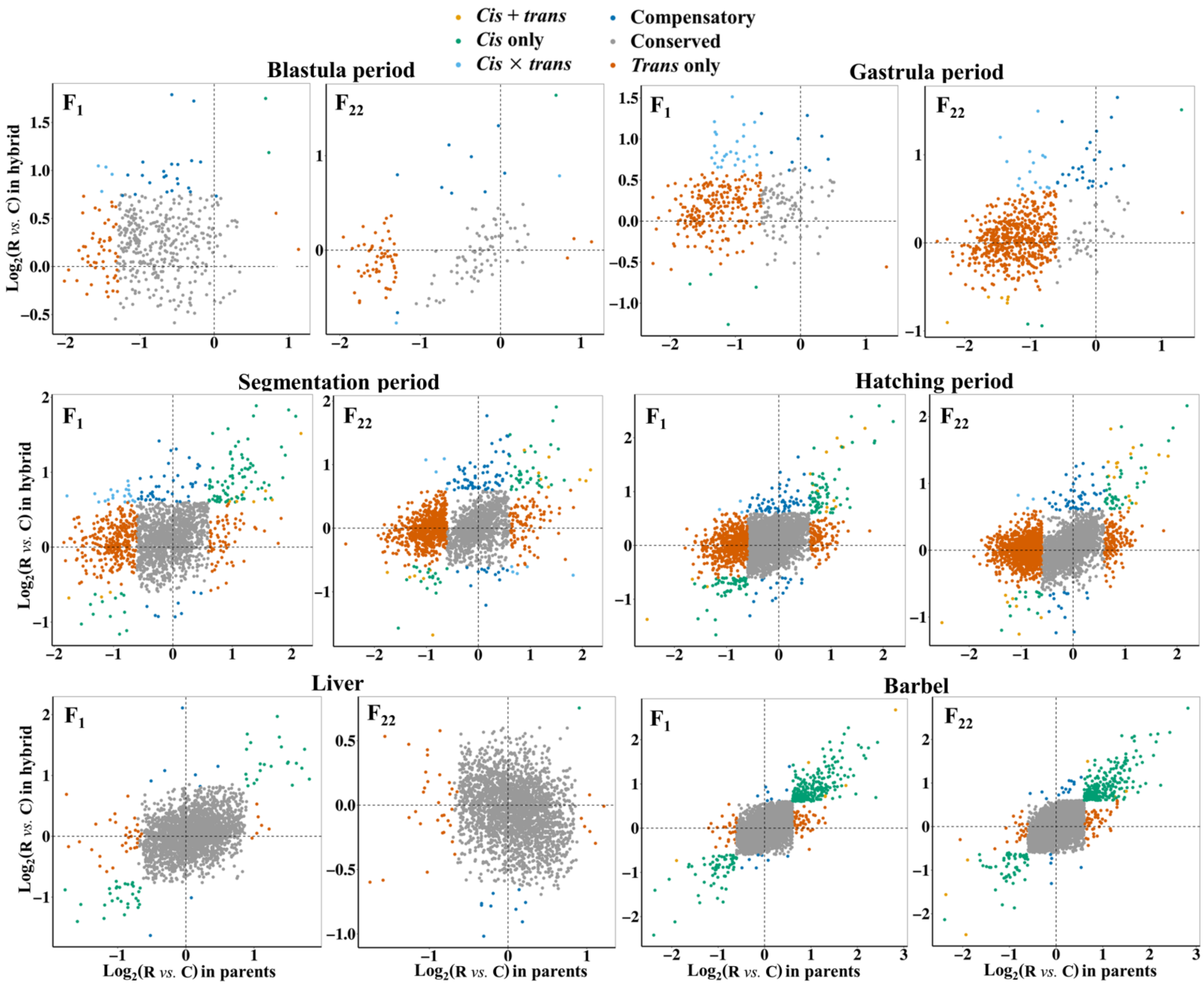


Fig. S31. *Cis*- and *trans*-regulatory genes distributed in different development stages and tissues.

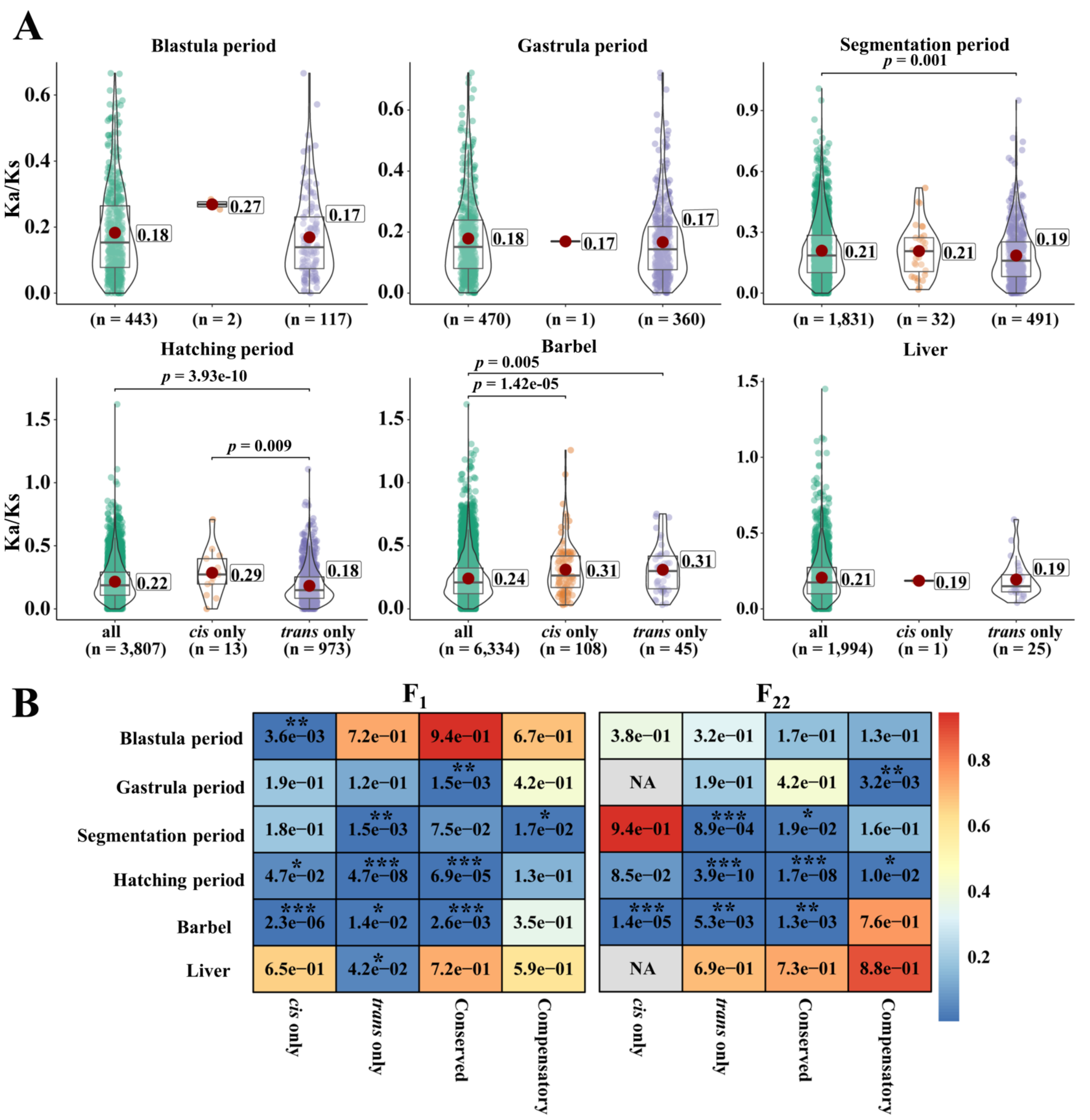


Fig. S32. The analyses of $K_a/K_s$ values in *cis*- and/or *trans*-regulatory patterns. (A) The distribution of $K_a/K_s$ values of OGPs in "*cis* only", "*trans* only", and the total genes (all) of $F_{22}$. A student's *t* test was performed using "ggstatsplot" package. The mean value of the group is signed with a red dot in box. (B) Difference analysis of $K_a/K_s$ values between the genes in four patterns ("*cis* only", "*trans* only", "Conserved", and "Compensatory") and all genes, respectively. *P*-value is signed and described by heat map. The symbol '*' represents the *p*-value < 0.05 in student's *t* test; symbol '**' represents the 0.001< *p*-value < 0.01; symbol '***' represents the *p*-value < 0.001. The symbol 'NA' represents no value.

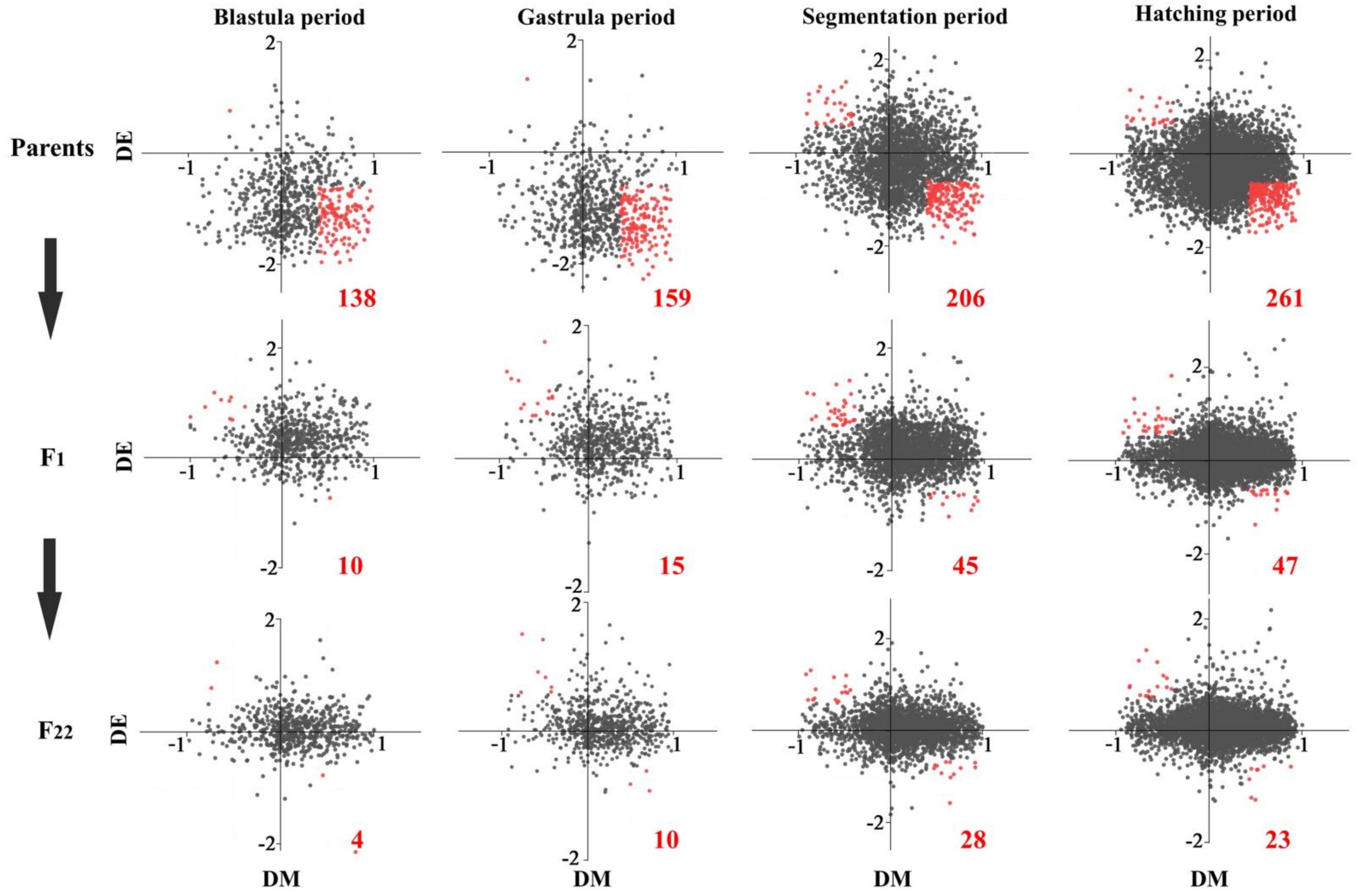


Fig. S33. Gene expression regulated by DNA methylation. Correlation analyses between the gene expression ratios of homoeologs R and C ($\log_2$ (R *vs.* C)) and values of differential methylation (DM). Red dot indicates the negative correlation between values of DE and DM. Black dot indicates the other values of them. The strict thresholds were settled in the analyses with 0.4 in DM and FC = 4 in DE.

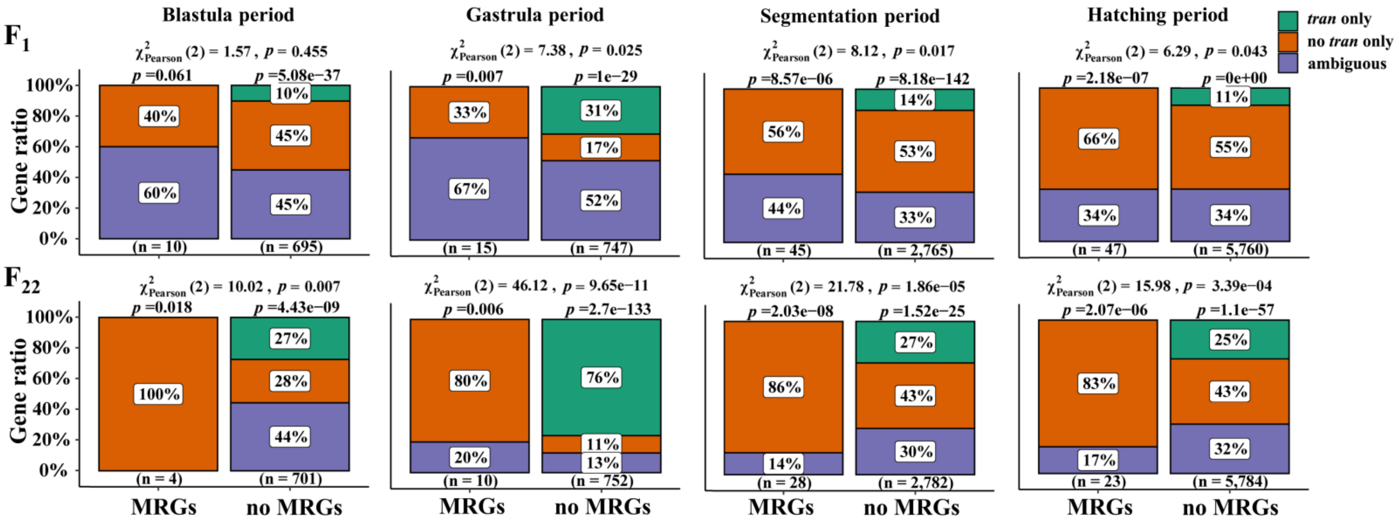


Fig. S34. Correlation analyses of potential DNA methylation-regulated genes (MRGs) and "*tran* only" genes.

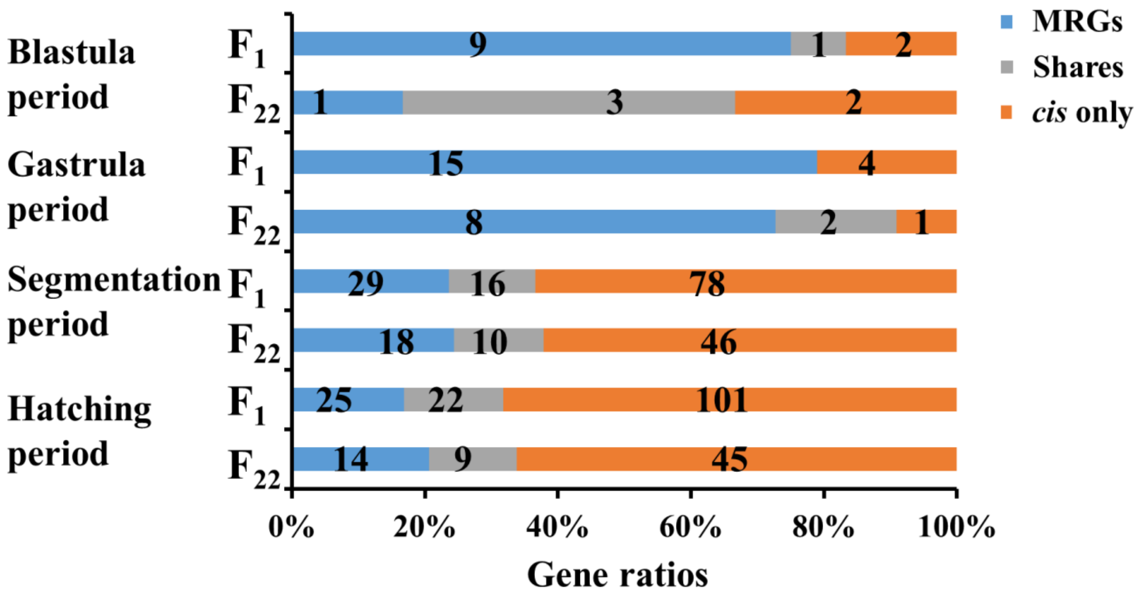


Fig. S35. The distribution of *cis*-regulated genes and potential DNA methylation-regulated genes (MRGs). The "*cis* only" genes were obtained from analysis pipeline of *cis*- and/or *trans*-regulatory patterns, while MRGs were predicted by a negative correlation between DM and DE in hybrids.
